# A Randomized Comparative Trial of Continued Abacavir/Lamivudine plus Efavirenz or Replacement with Efavirenz/Emtricitabine/Tenofovir DF in Hypercholesterolemic HIV-1 Infected Individuals

**DOI:** 10.1371/journal.pone.0116297

**Published:** 2015-02-06

**Authors:** Graeme J. Moyle, Chloe Orkin, Martin Fisher, Jyoti Dhar, Jane Anderson, Edmund Wilkins, Jacqueline Ewan, Ramin Ebrahimi, Hui Wang, for the ROCKET 1 (Randomized Open Label Switch for Cholesterol Elevation on Kivexa Evaluation Trial) Study Group

**Affiliations:** 1 Chelsea and Westminster Hospital, London, SW10 9NH, United Kingdom; 2 St Bartholomew’s and Royal London Hospitals, London, United Kingdom; 3 Brighton and Sussex University Hospitals, Brighton, BN2 5BE, United Kingdom; 4 Leicester Royal Infirmary, Leicester, United Kingdom; 5 Homerton University Hospital, London, United Kingdom; 6 North Manchester General, Manchester, United Kingdom; 7 Gilead Sciences Ltd., Cambridge, United Kingdom; 8 Gilead Sciences Inc, Foster City, California, United States of America; McGill University AIDS Centre, CANADA

## Abstract

**Background:**

Drug choice and metabolic changes with antiretroviral therapy contribute to cardiovascular risk in persons with HIV-1 infection.

**Methods:**

A randomized, 12 week, open-label, comparative study of the impact on lipids of continuation of abacavir/lamivudine (ABC/3TC) plus efavirenz (EFV) or replacement with the single tablet regimen of EFV/emtricitabine/tenofovir DF (EFV/FTC/TDF) in hypercholesterolaemic subjects on successful antiretroviral therapy, with a 12-week extension with all subjects on EFV/FTC/TDF.

**Results:**

157 subjects received study drug, 79 switched to EFV/FTC/TDF and 78 subjects continued ABC/3TC+EFV. At Week 12, 73 subjects on ABC/3TC+EFV switched to EFV/FTC/TDF. The switch was well tolerated and no subject experienced viral rebound. Median baseline fasting total cholesterol was 6.32mmol/L. 12 weeks following switch, the difference in the means (LSM) between treatment groups (EFV/FTC/TDF minus ABC/3TC+EFV) in total cholesterol change from baseline was -0.74mmol/l (95% CI −1.00, −0.47, p < 0.001). The median change from baseline in total cholesterol following switch in the EFV/FTC/TDF arm was -0.86mmol/l (p < 0.001) compared with +0.01mmol/l (p = 0.45) in the continuation arm at Week 12. Significant (p < 0.001) differences between treatment groups following switch were seen for all lipid fractions from baseline to Week 12: LDL cholesterol (−0.47 mmol/L [−0.70, −0.25]), HDL cholesterol (−0.15 mmol/L [−0.21, −0.08]), triglycerides (−0.43 mmol/L [-0.75, -0.11]), and non HDL cholesterol (−0.56 mmol/L [−0.80, −0.31]). In the extension phase, similar declines in total cholesterol were observed with a median change from Week 12 to Week 24 of −0.73mmol/L (p < 0.001).

**Conclusions:**

Switching from ABC/3TC+EFV to EFV/FTC/TDF in persons with hypercholesterolemia maintains virological control and significantly improves key lipid parameters.

**Trial Registration:**

ClinicalTrials.gov NCT00615810

## Introduction

Continuous antiretroviral therapy dramatically reduces HIV-associated morbidity and mortality [1] but may be complicated by adverse effects including metabolic adverse events such as dyslipidemia, and clinical adverse events including cardiovascular events [2]. Adverse events or fear of adverse events remain a key cause of regimen modification, interruption or discontinuation [3].

Comparative clinical data indicate that ABC/3TC is associated with greater increases in total cholesterol and other lipid fractions relative to TDF/FTC-based regimens [4, 5, 6]. Switch data indicate that when replacing a thymidine analogue in persons with lipoatrophy, similar limb fat recovery is observed with ABC or TDF but only TDF leads to declines in lipids [7]. Replacement of zidovudine (AZT)/3TC with TDF/FTC also leads to significant declines in proatherogenic lipids [8]. Healthy volunteer data indicate TDF has a small lipid lowering effect, reducing total cholesterol by 8% over 2 weeks and does not trigger insulin resistance [9].

HIV infection per se appears to be a risk factor for cardiovascular disease [10], and viral suppression may reduce risk of cardiovascular events [11]. Lipid elevation is a key modifiable risk factor for cardiovascular disease. Some cohort data have reported an association between current or recent abacavir use and coronary heart disease (CHD) but not stroke risk [2, 12]. A similar association was not observed with TDF [2]. Differences exist between subjects in randomized trials, who are typically younger, initiating treatment and with few CV risk factors hence fewer events to analyse, compared with the mix of treatment experience, older subjects and more MI events in cohorts. Thus the risk of MI may not be substantially affected by NRTI choice in low risk populations but may exist in older subjects with established MI risk or atheroma.

We sought to investigate the relative benefits on lipids of switching from a virologically successful 2 pill regimen of ABC/3TC +EFV to the single tablet regimen of EFV/FTC/TDF versus continuing with ABC/3TC + EFV for 12 weeks in hyperlipidemic individuals, defined as screening cholesterol of ≥ 5.2 mmol/L (200mg/dL).

## Methods

The protocol for this trial, along with three protocol amendments’ summary of changes, and the supporting CONSORT checklist are available as supporting information (S1 Protocol. Protocol, S2 Protocol. Protocol Amendment 1 — Summary of Changes, S3 Protocol. Protocol Amendment 2 — Summary of Changes, S4 Protocol. Protocol Amendment 3 — Summary of Changes, S1 CONSORT Checklist. CONSORT Checklist).

## Ethics Statement

The study was approved by an appropriate UK Ethics Committee, ‘The Joint UCL/UCLH Committees on the Ethics of Human Research (Committee A)’. The members of the Committee present gave a favourable ethical opinion of the research on the basis described in the application form, protocol and supporting documentation. The approving EC liaised with the local RECs to obtain the outcome of site specific assessments for each of the participating sites. All sites were approved to participate in the study. Patients provided written informed consent prior to study entry. The study was conducted according to Good Clinical Practice guidelines.

This phase IV, open-label, multi-centre, randomized, 12-week trial, with a further 12 week extension phase compared the continuation of ABC/3TC+EFV with switching to the single tablet of EFV/FTC/TDF, in hyperlipidemic subjects on successful antiretroviral therapy. 180 subjects were planned to be enrolled into the study however, recruitment into the study was slower than anticipated, likely in part due to the data becoming available regarding cardiovascular risks with abacavir treatment [2]. Five study sites were added in an attempt to increase recruitment, taking the number of UK HIV treatment centres involved in the study to 17, but after 1 year of recruitment (March 2008 —March 2009) recruitment was stopped with 159 subjects enrolled.

The primary endpoint was the change from baseline in fasting total cholesterol at Week 12. This was assessed at 12 weeks post switch to EFV/FTC/TDF in each arm. Secondary endpoints included changes from baseline in HDL, LDL and triglycerides, calculated Framingham risk, clinical and laboratory safety parameters, HIV-1 RNA and CD4 cell count, patient reported adherence, treatment satisfaction, preference and regimen intrusiveness using validated questionnaires [13] at both Week 12 and 24.

Eligible HIV-1 infected subjects were aged *>*18 years, had been stable on ABC/3TC plus EFV therapy for ≥6 months with no known resistance to any of the study medications. Subjects had documented HIV-1 RNA of < 50 copies/ml at screening and for ≥3 months prior to screening and a screening Total Cholesterol of *>* 5.2mmol/l (200mg/dl) and for the last 2 consecutive tests (at least 4 weeks apart). Subjects receiving lipid lowering therapy were required to be stable for ≥ 3 months prior to screening and were expected to remain on a stable dose and frequency throughout the study. Women of childbearing potential and heterosexual men were required to use an effective method of contraception throughout the study and for up to 12 weeks afterwards. Exclusion criteria included pregnant or lactating females, prior history of significant renal disease or bone disease, creatinine clearance < 60 mL/min, aspartate aminotransferase (AST)/ alanine aminotransferase (ALT) > 5 × upper limit of normal (ULN), and previous FTC, TDF or adefovir dipivoxil therapy. Subjects with active infections, malignancies (except Kaposi Sarcoma or basal cell carcinoma) and resistance to any of the study drugs were also excluded.

The randomization sequence was generated by the lead statistician at Gilead Sciences Inc (Foster City, US) using SAS software version 8.2 and was centralized in block size 4 with no stratification. The centralized randomization list was kept and securely managed, using password protection, by the Clinical Operations team at Gilead Sciences Ltd. (Cambridge, UK). The Investigator at each site completed a Randomization Request Form (RRF) for each subject after protocol eligibility criteria were met and the completed form was faxed to Gilead UK. The subject was randomized according to the randomization schedule and the RRF faxed back to the site with details of the treatment allocation for the subject.

Eligible subjects currently receiving ABC/3TC+EFV were randomized 1:1 to

a) Stop ABC/3TC + EFV and immediately start EFV/FTC/TDF (*immediate switch arm*), or
b) to continue ABC/3TC + EFV and after 12 weeks switch to EFV/FTC/TDF (*delayed switch arm*).

All subjects were then followed to Week 24.

Subjects were followed at baseline and at Weeks 4, 12, 16 and 24 for adverse effects, full blood count, biochemistry, liver and renal function, fasting lipids, HIV-1 RNA and CD4 cell count.

## Statistical methods

**Efficacy.** A modified ITT (MITT) analysis set was used for analyses of fasting lipid parameters. It included subjects who were randomized and received at least 1 dose of study drug and excluded subjects unsuitable for analysis (defined as fasting baseline total cholesterol < 4.2 mmol/L or a non-fasting sample at baseline); subjects were grouped by treatment assigned. The ITT analysis set (subjects who received at least one dose of study drug and grouped by treatment assigned) was used for analyses of HIV‑1 RNA, CD4, and other endpoints. No imputation was used for missing data unless otherwise specified.

The primary endpoint was the change in fasting total cholesterol from baseline to Week 12, in the immediate switch arm, and the change from Week 12 to Week 24, in the delayed switch arm. The primary analysis of the primary endpoint was based on the MITT analysis set and used last post-baseline observation carried forward (Missing = LOCF) methodology. Changes from baseline were analyzed within each treatment group using a Wilcoxon signed rank test, and differences between groups were analyzed using a Wilcoxon rank sum test. Confidence intervals (CIs, 95%) for the differences in changes between groups were constructed based on normal approximation. A secondary analysis of the primary endpoint was based on observed data (i.e., no imputation for missing). Sensitivity analyses were conducted for the primary endpoint using LOCF methodology in the MITT analysis excluding data for subjects who started or modified lipid‑lowering therapy during the study, and using observed data in the treated analysis set. Other fasting lipid parameters were analyzed in a similar manner to the primary endpoint. Percent of subjects by NCEP thresholds were compared between treatment groups using Cochran Mantel Haenszel (CMH) row mean score test. The proportion of subjects with plasma HIV‑1 RNA < 50 and < 400 copies/mL at Week 12, were compared between groups using Fisher exact test. During the study there was a report of an increased frequency of detectable plasma viral load (HIV‑1 RNA) above 50 copies/mL using the COBAS Ampliprep Taqman HIV‑1 test (as employed in this study), compared to the ultrasensitive Ampliprep Amplicor Monitor HIV‑1 test [14]. As recruitment into the study had already been completed when this was discovered, and any detectable viral loads were managed by the sites at the time, the assay was not changed. These important data were communicated to all sites for their information, and the proportion of subjects with HIV‑1 RNA < 200 copies/mL was added to the analysis plan as a secondary endpoint. CD4 and CD8 cell counts, absolute values and change from baseline were summarized by visit. Following a protocol amendment CD4 and CD8 percentages were also added to the analysis plan as secondary endpoints. Differences between EFV/FTC/TDF and ABC/3TC+EFV groups in change from baseline were tested using the Wilcoxon rank sum test at Week 12.

**Safety.** All safety analyses were based on the treated analysis set and were summarized using descriptive statistics by treatment group according to the study drug received. Data collected up to the date of last dose of study drug plus 30 days were included in safety analyses.

**Outcomes Research.** The observed values and changes from baseline at Week 12 in the 10‑year risk for CHD outcomes were summarized by treatment group using the treated analysis set. The change from baseline was tested using the Wilcoxon signed rank test within treatment group and was compared between EFV/FTC/TDF and ABC/3TC+EFV groups using the Wilcoxon rank sum test at Week 12. Other outcomes research endpoints were summarized; changes from baseline were summarized and differences between EFV/FTC/TDF and ABC/3TC+EFV groups were performed using appropriate statistical tests.

**Sample size.** Based on previous studies [7, 8], a mean difference between the two groups of 0.5 mmol/L was expected. A sample size of 180 (90 per group) was expected to provide approximately 85% power to detect a mean difference of 0.5 mmol/L between the two groups in change from baseline to Week 12 in fasting total cholesterol using a two‑sided Student t‑test, assuming that the pooled standard deviation for this difference is estimated to be about 1.1 mmol/L. The number of subjects randomized into the study was lower than planned (159 subjects versus 180 subjects planned). The number of subjects treated in the study (n = 157) provided approximately 80% power to detect a mean difference of 0.5mmol/L.

## Results

### Demographics and Subject Disposition

Of the 177 subjects that were screened for the study, 159 subjects were randomized. 18 subjects were excluded from randomization due to; not meeting the inclusion criteria (15 subjects), declining to participate (one subject) and logistical reasons (two subjects).

Two subjects who were randomized but who did not receive study drug were excluded from all analysis sets, both subjects were in the delayed switch group. All 157 subjects who received study drug (79 subjects in the immediate switch group and 78 subjects in the delayed switch group) were included in the treated and intent‑to‑treat (ITT) analysis sets. Four subjects in the delayed switch group were excluded from the modified ITT (MITT) analysis set in the randomized phase (comparing EFV/FTC/TDF to continuing with ABC/3TC+EFV) for reasons as follows: fasting total cholesterol < 4.2 mmol/L at baseline (one subject) or no fasting baseline measurement (three subjects). The MITT analysis set in the randomized phase comprised 153 subjects (79 subjects in the immediate switch group and 74 subjects in the delayed switch group).

At baseline, subjects were well matched for demographic characteristics as summarized in Table 1. The median baseline CD4 cell count in the immediate switch group was 459 cells/mm^3^ (IQR: 377,604) and in the delayed switch group was 450 cells/mm^3^ (IQR: 371,584). The proportion of subjects with a baseline HIV RNA < 50 copies/ml was 96.2% and 92.2% respectively, all subjects having been < 50 copies/ml at screening.

**Table 1:** Demographics and Baseline Characteristics (Treated Analysis Set).

	**Immediate SwitchEFV/FTC/TDF**	**Delayed Switch (Continue ABC/3TC + EFV for 12 Weeks)**
Number of Subjects	79	78
Median age in yrs (IQR)	42 (36, 48)	44 (40, 50)
Race		
White	45 (57.0%)	48 (61.5%)
Black	29 (36.7%)	27 (34.6%)
Asian	2 (2.5%)	0
Other	3 (3.8%)	3 (3.9%)
Gender		
Male	61 (77.2%)	64 (82.1%)
HIV RNA		
< 50 copies/mL	76/79 (96.2%)	71/77 (92.2%)
< 400 copies/mL	79/79 (100%)	77/77 (100%)
Median CD4 (cells/microl) (IQR)	459 (377, 604)	450 (371, 584)
Median BMI (kg/m2) (IQR)	25.7 (23.5, 29.3)	25.8 (23.7, 28.0)
Median Fasting TC (mmol/L) ( IQR)	6.62 (5.97, 7.26)	6.19 (5.80, 6.78)
Number of Subjects on Prior Lipid Modifying Agents	9 (11.4%)	13 (16.7%)

**Figure 1:**
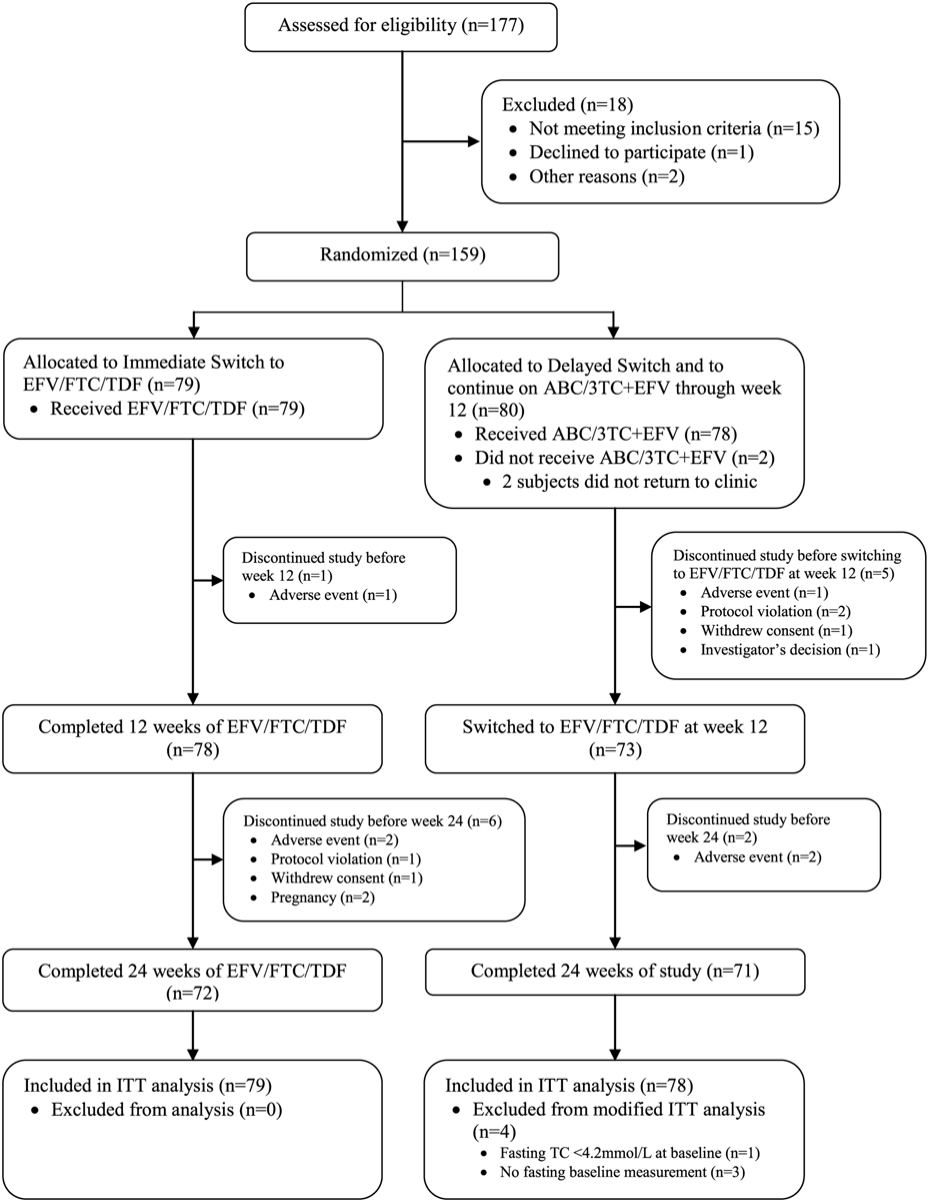
Flow diagram of progress through the study phases (All Subjects).

157 subjects received at least one dose of study drug. 143 subjects completed 24 weeks of study treatment and 14 subjects discontinued the study before 24 weeks, seven in the immediate switch arm, five in the continuation arm and two following delayed switch. The study flow of the progress through the study is shown in Fig. 1 [15, 16]. The reasons for discontinuation were adverse events (six subjects), protocol violation (three subjects), pregnancy (two subjects), withdrawal of consent (two subjects), and investigator’s decision (one subject). Subject disposition is shown in Table 2.

**Table 2:** Subject Disposition at Week 24 (Treated Analysis Set).

	**Immediate Switch**		**Delayed Switch**	
**N (%)**	**EFV/FTC/TDF (N = 79)Baseline—Wk 12**	**EFV/FTC/TDF (N = 79) Wk 12 —Wk 24**	**ABC/3TC + EFV (N = 78) Baseline—Wk 12**	**EFV/FTC/TDF (N = 73) Wk 12 —Wk 24**
Subjects completing study treatment	78 (98.7%)	72 (91.1%)	73 (93.6%)	71 (91.0%)
Early Treatment Discontinuation	1 (1.3%)	6 (7.6%)	5 (6.4%)	2 (2.7%)
Adverse Events *	1 (1.3%)	2 (2.5%)	1 (1.3%)	2 (2.7%)
Pregnancy	0	2 (2.5%)	0	0
Protocol Violation	0	1 (1.3%)	2 (2.5%)	0
Withdrew Consent	0	1 (1.3%)	1 (1.3%)	0
Investigator’s Decision	0	0	1 (1.3%)	0

*Adverse events leading to study drug discontinuation:
Immediate Switch: emergent to EFV/FTC/TDF—anxiety; insomnia; night sweats Delayed Switch: emergent to ABC/3TC + EFV (baseline to Wk12)—depression Delayed Switch: emergent to EFV/FTC/TDF (Wk 12 to Wk 24)—sleep disorder; urticaria
The proportion of subjects discontinuing due to adverse events at week 12 was the same in both groups (n = 1; 1.3%)

### Primary Endpoint

**Figure 2:**
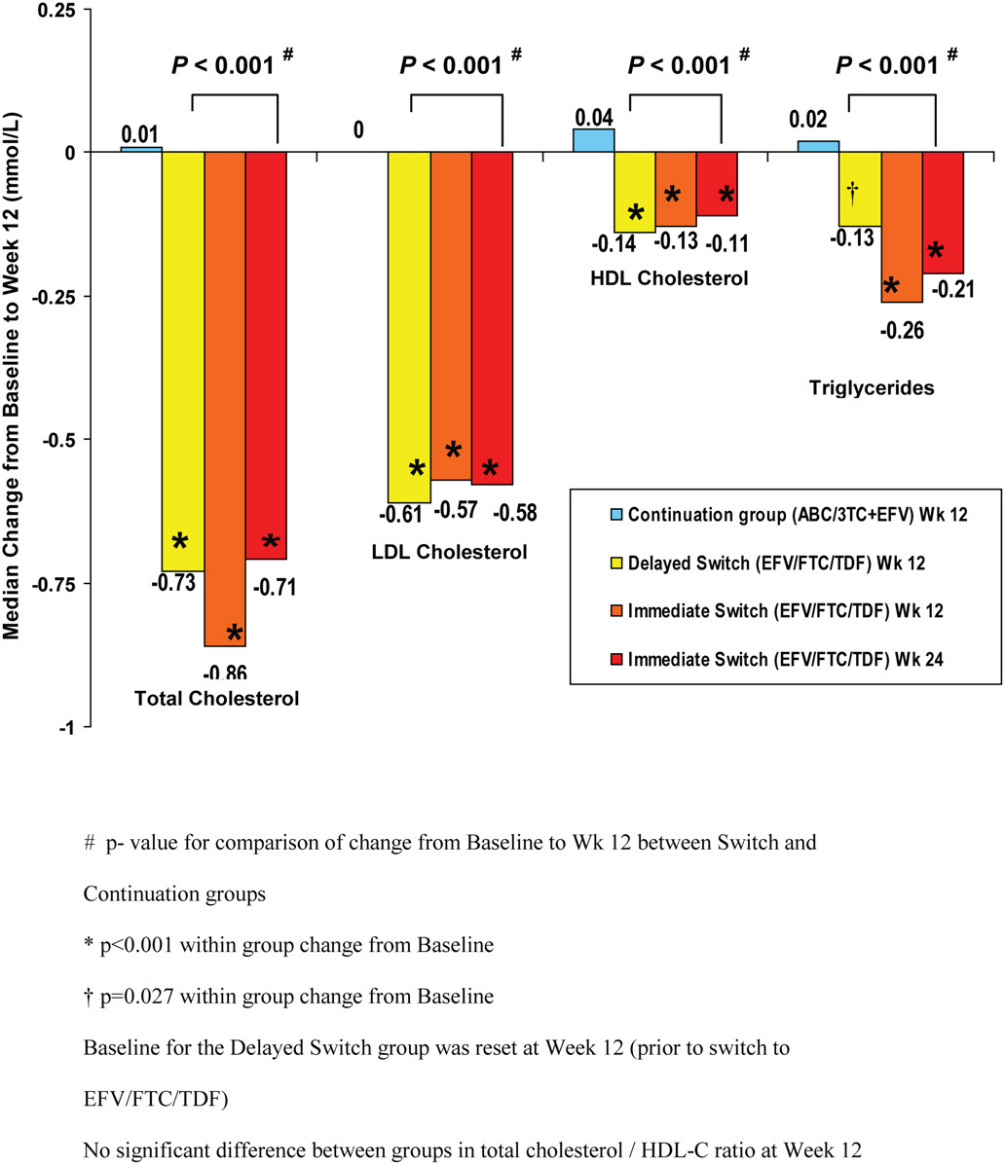
Changes in Lipid fractions from Baseline (Treated Analysis Set). Significant declines from baseline were seen in the Immediate Switch group but not in the Delayed Switch group.

**Figure 3:**
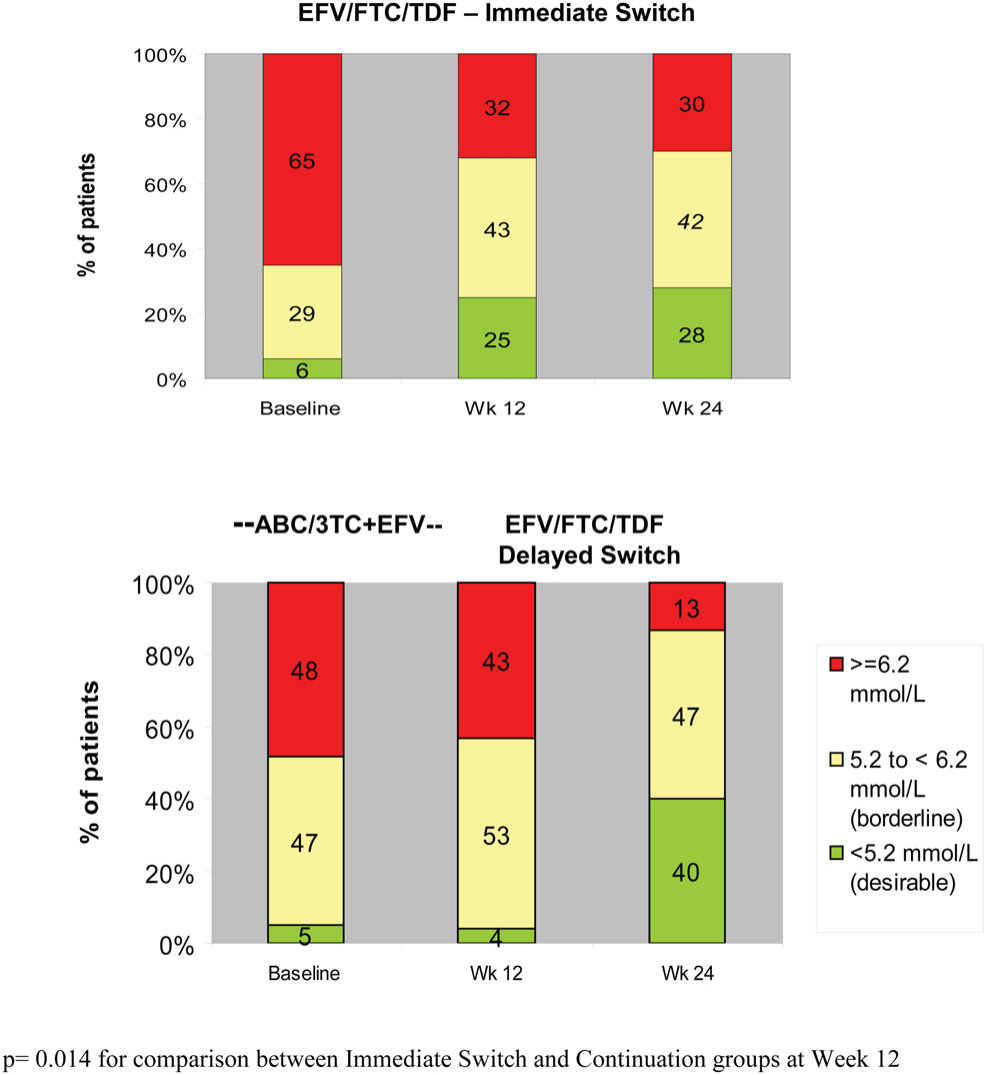
Fasting Total Cholesterol by NCEP Thresholds (Treated Analysis Set).

At Study Week 12, in the immediate switch group, there was a statistically significant decrease from baseline in fasting total cholesterol (median change −0.86 mmol/L, p < 0.001), while in the delayed switch group (subjects continuing with ABC/3TC+EFV) there was no statistically significant change from baseline to Week 12 in fasting total cholesterol (median change 0.01 mmol/L, p = 0.45) (Table 3). Similar to the effect observed in the immediate switch group, there was a statistically significant decrease 12 weeks post-switch to EFV/FTC/TDF (i.e. from study Week 12 to Week 24) in fasting total cholesterol in the delayed switch group (median change −0.73 mmol/L, p < 0.001) (Fig. 2). Results were similar using sensitivity analyses. The difference in the means (LSM) between groups (EFV/FTC/TDF minus ABC/3TC+EFV) for the change from baseline to Week 12 was −0.74 mmol/L (95% CI −1.00, −0.47); this difference was statistically significant (p < 0.001). These favourable changes in total cholesterol following switch to EFV/FTC/TDF led to fewer subjects being above NCEP (National Cholesterol Education Program) treatment thresholds, (http://www.nhlbi.nih.gov/guidelines/cholesterol/index.htm) (Fig. 3).

**Table 3:** Change from Baseline at Week 12 in Fasting Lipids (Modified Intent To Treat Analysis Set.

	**Immediate Switch**	**Delayed Switch**			
**Change From Baseline 12 Weeks post-switch**	**EFV/FTC/TDF (N = 79)**	**ABC/3TC+EFV(N = 74)**	**Delayed Switch to EFV/FTC/TDF^a^ (N = 73)**	**p-value^b^**	**Diff in LSM (95% CI)^c^**
Fasting Total Cholesterol (mmol/L)					
N	79	73	68	<.001	−0.74 (−1.00, −0.47)
Median	−0.86	0.01	−0.73		
p-value^d^	<.001	0.45	<.001		
LDL Cholesterol (mmol/L)					
N	79	73	68	<.001	−0.47 (−0.70, −0.25)
Median	−0.57	0.00	−0.61		
p-value^d^	<.001	0.47	<.001		
HDL Cholesterol (mmol/L)					
N	78	73	68	<.001	−0.15 (−0.21, −0.08)
Median	−0.13	0.04	−0.14		
p-value^d^	<.001	0.44	<.001		
Triglycerides (mmol/L)					
N	79	73	68	<.001	−0.43 (−0.75, −0.11)
Median	−0.26	0.02	−0.13		
p-value^d^	<.001	1.00	0.063		

^a^Delayed Switch to EFV/FTC/TDF column includes subjects who were randomized to continue ABC/3TC+EFV at baseline and had at least 1 dose of EVF/FCT/TDF after switch at Week 12.

^b^The p-value for comparison between EFV/FTC/TDF and ABC/3TC +EFV at study Week 12 is from Wilcoxon rank sum test.

^c^The 95% confidence interval for the difference (EFV/FTC/TDF vs. ABC/3TC+EFV) is based on normal approximation.

^d^The p-value for within treatment group comparison is from Wilcoxon signed rank test.

### Lipids fractions

Results of analyses of secondary fasting lipid parameters were similar to those for fasting total cholesterol. For each parameter there was a statistically significant decrease from baseline to Week 12 in the immediate switch to EFV/FTC/TDF group but no significant change from baseline to Week 12 while subjects continued on ABC/3TC+EFV in the delayed switch group. Differences between groups (mean (95% CI)) for the changes from baseline to Week 12 in secondary fasting lipid parameters were statistically significant (p < 0.001) for LDL cholesterol (−0.47 mmol/L (−0.70, −0.25)), HDL cholesterol (−0.15 mmol/L (−0.21, −0.08)), triglycerides (−0.43 mmol/L (−0.75, −0.11)), and non‑HDL cholesterol (−0.56 mmol/L (−0.80, −0.31)), but not for total/HDL cholesterol ratio. Results in the delayed switch group at Week 24 were similar to those in the immediate switch group at Week 12 (Fig. 2).

### HIV Disease Markers

No subjects met protocol‑defined criteria for virologic failure (2 consecutive HIV‑1 RNA values ≥ 400 copies/mL). High rates of ITT virologic suppression were maintained in subjects after switching therapy and while they remained on ABC/3TC+EFV. At study Week 12, there were no statistically significant differences between the immediate and delayed switch groups in the proportions of subjects with HIV‑1 RNA < 50 copies/ml (92.2% vs. 90.3%, respectively).

Baseline median CD4 count values (cells/μL) were 459 and 450 for the immediate and delayed switch groups, respectively. At Week 12, median changes from baseline were −25 cells/μL and 27 cells/μL, for the immediate and delayed switch groups, respectively (between treatment groups p = 0.013). In the delayed switch group, 12 weeks after initiating EFV/FTC/TDF (study Week 12 to Week 24), the median change in CD4 cell count was 25 cells/μL.

### 10-Year Risk for Coronary Heart Disease Outcomes

Changes from baseline to Week 12 in the 10‑year risk for CHD outcomes were not statistically significant within any treatment group or between randomized treatment groups. Mean (SD) changes from baseline at study Week 12 were—0.6 (3.85) in the immediate switch group and—0.1 (2.69) in the delayed switch group (while continuing ABC/3TC+EFV).

### Treatment Adherence—Visual Analog Scale (VAS)

Study drug adherence based on VAS assessments was high (median ≥ 98.0%) in all groups at baseline. There were no statistically significant differences in the reported VAS adherence between immediate switch and delayed switch groups, and no statistically significant changes from baseline in VAS adherence in any group.

### Treatment Satisfaction

Treatment satisfaction and regimen tolerability improved after switching to EFV/FTC/TDF. At study Week 12, statistically significant differences between the immediate switch group and delayed switch group (while continuing on ABC/3TC+EFV) were observed for satisfaction with convenience and simplicity (very satisfied: 90.7% EFV/FTC/TDF; 76.7% ABC/3TC+EFV), and the ability to tolerate the regimen (very satisfied: 81.1% EFV/FTC/TDF; 61.6% ABC/3TC+EFV). In addition, there was a statistically significant difference between groups in the proportion of subjects who were bothered by side effects of the regimen at study Week 12 (bothered: 39.2% immediate switch; 61.6% delayed switch). As might be expected in this virologically stable study population, there were no significant differences between groups in satisfaction with the ability of the regimen to control HIV, or with general satisfaction of the regimen.

### Perceived Ease of Regimen for Condition (PERC)

Subjects perceived the single tablet regimen to be easier to follow than the 2 pill regimen, as assessed using the PERC survey. In the All EFV/FTC/TDF group (subjects from both the immediate and delayed switch groups) the proportion of subjects who considered their regimen very easy to take increased from 78.9% at baseline to 90.1% 12 weeks after switching (p = 0.004). However, the difference between the immediate switch and delayed switch groups at Week 12 did not achieve statistical significance (very easy: 90.5% EFV/FTC/TDF vs. 80.3% ABC/3TC+EFV, p = 0.10).

### Preference of Medicine

Subjects in the All EFV/FTC/TDF group generally preferred the single tablet regimen. Following switch, 54.5% of subjects considered EFV/FTC/TDF to be much better than their previous regimen and 14.3% of subjects considered EFV/FTC/TDF to be slightly better than their previous regimen (p < 0.001).

### HAART Intrusiveness Scale

Subjects in the All EFV/FTC/TDF group perceived diminished regimen intrusiveness following switch with a statistically significant reduction (improvement) in overall m‑HIS index score from baseline to Week 12 (median (IQR) change 0.0 (−0.2, 0.0), p < 0.001). The difference between the immediate switch and delayed switch groups at study Week 12 was statistically significant (median (IQR) changes were 0.0 (−0.1, 0.0) and 0.0 (0.0, 0.1), respectively, (p = 0.037)).

### Adverse Events

Adverse events (AEs) considered related to study drug by the investigator were reported for 30.3% of subjects following switch to EFV/FTC/TDF (46 subjects (25 immediate switch through to study Week 24, 21 delayed switch from study Week 12 to 24)) and for 3.8% of subjects (3 subjects) while subjects continued on ABC/3TC+EFV (baseline to study Week 12). After switching to EFV/FTC/TDF, the most frequently reported AEs considered related to study drug were abnormal dreams (12 subjects (7 immediate switch, 5 delayed switch)) and fatigue (8 subjects (2 immediate switch, 6 delayed switch)). Five subjects discontinued study drug due to AEs following switch (3 immediate switch, 2 delayed switch) and 1 subject while continuing ABC/3TC+EFV. There were no Serious Adverse Events (SAEs) related to study drug reported for either arm. No AE considered related to study drug was reported for more than 1 subject while on ABC/3TC+EFV. No cardiovascular events were observed.

### Renal Function

Renal function was assessed by performing urinalysis and calculating creatinine clearance at every visit throughout the study. Estimated creatinine clearance (eCrCl) and glomerular filtration rate (eGFR) was calculated using the Cockcroft-Gault (CG) and Modified Diet in Renal Disease (MDRD) methods respectively, and there were no meaningful or significant changes seen in either arm during the study.

Treatment-emergent proteinuria was reported for 11 subjects (all Grade 1) in the immediate switch group through 24 weeks and for 11 subjects (Grade 1 for 7 subjects and Grade 2 for 4 subjects) in the delayed switch group while on ABC/3TC+EFV through 12 weeks. After switching to EFV/FTC/TDF, treatment-emergent proteinuria was reported for 12 subjects (all Grade 1) in the delayed switch group. There were no clinically relevant changes in median values for urine protein/creatinine ratio within treatment groups, and no clinically relevant differences between randomized treatment groups. No AEs were reported in relation to urine protein abnormalities. No subjects discontinued the study due to renal adverse events.

## Discussion

Prevention and management of drug-related adverse events and maintenance of adherence remain key challenges to the success of long-term antiretroviral therapy. This study documents several benefits of switching from ABC/3TC+EFV to the single tablet regimen of EFV/FTC/TDF in persons with dyslipidemia. These advantages include significant declines in pro-atherogenic lipids and improved perception of their treatment regimen. These benefits are gained without loss of virological control and have a low risk of introducing new unexpected adverse experiences.

Prospective studies in initial therapy [4–6] and switch from thymidine analogue [7, 8] suggest TDF based regimens have more limited impact on lipids than other NRTIs and may have a small lipid lowering effect. In healthy volunteers total and non-HDL cholesterol are reduced by two weeks with TDF and no impact on glucose disposal is observed [9]. The mechanism by which TDF reduces cholesterol has not been investigated. Changes observed in cholesterol in this study are similar to those reported with less potent statin agents [17] and led to a trend of fewer subjects being in NCEP treatment ranges.

While renal dysfunction has been reported in individuals receiving tenofovir DF [18], no differences in changes in renal function were observed. These findings are consistent with prospective randomized controlled studies in treatment-naïve individuals [8, 19].

The subject preference of the single tablet regimen and the lower perceived intrusiveness of a regimen that involved the same dosing interval and only one less pill is consistent with previous switch studies that have demonstrated similar benefits in switching from twice to once daily therapies with these and other validated tools [13, 20–22]. These data provide support for the development of once-daily single tablet regimens as tools that may support long term patient adherence and treatment persistency.

The study has some limitations, predominately that it is of a short term nature and did not include detailed assessments of other clinical and biomarkers of CV risk other than lipids. Changes in lipids observed with therapy switching may not have the same benefits on CV risk as lipid reductions with statin agents due to the pleiotropic effects of these agents [23] and there have been studies that do not support the association between abacavir (ABC) exposure and increased risk of myocardial infarction (MI) among HIV-infected individuals [24, 25].

In summary, switching from ABC/3TC+EFV to the single tablet regimen of EFV/FTC/TDF leads to statistically significant declines in total cholesterol and other proatherogenic lipid fractions and improved patient preference. Regimen switch to EFV/FTC/TDF can be safely achieved without a significant risk of loss of virologic control or introduction of new unexpected adverse events.

## Supporting Information

S1 CONSORT Checklist (DOC)

S1 Protocol Protocol. (PDF)

S2 Protocol Protocol Amendment 1—summary of changes. (PDF)

S3 Protocol Protocol Amendment 2—summary of changes. (PDF)

S4 Protocol Protocol Amendment 3—summary of changes. (PDF)
