# Supplementary material for: A Randomized Comparative Trial of Continued Abacavir/Lamivudine plus Efavirenz or Replacement with Efavirenz/Emtricitabine/Tenofovir DF in Hypercholesterolemic HIV-1 Infected Individuals
Source: PLoS One. 2015 Feb 6;10(2):e0116297. doi: 10.1371/journal.pone.0116297 (PMC4319732; doi:10.1371/journal.pone.0116297)
Supplement: S1 Protocol — (PDF) [file pone.0116297.s002.pdf]

CLINICAL STUDY PROTOCOL

Study Title:	**ROCKET** - **R**andomized **O**pen Label Switch for **C**holesterol Elevation on **K**ivexa **E**valuation **T**rial<br><br>A Phase 4, Open Label, Randomized, Controlled Study to Assess the Effect on Lipid Profile of Switching from a Stable HAART Regimen of fixed dose Abacavir/Lamivudine (Kivexa) Plus Efavirenz, to Once Daily Atripla in Adult HIV-1 Infected Subjects With Raised Cholesterol
Sponsor:	Gilead Sciences Limited<br>Granta Park, Abington<br>Cambridge, CB21 6GT<br>United Kingdom
IND Number<br>EudraCT Number	Not applicable<br>2007-003354-28
Indication:	HIV-1 Infection
Protocol ID:	GS-UK-177-0109
Gilead Sciences Medical Monitor:	Name: Dr Cham Herath<br>Telephone: +44 (0) 1223 897300<br>Fax: +44 (0) 1223 897281<br>E-mail: cham.herath@gilead.com
Protocol Version/Date:	Final: 22 November 2007

CONFIDENTIALITY STATEMENT

The information contained in this document, particularly unpublished data, is the property or under control of Gilead Sciences, Inc., and is provided to you in confidence as an investigator, potential investigator, or consultant, for review by you, your staff, and an applicable Independent Ethics Committee. The information is only to be used by you in connection with authorized clinical studies of the investigational drug described in the protocol. You will not disclose any of the information to others without written authorization from Gilead Sciences Limited, except to the extent necessary to obtain informed consent from those persons to whom the drug may be administered.

TABLE OF CONTENTS

1. INTRODUCTION 11
1.1. Background and Rationale for the Current Study 11
1.2. EFV/FTC/TDF Fixed-Dose Combination Tablets 12
1.3. Other Study Drugs 12
2. OBJECTIVES 13
3. STUDY DESIGN 14
3.1. Treatment Plan and Regimen 14
4. SUBJECT POPULATION 16
4.1. Number of Subjects and Subject Selection 16
4.2. Inclusion Criteria 16
4.3. Exclusion Criteria 17
5. STUDY DRUGS 19
5.1. Randomization and Blinding 19
5.2. Atripla Tablets 19
5.2.1. Formulation 19
5.2.2. Packaging and Labeling 19
5.2.3. Storage and Handling 20
5.2.4. Dosage and Administration 20
5.3. Kivexa (ABC/3TC Tablets) 20
5.3.1. Formulation 20
5.3.2. Packaging and Labeling 20
5.3.3. Storage and Handling 20
5.3.4. Dosage and Administration 20
5.3.5. Further Information 20
5.4. Efavirenz Tablets (Sustiva®) 21
5.4.1. Formulation 21
5.4.2. Packaging and Labeling 21
5.4.3. Storage and Handling 21
5.4.4. Dosage and Administration 21
5.4.5. Further Information 21
5.5. Prior and Concomitant Medications 21
6. STUDY PROCEDURES 22
6.1. Subject Enrollment and Treatment Assignment 22
6.2. Pretreatment Assessments 22
6.2.1. Screening Visit 22
6.2.2. Baseline Assessments 23
6.3. Treatment Assessments 24
6.4. Unscheduled Visit 25
6.5. 30 Day Follow-up Assessment 25
6.6. Assessments for Premature Discontinuation from Study 25
6.7. Criteria for Discontinuation of Study Treatment 26
7. ADVERSE EVENTS AND TOXICITY MANAGEMENT 27
7.1. Adverse Events 27
7.2. Assessment of Adverse Events 28

7.3. Serious Adverse Events 28
7.4. Overdose 30
7.5. Serious Adverse Event Reporting Requirements 30
7.5.1. All Serious Adverse Events 30
7.5.2. Investigator and Sponsor Reporting Requirements for SAEs 31
7.5.3. Poststudy Reporting Requirements 31
7.6. Clinical Laboratory Abnormalities and Other Abnormal Assessments as Adverse Events or Serious Adverse Events 32
7.7. Toxicity Management 32
7.7.1. Grades 1 and 2 Laboratory Abnormality or Clinical Event 33
7.7.2. Grade 3 Laboratory Abnormality or Clinical Event 33
7.7.3. Grade 4 Laboratory Abnormality or Clinical Event 33
7.8. Risks for Women of Childbearing Potential or During Pregnancy 34

8. STATISTICAL CONSIDERATIONS 36

8.1. Analysis Objectives 36
8.2. Primary Endpoint 36
8.3. Secondary Endpoint 36
8.4. Methods of Analysis 37
8.4.1. Analysis Sets 37
8.4.2. Data Handling Conventions 37
8.4.3. Interim Analysis 38
8.5. Demographic Data and Baseline Characteristics 38
8.6. Analysis Methods 38
8.6.1. Analyses of Primary and Efficacy Endpoints 38
8.6.2. Analysis of Secondary Endpoints 39
8.6.3. Extent of Exposure 39
8.6.4. Adverse Events 39
8.6.5. Laboratory Evaluations 39
8.6.6. Other Safety Evaluations 40
8.7. Sample Size 40

9. RESPONSIBILITIES 41

9.1. Investigator Responsibilities 41
9.1.1. Good Clinical Practice 41
9.1.2. Independent Ethics Committee (IEC) Approval 41
9.1.3. Informed Consent 41
9.1.4. Confidentiality 41
9.1.5. Study Files and Retention of Records 42
9.1.6. Electronic Case Report Forms 42
9.1.7. Drug Accountability 43
9.1.8. Inspections 43
9.1.9. Protocol Compliance 43
9.2. Sponsor Responsibilities 43
9.2.1. Protocol Modifications 43
9.2.2. Study Report and Publications 44
9.3. Joint Investigator/Sponsor Responsibilities 44
9.3.1. Access to Information for Monitoring 44
9.3.2. Access to Information for Auditing or Inspections 45
9.3.3. Study Discontinuation 45

10. REFERENCES 46

11. APPENDICES 48

Appendix 1. Investigator Signature Page 49
Appendix 2. Study Procedures Table 50
Appendix 3. Management of Clinical and Laboratory Adverse Events 52
Appendix 4. GSI Grading Scale for Severity of Adverse Events and Laboratory Abnormalities......53
Appendix 5. Patient Preference of Medicine (POM) 76
Appendix 6. Treatment Satisfaction and Symptoms Questionnaire 77
Appendix 7. Disallowed Medications 79
Appendix 8. HAART Intrusiveness Scale (m-HIS) 81
Appendix 9. Perceived Ease of Adherence (PERC) Questionnaire 82
Appendix 10. National Cholesterol Education Program ATP III Guidelines 84
Appendix 11. World Medical Association Declaration of Helsinki 85

LIST OF IN-TEXT FIGURES

Figure 3-1. Study Schema 15

PROTOCOL SYNOPSIS

Gilead Sciences Limited
Granta Park, Abington
Cambridge, CB21 6GT
United Kingdom

**Study Title:**	**ROCKET** - **R**andomized **O**pen Label Switch for **C**holesterol Elevation on **K**ivexa **E**valuation **T**rial<br><br>A Phase 4, Open Label, Randomized, Controlled Study to Assess the Effect on Lipid Profile of Switching from a Stable HAART Regimen of fixed dose Abacavir/Lamivudine (Kivexa) Plus Efavirenz, to Once Daily Atripla in Adult HIV-1 Infected Subjects With Raised Cholesterol
**IND Number:**	This is a non-IND study.
**EudraCT Number:**	2007-003354-28
**Study Centers Planned:**	Approximately 10 centers in the United Kingdom
**Objectives:**	The primary objective is to determine if switching from a stable HAART regimen of Kivexa® + EFV to once daily Atripla® leads to a reduction in total fasting cholesterol at 12 weeks.<br><br>The secondary objectives of this study are<br>• Evaluation of fasting metabolic parameters (e.g., LDL, HDL, triglycerides, non-HDL cholesterol and cholesterol ratios).<br>• Evaluation of efficacy and safety by assessing adverse events, clinical laboratory tests, physical examinations and vital signs at every visit.<br>• Evaluation of changes in the 10-year risk factor for coronary heart disease outcomes as measured by total cholesterol, HDL, blood pressure, smoking status, treatment for hypertension, sex and age.

**Study Design:**	Phase 4, open label, randomized, controlled study to assess the effect on lipid profile of switching from a stable HAART regimen of Kivexa + EFV to once daily Atripla in adult HIV-1 infected subjects with raised cholesterol.
Number of Subjects Planned:	180 randomized subjects (90 per group)
Target Population:	Adult, HIV-1 infected, TDF and FTC-naïve subjects on a stable HAART regimen of Kivexa + EFV, with raised total fasting cholesterol.
Duration of Treatment:	24 weeks
Diagnosis and Main Eligibility Criteria:	• Documented confirmed total cholesterol ≥ 5.2 mmol/L for last two consecutive testings (at least 4 weeks apart) with last result ≤ 4 weeks prior to Screening<br>• Plasma HIV-1 RNA < 50 copies/mL ≥ 12 weeks prior to Screening<br>• Stable HAART regimen of Kivexa + EFV for at least 24 weeks prior to Screening<br>• No previous TDF, FTC or ADV therapy<br>• Concomitant lipid regulating therapy is permitted but must be stable for at least 12 weeks prior to Screening and remain stable throughout the treatment phase of the study
Study Procedures/ Frequency:	The following will be performed at all visits (Screening, Baseline, Weeks 4, 12, 16, and 24):<br>• Hematology, serum chemistry and urinalysis tests<br>• Plasma HIV RNA (viral load)-<br>• CD4/CD8 cell count and percentage<br>• Calculated creatinine clearance<br>• Vital signs, weight and physical examination<br>• Pregnancy tests for women of childbearing potential

	The following will be performed at Baseline, Weeks 4, 12, 16, and 24:<br><br>• Fasting lipid profile measurements. These will include total cholesterol (TC), high density and low density lipoproteins (HDL, LDL), triglycerides (TG) and cholesterol ratios<br><br>• Fasting glucose<br><br>• Acceptability questionnaires and Adherence questionnaires<br><br>Dispensing to be performed at Baseline, Week 4, Week 12, and Week 16.<br><br>Patient Preference of Medicine (POM) questionnaire at Week 4, Week 12, Week 16, and Week 24
**Test Product, Dose, and Mode of Administration:**	Open label Atripla (efavirenz 600 mg/emtricitabine 200 mg/tenofovir DF 300 mg) once daily for oral administration to be taken on an empty stomach
**Reference Therapy, Dose, and Mode of Administration:**	Open label Kivexa (abacavir (as sulfate) 600 mg/lamivudine 300 mg) once daily for oral administration plus Sustiva® (efavirenz 600 mg) once daily for oral administration
**Criteria for Evaluation:**	
Primary Endpoint:	Change from baseline in total fasting cholesterol at Week 12.
Secondary Endpoints	Efficacy:<br><br>• Proportion of subjects with HIV RNA < 50 copies/mL<br><br>• Proportion of subjects with HIV RNA < 400 copies/mL<br><br>• Changes in CD4 and CD8 cell count<br><br>• Safety and Other:<br><br>• Change in total fasting cholesterol at Week 24<br><br>• Changes in fasting LDL, HDL, triglycerides, non-HDL cholesterol and cholesterol ratios

	• Changes in AST and ALT<br>• Changes in the urine protein / urine creatinine ratio<br>• Changes in the 10-year risk for coronary heart disease outcomes<br>• Effect of using a once daily fixed dose tablet on adherence and acceptability
**Statistical Methods:**	Wilcoxon Rank-Sum test will be used to test the hypothesis that switching from Kivexa + EFV to Atripla leads to lower total fasting cholesterol at Week 12 compared to remaining on Kivexa + EFV. Hypothesis testing will be two-sided at 0.05 level.<br><br>In general, point and interval estimation (i.e., two-sided 95% confidence intervals) will be used to summarize the data. Mean standard deviation, minimum, 25th, 50th (median), 75th percentiles, and maximum will summarize the continuous measures. Count and proportion of patients will summarize the categorical and ordinal measures. These statistics provide sufficient information on the distribution of the endpoints. The summary statistics will be presented in tabular and/or graphical format.

This study will be conducted in accordance with the guidelines of Good Clinical Practices (GCPs) including archiving of essential documents.

GLOSSARY OF ABBREVIATIONS AND DEFINITION OF TERMS

° C	degrees Celsius
AE	adverse event
ALT	alanine aminotransferase
AST	aspartate aminotransferase
BID	twice a day (*bis in die*)
BUN	blood urea nitrogen
CBC	complete blood count
CrCl (calc)	calculated creatinine clearance
CRF	case report form(s)
CRO	contract research organization
DNA	deoxyribonucleic acid
DSPH	Drug Safety and Public Health
ECG	electrocardiogram
eCRF	electronic case report form(s)
EEG	electroencephalogram
EMEA	European Medicines Agency
EudraCT	European Clinical Trials Database
FDA	(United States) Food and Drug Administration
FSH	follicle stimulating hormone
GCP	Good Clinical Practice (Guidelines)
GGT	gamma glutamyl transferase
GSI	Gilead Sciences, Inc.
HAART	highly active antiretroviral therapy
Hct	hematocrit
HDL	high-density lipoprotein
HDPE	high-density polyethylene
Hb	hemoglobin
HIV	human immunodeficiency virus
HLGT	high-level group term
HLT	high-level term
IB	investigator’s brochure
ICH	International Conference on Harmonization
ID	identification
IEC	independent ethics committee
IND	Investigational New Drug (Application)
ITT	intent-to-treat (analysis or subset)
IV	intravenous

GLOSSARY OF ABBREVIATIONS AND DEFINITIONS OF TERMS (CONTINUED)

IUD	intrauterine device
KS	Kaposi sarcoma
LDL	low-density lipoprotein
LLN	lower limit of the normal range
LLT	lower-level term
MAA	marketing authorization application
MedDRA	Medical Dictionary for Regulatory Activities
NNRTI	non-nucleoside reverse transcriptase inhibitor
NRTI	nucleoside reverse transcriptase inhibitor
PBMC	peripheral blood mononuclear cell(s)
PE	physical examination
PK	pharmacokinetic
PO	oral administration (*per os*, by mouth)
PT	preferred term
QD	once daily (use only in tables)
RNA	ribonucleic acid
SAE	serious adverse event
SmPC	summary of product characteristics
SOC	system organ class
T	Treated
TC	total cholesterol
TG	triglycerides
TID	three times a day (*tris in die*)
ULN	upper limit of the normal range
WOCBP	women of child bearing potential

1. INTRODUCTION

1.1. Background and Rationale for the Current Study

Approximately 42 million people are infected with the human immunodeficiency virus (HIV) worldwide {10403}. Infection with HIV is characterized by a severe weakening of the immune system leading to the occurrence of opportunistic infections and cancers. Over the past decade, there have been significant advances toward fighting the progression of HIV disease. HIV viral load and CD4 cell count levels are used as surrogate markers to monitor disease progression. Current treatment strategies consist of the utilization of potent combination antiretroviral therapy (often referred to as highly active antiretroviral therapy, or "HAART") to suppress HIV replication below detectable limits thus limiting the potential for resistant viruses, increasing or maintaining CD4 cell counts, and thereby delaying disease progression. However, prolonged therapy may be complicated by adverse effects, including the metabolic syndrome (dyslipidemia, insulin resistance, visceral adiposity) and subcutaneous lipoatrophy {5117}: collectively known as lipodystrophy. Dyslipidemia has emerged as an important issue in HIV infected individuals receiving HAART {10979}, {5675}and data from the DAD Study Group have revealed an increase in risk of myocardial infarction in HIV positive patients receiving antiretroviral therapy {5903}. This prospective cohort study however failed to show an independent association between exposure to Non-nucleoside Reverse Transcriptase Inhibitors (NNRTIs) and myocardial infarction. No analysis within this study was undertaken on the individual or collective contribution of nucleoside reverse transcriptase inhibitors (NRTIs) to dyslipidemia or cardiovascular events. Several studies have been undertaken recently to investigate the metabolic effects of switching from older NRTIs, particularly the thymidine analogues to newer agents. Focus has been on changes in lipid parameters, lipodystrophy assessments and other metabolic parameters. A number of these switch studies have analyzed the effects of changing an NRTI because of toxicity to either abacavir or tenofovir DF and the results suggest that abacavir generally has a dyslipidemic effect with increases in total cholesterol, triglycerides and low density lipoprotein cholesterol (RAVE study, GS-02-1008), {3551}, {10958}; whilst tenofovir DF appears to have a beneficial effect (SWEET study, GS-MC-164-0111), (RAVE study, GS-02-1008), {10619}, {9299}, {10159}. Only one published study has compared switching an NRTI to abacavir versus tenofovir DF (RAVE study, GS-02-1008). This study of 48 weeks duration reported mean reductions in total, LDL and HDL cholesterol and triglycerides in patients randomized to tenofovir DF with increases in these parameters for abacavir treated patients. A recent Kivexa vs Truvada study showed that the differences between the abacavir and tenofovir DF groups were statistically significant for both total cholesterol, HDL and LDL cholesterol (BICOMBO study, IN-ES-164-0132). Both Kivexa and Truvada demonstrated that they are less associated with fat loss / redistribution and may even be able to reverse these changes if patients are switched off older nucleosides to these drugs at an early stage. Truvada has also demonstrated improvements in the lipid profile of patients switching from older nucleoside backbones and this improvement appears to be greater than that of Kivexa.

This study aims to investigate whether patients switching from Kivexa + Efavirenz to Atripla, who already have raised total cholesterol prior to switching, have an improvement in their total cholesterol after 12 weeks of treatment. If an improvement is demonstrated the study aims to show whether this has a beneficial effect on the patient's overall cardiovascular risk and long term prognosis.

1.2. EFV/FTC/TDF Fixed-Dose Combination Tablets (Atripla)

The EFV/FTC/TDF tablet (ATR, Atripla) is a fixed-dose combination of efavirenz (EFV, Sustiva®, Stocrin®), emtricitabine (FTC, Emtriva®), and tenofovir disoproxil fumarate (tenofovir DF, TDF, Viread®) and was approved in the Unites States on 12 July 2006 for the treatment of HIV-1 infection in adults. In the European Union a Marketing Authorization Application (MAA) has been filed with the European Medicines Agency (EMEA) and is undergoing review. The formulation received a positive opinion from the CHMP on 18th October 2007, with a recommended indication for the treatment of HIV-1 infected adults with virologic suppression to HIV-1 RNA levels of < 50 copies/ml on their current antiretroviral combination therapy for more than 3 months. Patients must not have experienced virological failure on any prior antiretroviral therapy and must be known not to have harboured virus strains with mutations conferring significant resistance to any of the three components contained in Atripla prior to initiation of their first antiretroviral treatment regimen. The CHMP Opinion on Atripla is currently being considered by the European Commission which is empowered to grant marketing authorizations in the European Union.

The individual components (efavirenz, 600 mg; emtricitabine, 200 mg; and tenofovir DF, 245 mg of tenofovir disoproxil as fumarate, equivalent to 300 mg of tenofovir DF or 136 mg of tenofovir) and the fixed-dose combination tablet of emtricitabine/tenofovir DF (Truvada®, TVD) are individually approved for marketing for the treatment of human immunodeficiency virus type 1 (HIV-1) infection in the United States, European Union, and other markets worldwide.

Further information is available in the Atripla Investigators Brochure.

1.3. Other Study Drugs

The other drugs being used in the study are: efavirenz (EFV, Sustiva, Stocrin) and fixed combination tablets of abacavir/lamivudine (Kivexa).

Further information regarding these drugs can be found in the respective Summary of Product Characteristics (SmPC).

2. OBJECTIVES

The primary objective of this study is as follows:

- To determine if switching from a stable HAART regimen of Kivexa + EFV to once daily Atripla leads to a reduction in total fasting cholesterol at 12 weeks.

The secondary objectives of this study are:

- Evaluation of fasting metabolic parameters (e.g., LDL, HDL, non-HDL cholesterol, triglycerides, and cholesterol ratios).
- Evaluation of efficacy and safety by assessing adverse events, clinical laboratory tests, physical examinations and vital signs at every visit.
- Evaluation of changes in the 10-year risk factor for coronary heart disease outcomes as measured by total cholesterol, HDL, blood pressure, smoking status, treatment for hypertension, sex and age.

3. STUDY DESIGN

This protocol describes a Phase 4, open label, randomized, UK multi-center, controlled study to assess the effect on lipid profile of switching from a stable HAART regimen of Kivexa + EFV to once daily Atripla in adult HIV-1 infected subjects with raised cholesterol.

3.1. Treatment Plan and Regimen

All Screening and Baseline tests and procedures must be completed prior to the receipt of the first dose of study drug.

At Baseline, subjects will be randomized 1:1 to one of two treatment groups:

Treatment Group 1: switch to Atripla

Treatment Group 2: Continuation of previous stable HAART regimen of Kivexa + EFV

Initiation of treatment with study drugs must take place within 24 hours after Baseline visit. At Week 12, subjects in Treatment Group 2 (continuation of Kivexa and EFV regimen) will switch to Atripla. Treatment in both groups will continue through Week 24 (Figure 3-1).

Figure 3-1. Study Schema

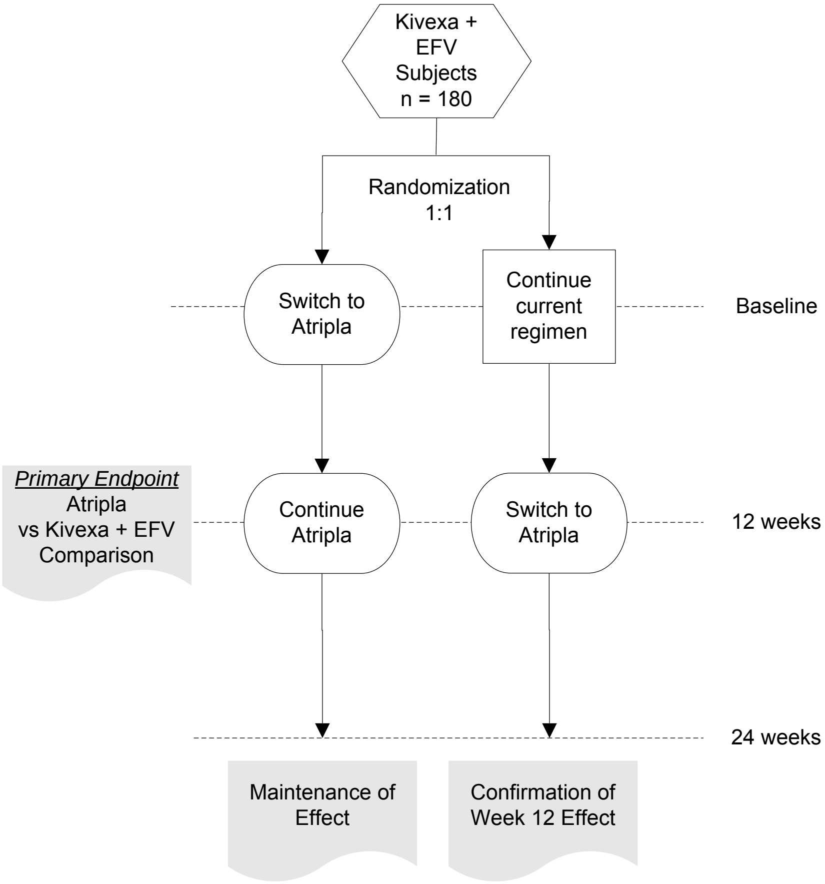

The full visit schedule is detailed in Appendix 2.

4. SUBJECT POPULATION

4.1. Number of Subjects and Subject Selection

A total of 180 subjects with raised cholesterol, taking Kivexa + EFV are planned for enrolment in this study. Subjects will be randomized 1:1 to either remain on current therapy or switch to once daily Atripla. Concomitant lipid regulating therapy is permitted but must be stable for ≥ 12 weeks prior to Screening and must remain stable throughout the treatment phase of the study.

4.2. Inclusion Criteria

- Subjects must meet *all* of the following inclusion criteria to be eligible for participation in this study:
- ≥ 18 years old
- Plasma HIV RNA < 50 copies/mL ≥ 12 weeks prior to Screening
- Stable HAART regimen of Kivexa + EFV for ≥ 24 weeks prior to Screening
- Documented confirmed raised total cholesterol ≥ 5.2 mmol/L for last two consecutive tests (at least 4 weeks apart) with the last result ≤ 4 weeks prior to Screening
- Subject willing to continue current unmodified HAART for 12 weeks if randomized to Group 2
- Subjects requiring concomitant lipid regulating therapy must be established on a stable dose/frequency ≥ 12 weeks prior to Screening and be expected to remain stable in dose and frequency throughout the treatment phase of the study
- Adequate renal function by calculated creatinine clearance ≥ 60 mL/min according to the Cockcroft Gault formula
- Negative serum pregnancy test (females of childbearing potential only i.e., not surgically sterile or at least 2 years post-menopausal)
- Hepatic Total Bilirubin ≤ 1.5 mg/dL
- Adequate haematologic function of absolute neutrophil count ≥ 1000/mm^3, platelets ≥ 25,000/mm^3, Haemoglobin ≥ 8.0g/dL

- Women of childbearing potential (WOCBP) must be using two methods of contraception to avoid pregnancy throughout the study and for up to 12 weeks after the last dose of study drugs in such a manner that the risk of pregnancy is minimized. Subjects may choose two (barrier plus highly effective method – see section 7.8 for further discussion) of the birth control methods listed below:
  - Hormonal birth control drugs
  - Male or female condoms with or without spermicidal gels
  - Diaphragm cervical cap with or without spermicidal gels
  - Intrauterine device
  - Female subjects who utilize hormone contraceptive as one of their birth control methods must have used the same methods for at least 3 months prior to study dosing
- Female subjects who are postmenopausal for less than 2 years are required to have FSH ≥ 40 mIU/mL. If the FSH is < 40 mIU/mL, the subject must agree to use highly effective method of birth control (as described above) to participate in the study
- Male subjects who are sexually active must be willing to use effective barrier contraception (e.g. condom with spermicide) during heterosexual intercourse from screening through completion of the study and continuing for up to 12 weeks after the last dose of study drugs
- Life expectancy ≥ 1 year
- The ability to understand and sign a written informed consent form, which must be obtained prior to initiation of study procedures

4.3. Exclusion Criteria

Subjects who meet *any* of the following exclusion criteria are not to be enrolled in this study:

- Pregnant or lactating subjects
- Previous treatment with emtricitabine (FTC), tenofovir DF (TDF) or adefovir dipivoxil (ADV)
- Known hypersensitivity to emtricitabine (FTC), tenofovir DF (TDF), efavirenz (EFV) or Truvada
- Documented resistance to any of the study drugs (either genotypic or phenotypic)
- Severe hepatic impairment

- Hepatic transaminases (AST and ALT) ≥ 5 × upper limit of normal (ULN)
- Subjects receiving ongoing therapy with any of the medications that are contraindicated with any of the study drugs. Administration of any of these medications must be discontinued at least 30 days prior to the Baseline visit and for the duration of the study period. The full list of disallowed medications can be found in appendix 7.
- Active, serious infections (other than HIV infection) requiring parenteral antibiotic therapy within 15 days prior to screening
- Prior history of significant renal or bone disease
- Any current known clinical or symptomatic laboratory parameter of GSI grade 4 (see appendix 4). Asymptomatic grade 4 abnormalities will be permitted at the discretion of the Investigator if deemed clinically appropriate (excluding AEs and laboratory parameters mentioned elsewhere in the inclusion/exclusion criteria). Abnormalities deemed insignificant by the Investigator must be discussed with the Sponsor prior to enrollment.
- Malignancy other than cutaneous Kaposi sarcoma (KS) or basal cell carcinoma. Subjects with biopsy-confirmed cutaneous KS are eligible, but must not have received any systemic therapy for KS within 30 days of baseline and are not anticipated to require systemic therapy during the study
- Current alcohol or substance use judged by the investigator to potentially interfere with subject study compliance
- Subjects currently taking part in any other clinical trial using an investigational product, with the exception of studies where the treatment studied has been stopped for more than 1 month
- Any other clinical condition or prior therapy that, in the opinion of the investigator, would make the subject unsuitable for the study or unable to comply with the dosing requirements

5. STUDY DRUGS

5.1. Randomization and Blinding

A centralized randomization procedure will be used for this study whereby treatment will be assigned to subjects at each investigational site via use of a randomization fax to a member of Gilead staff which will be completed according to the randomization code and faxed back to the investigational site.

Gilead will supply Atripla tablets to each site. Kivexa + EFV tablets will be provided by each site according to normal local procedure (not supplied by Gilead).

Study drugs will be dispensed at Baseline, Week 4, Week 12 and Week 16. Subjects will be instructed to return unused study medication in the original container at each study visit starting with the Week 4 visit. The investigator will be responsible for maintaining accurate records for all study drug dispensed and returned (including Kivexa and Efavirenz tablets) and the inventory must be available for inspection by the study monitor. For subjects randomized to continue their current treatment, any Kivexa or Efavirenz tablets remaining from their previous prescription should be counted and documented on the accountability log as part of their dispensed study medication.

All study medication supplies, including partially used or empty bottles, and the dispensing logs, must be accounted for by the study monitor prior to destruction or return.

5.2. Atripla Tablets

5.2.1. Formulation

Atripla tablets are pink, modified capsule-shaped, film-coated tablets containing 600 mg of efavirenz, 200 mg of emtricitabine, and 300 mg of tenofovir DF, debossed with ‘123’ on one side, plain on the other side. Each tablet core contains the following inactive ingredients: croscarmellose sodium, hydroxypropyl cellulose, magnesium stearate, microcrystalline cellulose, and sodium lauryl sulfate. The efavirenz/emtricitabine/tenofovir DF combination tablets are film-coated with a film-coating material containing black iron oxide, polyethylene glycol, polyvinyl alcohol, red iron oxide, titanium dioxide, and talc.

5.2.2. Packaging and Labeling

Atripla tablets are packaged in white, child-resistant, HDPE bottles containing 30 tablets each and a silica gel desiccant canister to protect the product from humidity. To ensure stability of the tablets and proper product identification, the drug must be dispensed in the HDPE container, in which it is supplied.

Each bottle of Atripla tablets will be labeled in accordance with GMP Annex 13 for Clinical Trial medication.

5.2.3. Storage and Handling

Store in the original container and keep the container tightly closed in order to protect from moisture. No special storage conditions required.

5.2.4. Dosage and Administration

Subjects randomized to Group 1 will be instructed to take one Atripla tablet, once-daily. It is recommended that Atripla tablets be swallowed whole with water on an empty stomach. Food may increase Efavirenz exposure and may lead to an increase in the frequency of adverse events. In order to improve the tolerability of nervous system undesirable effects related to Efavirenz, bedtime dosing is also recommended.

5.3. Kivexa (ABC/3TC Tablets)

5.3.1. Formulation

Kivexa tablets are orange, film-coated, modified capsule shaped tablets, debossed with GS FC2 on one side. Each tablet contains 600 mg of abacavir (as sulfate) and 300 mg lamivudine.

Each tablet core contains the inactive ingredients: magnesium stearate, microcrystalline cellulose and sodium starch glycollate. The Kivexa tablets are film-coated with Opadry Orange YS-1-13065-A containing: hypromellose, titanium dioxide (E171), macrogol 400 and polysorbate 80.

5.3.2. Packaging and Labeling

Kivexa tablets are packaged in opaque white (PVC/PVDC/Aluminum) blister packs and white (HDPE) bottles with child-resistant closure; 30 tablets per pack. Kivexa tablets will be provided by each site and labeled according to normal local procedure.

5.3.3. Storage and Handling

Do not store above 30°C.

5.3.4. Dosage and Administration

Subjects randomized to Group 2 will be instructed to continue taking their daily dose of Kivexa (abacavir (as sulfate) 600 mg/lamivudine 300 mg) once daily for oral administration plus Sustiva (efavirenz 600 mg) once daily for oral administration.

5.3.5. Further Information

Further information regarding Kivexa tablets can be found in the Summary of Product Characteristics (SmPC).

5.4. Efavirenz Tablets (Sustiva®)

5.4.1. Formulation

Efavirenz tablets (Sustiva) are dark yellow, film-coated, capsule shaped tablets, printed with "SUSTIVA" on both sides. Each tablet contains 600 mg of efavirenz and the following inactive ingredients: croscarmellose sodium, microcrystalline cellulose, sodium laurilsulfate, hydroxypropylcellulose, lactose monohydrate and magnesium stearate. The film coating contains: hypromellose (E464), titanium dioxide (E171), macrogol 400, yellow iron oxide (E172) and carnauba wax. The printing ink contains: hypromellose (E464), propylene glycol, cochineal carminic acid (E120), indigo carmine (E132) and titanium dioxide (E171).

5.4.2. Packaging and Labeling

Efavirenz tablets are packaged in HDPE bottles with a child-resistant polypropylene closure or blister packs, each containing 30 film-coated tablets. EFV tablets will be provided by each site and labeled according to normal local procedure.

5.4.3. Storage and Handling

This medicinal product does not require any special storage conditions.

5.4.4. Dosage and Administration

The recommended dosage of Efavirenz in combination with NRTIs is 600 mg orally, once daily. Subjects randomized to Group 2 will be instructed to continue taking their daily dose of Kivexa + EFV.

5.4.5. Further Information

Further information regarding Efavirenz tablets can be found in the Summary of Product Characteristics (SmPC).

5.5. Prior and Concomitant Medications

Subjects will continue their concomitant medications as prescribed.

Should subjects need to start treatment with any excluded concomitant medication the sponsor must be consulted prior to initiation of the new medication. In instances where an excluded medication is initiated prior to discussion with the sponsor, the investigator must notify the sponsor as soon as they are aware of the use of the excluded medication. In such circumstances the subject will need to be excluded from the study.

6. STUDY PROCEDURES

The study procedures to be conducted for each subject enrolled in the study are presented in tabular form in Appendix 2 and described in the text that follows. Additional information on the study procedures is provided in the study procedures manual.

Any deviation from protocol procedures should be notified to the sponsor or Contract Research Organization (CRO).

All blood and urine specimens will be sent to a central laboratory for analysis. The urine pregnancy test will be performed at site using a urine dipstick method.

6.1. Subject Enrollment and Treatment Assignment

It is the responsibility of the investigator to ensure that each subject is eligible for the study before enrolment. Once a subject number has been assigned to a subject, it will not be reassigned to any other subject.

6.2. Pretreatment Assessments

6.2.1. Screening Visit

Subjects will be screened to determine eligibility for participation in the study. The following will be performed and documented at screening:

- Written informed consent
- Medical and medication history including history of HIV disease-related events and prior HIV and lipid lowering medications
- Concomitant medications (including lipid lowering agents) and ongoing antiretroviral medications
- Laboratory confirmation of HIV infection (only to be performed in cases where documentation of such is unavailable)
- Hepatitis B and Hepatitis C testing
- Serum pregnancy test (females of childbearing potential only)
- Serum FSH (for women who are postmenopausal for less than 2 years only)
- Physical examination including vital signs, body weight, and height
- CD4/CD8 cell count and percentage

- Hematology profile: complete blood count (CBC) with differential and platelet count
- Chemistry profile: albumin, AST, ALT, direct bilirubin, total bilirubin, creatinine, potassium, phosphate, glucose, calcium and amylase, CrCl (calc), sodium, bicarbonate, chloride
- Urinalysis–proteinuria, glycosuria, pH, protein/creatinine ratio
- Plasma HIV RNA (viral load)

Subjects meeting all of the inclusion criteria and none of the exclusion criteria will return to the clinic within 4 weeks of Screening for randomization into the study.

6.2.2. Baseline Assessments

For this trial, Baseline (Day 0) is defined as the day on which the subject's first dose of the study medication is dispensed. The subject should be instructed to arrive for the assessments not having had any food for the preceding 10 hours.

The subject must complete all study procedures before administering the study drug:

- Concomitant medications (including lipid lowering agents) and ongoing antiretroviral medications
- Adverse events
- Serum pregnancy test (females of childbearing potential only)
- Physical examination including vital signs and body weight
- CD4/CD8 cell count and percentage
- Hematology profile: complete blood count (CBC) with differential and platelet count
- Chemistry profile: Albumin, AST, ALT, direct bilirubin, total bilirubin, creatinine, potassium, phosphate, glucose, calcium and amylase, CrCl (calc), sodium, bicarbonate, chloride
- Urinalysis–proteinurea, glycosuria, pH, protein/creatinine ratio
- Plasma HIV RNA
- Fasting glucose
- Fasting lipid profile – TC, LDL, HDL, non-HDL, TG, cholesterol ratios

- Acceptability questionnaires:
    - Treatment Satisfaction and Symptoms Questionnaire, Appendix 6
    - HAART Intrusiveness Scale (m-HIS), Appendix 8
- Adherence questionnaire (Perceived Ease of Adherence (PERC) and Visual Analogue Scale (VAS), Appendix 9)

Upon completion of Baseline assessments, the investigator should dispense assigned study drug to the subject. The subject should be instructed to initiate study drugs within 24 hours of the Baseline visit.

6.3. Treatment Assessments

The following evaluations are to be completed during visits at Week 4, 12, 16, and 24. All study visits should be completed within ± 5 days based on the Baseline visit.

- Concomitant medications (including lipid lowering agents) and ongoing antiretroviral medications
- Adverse events
- Physical examination including vital signs and body weight
- Urine pregnancy test (females of childbearing potential only); positive urine pregnancy tests will be confirmed with a serum test
- CD4/CD8 cell count and percentage
- Hematology profile: complete blood count (CBC) with differential and platelet count.
- Chemistry profile: Albumin, AST, ALT, direct bilirubin, total bilirubin, creatinine, potassium, phosphate, glucose, calcium and amylase, CrCl (calc), sodium, bicarbonate, chloride
- Urinalysis–proteinuria, glycosuria, pH, protein/creatinine ratio
- Plasma HIV RNA
- Fasting glucose
- Fasting lipid profile–TC, LDL, HDL, non-HDL, TG, cholesterol ratios
- Acceptability questionnaires:

- Treatment Satisfaction and Symptoms Questionnaire, Appendix 6
- HAART Intrusiveness Scale (m-HIS), Appendix 8

- Adherence questionnaires:
  - Patient Preference of Medicine (POM), Appendix 5 (Week 4 and Week 16 for switched subjects only)
  - Perceived Ease of Adherence (PERC) and Visual Analogue Scale (VAS), Appendix 9
- Pill count

Study drug to be dispensed at Week 4, Week 12 and Week 16 only. No study drug will be dispensed at the Week 24 visit.

6.4. Unscheduled Visit

The investigator can bring the subject back for an additional unscheduled visit if it is felt necessary to follow up on abnormal laboratory tests or adverse events. In particular, if a subject has viral load ≥ 400 copies/mL the subject should have a second test performed within 2−4 weeks.

6.5. 30 Day Follow-up Assessment

The investigator will contact the subject to conduct an Outcome Visit thirty days after completion or discontinuation from the study to assess updates on Adverse Events, concomitant medications and antiretroviral medications and to monitor any pregnancies that may have occurred during this period. If study medication was not returned at the last visit the subject must return to the clinic for the outcome visit and return all unused study medication (and used packaging). Subjects that do not have any study medication can be contacted by telephone in order to complete the outcome form. This telephone contact will still be considered a formal study visit.

Last Patient Last Visit (LPLV) is defined as the last protocol required follow-up visit completed by a subject enrolled in this study.

6.6. Assessments for Premature Discontinuation from Study

If a subject discontinues study medication dosing (for example, as a result of an adverse event [AE]), every attempt should be made to keep the subject in the study and continue to perform the required study-related follow-up and procedures (see Section 6.7). If this is not possible or acceptable to the subject or investigator, the subject may be withdrawn from the study.

6.7. Criteria for Discontinuation of Study Treatment

Study medication may be discontinued in the following instances:

- Intercurrent illness that would, in the judgment of the investigator, affect assessments of clinical status to a significant degree
- Unacceptable toxicity, as defined in the toxicity management section of the protocol, or toxicity that, in the judgment of the investigator, compromises the ability to continue study-specific procedures or is considered to not be in the subject's best interest
- Virological failure defined as two consecutive viral load values ≥ 400 copies/mL taken within 2–4 weeks
- Subject request to discontinue for any reason
- Subject noncompliance
- Pregnancy during the study
- Discontinuation of the study at the request of Gilead, Regulatory Agency or an IEC
- Subject develops renal impairment (CrCl < 50 mL/min)

7. ADVERSE EVENTS AND TOXICITY MANAGEMENT

7.1. Adverse Events

An adverse event (AE) is any untoward medical occurrence in a clinical investigation subject administered a medicinal product and which does not necessarily have a causal relationship with this treatment. An AE can therefore be any unfavorable and unintended sign, symptom, or disease temporally associated with the use of a medicinal product, whether or not considered related to the medicinal product.

AEs may also include the following:

- Pre- or post-treatment complications that occur as a result of protocol mandated procedure(s) (e.g., invasive procedures such as venipuncture, biopsy) during or after screening (before the administration of study drug)
- Any pre-existing condition that increases in severity, or changes in nature during or as a consequence of the study drug phase of a human clinical trial

All AEs that occur after the screening visit and throughout the duration of the study, including the follow-up off-study medication period should be recorded as an AE.

An AE does not include the following:

- Medical or surgical procedures (e.g., surgery, endoscopy, tooth extraction, transfusion) performed; the condition that leads to the procedure is an adverse event
- Pre-existing diseases or conditions or laboratory abnormalities present or detected before the screening visit that do not worsen
- Situations where an untoward medical occurrence has not occurred (e.g., hospitalization for elective surgery, social and/or convenience admissions)
- Overdose of either non-Gilead drug(s) or concomitant medication without any signs or symptoms, unless the subject is hospitalized for observation

Any medical condition or clinically significant laboratory abnormality with an onset date before the screening visit and not related to a protocol-associated procedure is not an AE. It is considered to be pre-existing and should be documented on the medical history eCRF page.

In addition, pregnancy itself is not considered an AE. However, any pregnancy complication or elective termination of a pregnancy for medical reasons will be recorded as an AE or an SAE.

7.2. Assessment of Adverse Events

All AEs will be assessed by the investigator and recorded on the AE CRF page. The AE entry should indicate whether or not the AE was serious, the start date (AE onset), the stop date (date of AE resolution), whether or not the AE was related to study drug or to a study procedure, the action taken with study drug due to the AE, and the severity of the AE.

Severity should be recorded and graded according to the Table for Grading of Clinical Adverse Events and Laboratory Abnormalities (see Appendix 4). See Section 7.6 for AEs associated with laboratory abnormalities.

The relationship to study drug therapy should be assessed using clinical judgment and the following definitions:

- **No**: Evidence exists that the adverse event has an etiology other than the study drug. For SAEs, an alternative causality must be provided (e.g., pre-existing condition, underlying disease, intercurrent illness, or concomitant medication).

- **Yes**: A temporal relationship exists between the AE onset and administration of the study drug that cannot be readily explained by the subject’s clinical state or concomitant therapies. Furthermore, the AE appears with some degree of certainty to be related, based on the known therapeutic and pharmacologic actions or adverse event profile of the study drug. In case of cessation or reduction of the dose, the AE abates or resolves and reappears upon rechallenge.

It should be emphasized that ineffective treatment should not be considered as causally related in the context of adverse event reporting.

The relationship to study procedures (e.g., invasive procedures such as venipuncture or biopsy) should be assessed using the following definitions:

- **No:** Evidence exists that the adverse event has an etiology other than the study procedure.

- **Yes:** The adverse event occurred as a result of protocol-mandated procedures such as venipuncture or biopsy.

7.3. Serious Adverse Events

A **serious adverse event** (SAE) is defined as follows:

Any adverse drug experience occurring at any dose that results in any of the following outcomes:

- Death
- Life-threatening situation (subject is at **immediate** risk of death)
- In-patient hospitalization or prolongation of existing hospitalization (excluding those for study therapy or placement of an indwelling catheter, unless associated with other SAEs)
- Persistent or significant disability/incapacity
- Congenital anomaly/birth defect in the offspring of a subject who received study drug
- Other: medically significant events that may not be immediately life-threatening or result in death or hospitalization, but based upon appropriate medical and scientific judgment, may jeopardize the subject or may require medical or surgical intervention to prevent one of the outcomes listed above

Examples of such events are as follows:

— Intensive treatment in an emergency room or at home for allergic bronchospasm

— Blood dyscrasias or convulsions that do not result in hospitalization

— Development of drug dependency or drug abuse

Clarification of Serious Adverse Events

- Death is an outcome of an AE, and not an adverse event in itself. In reports of death due to "Disease Progression," where no other information is provided, the death will be assumed to have resulted from progression of the disease being treated with the study drug(s).
- All deaths, regardless of cause or relationship, must be reported for subjects on study and for deaths occurring within 30 days of last study drug dose or within 30 days of last study evaluation, whichever is longer.
- The subject may not have been on study drug at the time of the event. Dosing may have been given as treatment cycles or interrupted temporarily before the onset of the SAE, but may have contributed to the event.
- "Life-threatening" means that the subject was at immediate risk of death from the event as it occurred. This does not include an event that might have led to death if it had occurred with greater severity.
- Complications that occur during hospitalizations are AEs. If a complication prolongs the hospitalization, it is a SAE.

- "In-patient hospitalization" means the subject has been formally admitted to a hospital for medical reasons, for any length of time. This may or may not be overnight. It does not include presentation and care within an emergency department.

- The investigator should attempt to establish a diagnosis of the event on the basis of signs, symptoms and/or other clinical information. In such cases, the diagnosis should be documented as the AE and/or SAE and not the individual signs/symptoms.

A distinction should be drawn between seriousness and severity of AEs. An AE that is assessed as Grade 4 (potentially life-threatening) should not be confused with an SAE. Severity is a category utilized for rating the intensity of an event; and both AEs and SAEs can be assessed as Grade 4. An event is defined as "serious" when it meets one of the predefined outcomes described above in Section 7.3.

7.4. Overdose

Any accidental or intentional overdose (any increase in frequency or dosage of study medication that exceeds what is mandated by the protocol), misuse or abuse of study medication as well as any Gilead product taken as a concomitant medication, whether suspected or confirmed, and whether or not associated with an adverse experience, must be reported using the SAE form and forwarded within 24 hours to the Gilead Drug Safety & Public Health. The procedure for reporting is the same as an SAE, as outlined in Section 7.5. Although the procedure for reporting all overdoses will be the same as that for reporting SAEs, an overdose will be considered an SAE only if any of the seriousness criteria are met. Any clinical sequelae in association with the overdose should be reported as an AE (as outlined in Section 7.1) or SAE (as outlined in Section 7.3). Details of signs or symptoms, clinical management and outcome should be reported if available.

7.5. Serious Adverse Event Reporting Requirements

7.5.1. All Serious Adverse Events

Gilead has requirements for expedited reporting to worldwide regulatory authorities of SAEs meeting specific requirements; therefore, Gilead must be notified immediately regarding the occurrence of any SAE that occurs after the screening visit, including SAEs resulting from protocol-associated procedures performed from screening onwards. The procedures for reporting all SAEs, regardless of causal relationship, are as follows:

- Record the SAE on the AE eCRF page and complete the "Serious Adverse Event Report" form

- Fax the SAE form to the attention of the Gilead DSPH Department within 24 hours of the investigator's knowledge of the event. Contact information is as follows:

Gilead DSPH Representative:	Name:	Rie Devert, RN MSN
	Title:	Associate Manager, DSPH
	Phone:	01223 897500
	Fax:	01223 897290
	Email:	csafety@gilead.com
Gilead Medical Monitor:	Name:	Dr Cham Herath
	Phone:	+44 (0) 1223 897300
	Fax:	+44 (0) 1223 897281
	E-mail:	cham.herath@gilead.com

- For fatal or life-threatening events, also fax copies of hospital case reports, autopsy reports, and other documents when requested and applicable. **Any subject data sent to Gilead must be anonymised (whilst remaining traceable by the sender) before sending.**

Gilead may request additional information from the investigator to ensure the timely completion of accurate safety reports.

The investigator must take all therapeutic measures necessary for resolution of the SAE. Any medications necessary for treatment of the SAE must be recorded onto the concomitant medication section of the subject's eCRF.

Follow-up of adverse events will continue through the last day on study (including the follow-up off-study medication period of the study) and/or until the investigator and/or Gilead determine that the subject's condition is stable. Gilead may request that certain adverse events be followed until resolution.

7.5.2. Investigator and Sponsor Reporting Requirements for SAEs

An SAE may qualify for reporting to regulatory authorities if the SAE is related to the study drug. Expectedness of SAEs will be determined by Gilead using reference safety information specified in the Investigator's Brochure. All investigators will receive a formal notification describing unexpected related serious adverse events. In accordance with the EU Clinical Trials Directive (2001/20/EC), Gilead will notify the co-ordinating Ethics Committees in concerned Member States of serious adverse events that are unexpected and possibly attributable to the study drug.

7.5.3. Poststudy Reporting Requirements

All deaths, regardless of cause or relationship, must be reported for subjects on study and for all deaths occurring within 30 days of last study drug dose or within 30 days of last study evaluation, whichever is longer.

Investigators are not obligated to actively seek out SAEs beyond the follow-up period for subjects. However, if the investigator learns of an AE or SAE occurring after the

completion/termination visit and the event is deemed by the investigator to be probably or possible related to the use of study drugs, he/she should promptly document and report the event to Gilead Sciences.

7.6. Clinical Laboratory Abnormalities and Other Abnormal Assessments as Adverse Events or Serious Adverse Events

Laboratory abnormalities are usually not recorded as AEs or SAEs. However, laboratory abnormalities (e.g., clinical chemistry, hematology, urinalysis) independent of the underlying medical condition that require medical or surgical intervention or lead to study drug interruption or discontinuation must be recorded as an AE, as well as an SAE, if applicable. In addition, laboratory or other abnormal assessments (e.g., electrocardiogram, X-rays, vital signs) that are associated with signs and/or symptoms must be recorded as an AE or SAE if they meet the definition of an AE (or SAE) as described in Sections 7.1 and 7.3 If the laboratory abnormality is part of a syndrome, record the syndrome or diagnosis.

Severity should be recorded and graded according to the Table for Grading of Clinical Adverse Events and Laboratory Abnormalities (Appendix 4). For adverse events associated with laboratory abnormalities, the event should be graded on the basis of the clinical severity in the context of the underlying conditions; this may or may not be in agreement with the grading of the laboratory abnormality.

7.7. Toxicity Management

- All clinical and clinically significant laboratory toxicities will be managed according to uniform guidelines detailed in Appendix 4.
- Clinical events and clinically significant laboratory abnormalities will be graded according to the Table for Grading of Clinical Adverse Events and Laboratory Abnormalities (Appendix 4).
- Grade 3 and 4 clinically significant laboratory abnormalities should be confirmed by repeat testing within 3 calendar days of receipt of results and before study drug discontinuation, unless such a delay is not consistent with good medical practice.
- Abnormal lipids should be managed in accordance with the NCEP guidelines for intervention (Appendix 10).
- When restarting study drug following resolution of the adverse event, the study drug should be restarted at full dose or modified dose that is dependent upon discussion with the Gilead medical monitor.
- Any recurrence of the study drug-related Grade 3 or 4 clinical or clinically significant laboratory adverse event following dose interruption mandates permanent discontinuation of study drug.

- Any questions regarding toxicity management should be directed to the Gilead medical monitor.

7.7.1. Grades 1 and 2 Laboratory Abnormality or Clinical Event

- Continue study drug at the discretion of the investigator.

7.7.2. Grade 3 Laboratory Abnormality or Clinical Event

- For Grade 3 clinically significant laboratory abnormality or clinical event, study drug may be continued if the event is considered to be unrelated to study drug.

- For a Grade 3 clinical event, or clinically significant laboratory abnormality confirmed by repeat testing, that is considered to be related to study drug, study drug should be withheld until the toxicity returns to ≤ Grade 2.

- If a laboratory abnormality recurs to ≥ Grade 3 following rechallenge with study drug and is considered related to study drug, then study drug should be permanently discontinued and the subject managed according to local practice. Recurrence of laboratory abnormalities considered unrelated to study drug may not require permanent discontinuation.

7.7.3. Grade 4 Laboratory Abnormality or Clinical Event

- For a Grade 4 clinical event or clinically significant Grade 4 laboratory abnormality confirmed by repeat testing that is considered related to study drug, study drug should be permanently discontinued and the subject managed according to local practice. The subject should be followed as clinically indicated until the laboratory abnormality returns to baseline or is otherwise explained, whichever occurs first. A clinically significant Grade 4 laboratory abnormality that is not confirmed by repeat testing should be managed according to the algorithm for the new toxicity grade.

Study drug may be continued without dose interruption for a clinically non-significant Grade 4 laboratory abnormality (e.g., Grade 4 CK after strenuous exercise, or triglyceride elevation that is non-fasting or that can be medically managed) or a clinical event considered unrelated to study drug.

7.8. Risks for Women of Childbearing Potential or During Pregnancy

There are no adequate and well-controlled studies of Atripla or its components in pregnant women. In post-marketing experience through an anti-retroviral pregnancy registry, prospective data have not indicated an association between efavirenz, emtricitabine or tenofovir DF and a specific malformation pattern. More than 200 pregnancies with first trimester exposure to efavirenz as part of a combination antiretroviral regimen have been reported with no specific malformation pattern. Retrospectively, in this registry, a small number of cases of neural tube defects associated with efavirenz exposure, including meningomyelocoele, have been reported but causality has not been established.

Atripla should be used during pregnancy only if the potential benefit outweighs the risk to the fetus, such as in pregnant women without other therapeutic options. Women of childbearing potential should undergo pregnancy testing before initiation of Atripla. Barrier contraception should always be used in combination with other methods of contraception (e.g. oral or other hormonal contraceptives). Because the potential interaction of efavirenz with oral contraceptives has not been fully characterized, a reliable method of barrier contraception should be used in addition to oral contraceptives.

Therefore males and females of child bearing potential (i.e., less than 2 years postmenopausal) must agree to utilize highly effective methods of contraception throughout the study period and for up to 12 weeks following discontinuation of study drug. The investigator should counsel subjects on the most effective method(s) for avoiding pregnancy during the trial. A highly effective method of birth control is defined as a method that results in a low failure rate (i.e., less than 1% per year) when used consistently and correctly, such as implants, injectables, combined oral contraceptives, some IUDs, sexual abstinence, or a vasectomized partner. Females of child bearing potential must have a negative serum pregnancy test at screening. Female subjects who are post menopausal for less than 2 years are required to have an FSH ≥ 40 mIU/mL. If FSH less than 40 mIU/mL, then subjects must agree to use highly effective methods of contraception.

It is not known whether efavirenz, emtricitabine or tenofovir are excreted in human milk. Because of the potential for both HIV transmission and the potential for serious undesirable effects in breast-feeding infants, mothers should be instructed not to breast feed if they are receiving Atripla.

Male subjects who are sexually active must be willing to use effective barrier contraception (e.g., condom or spermicide) during heterosexual intercourse from screening through completion of the study and continuing for up to 12 weeks after discontinuation of study drug.

Use of condoms should be encouraged for all participants with other methods of contraceptives because they have been proven to decrease the risk of sexually transmitted diseases, such as HIV transmission. Female subjects who utilize hormonal contraceptives as one of their birth control methods must have used the same method for at least 3 months

before study dosing.

The subject must be instructed to discontinue all study drugs and inform the investigator **immediately** if she becomes pregnant during the study.

The investigator should report all pregnancies that occur in female subjects as well as female partners of male subjects to Gilead DSPH within 24 hours of becoming aware of the pregnancy, and up to 30 days after the last dose of study drugs. The investigator should counsel the subject regarding the possible effects of prior study drug exposure on the fetus and the need to inform the study site of the outcome of the pregnancy.

The pregnancy itself is not considered an AE. However, any pregnancy complication or elective termination of a pregnancy for medical reasons will be recorded as an AE or an SAE.

A spontaneous abortion is always considered to be an SAE and will be reported as described in the Adverse and Serious Adverse Events section. Furthermore, any SAE occurring as an adverse pregnancy outcome post-study must be reported to Gilead.

All pregnancies that occur during the study should be reported using the Pregnancy Report eCRF page and Clinical Pregnancy Report, and faxed to Gilead DSPH. Monitoring of the subject should continue until the conclusion of the pregnancy. The outcome should be reported to Gilead DSPH using the Pregnancy Outcome Report and faxed to Gilead DSPH. If the end of the pregnancy occurs after the study has been completed, the outcome should be reported directly to Gilead DSPH.

8. STATISTICAL CONSIDERATIONS

This section will serve as the general foundation of the analysis method to be used for the study. Further details will be provided separately in the statistical analysis plan document.

8.1. Analysis Objectives

The primary objective of this study is to investigate the metabolic benefits (in terms of total cholesterol) of switching subjects who are stable on a regimen containing Kivexa combined with efavirenz (EFV) but have high cholesterol levels, to Atripla. Thus, the null hypotheses of no benefit (cholesterol change from baseline in the Atripla group = cholesterol change from baseline in the Kivexa + EFV group) will be tested against the alternative hypothesis that switching to Atripla will have an impact on total cholesterol (cholesterol change from baseline in the Atripla group ≠ cholesterol change from baseline in the Kivexa + EFV group).

8.2. Primary Endpoint

The primary efficacy endpoint is change from baseline in total cholesterol at Week 12.

8.3. Secondary Endpoint

Secondary efficacy endpoints include:

- Proportion of subjects with HIV RNA < 50 copies/mL
- Proportion of subjects with HIV RNA < 400 copies/mL
- Changes in CD4 and CD8 cell count

Secondary safety and other endpoints include:

- Change in total fasting cholesterol at Week 24
- Changes in fasting LDL, HDL, triglycerides non-HDL and cholesterol ratios
- Changes in AST and ALT
- Changes in the urine protein / urine creatinine ratio
- Changes in the 10-year risk for coronary heart disease outcomes
- Effect of using a once daily fixed dose tablet on adherence and acceptability

8.4. Methods of Analysis

8.4.1. Analysis Sets

All Randomized

All randomized set will include all subjects who are randomized into the study.

Treated (T):

All treated set includes all subjects who are randomized and have received at least one dose of the randomly allocated study regimen.

T is the primary analysis set for secondary safety endpoints.

Intent to Treat (ITT)

ITT will include subjects in the T set with at least one post-switch observation and with no major violations of entrance criteria. ITT is the primary analysis set for the analyses of the efficacy endpoints and major deviation for ITT set is considered as HIV RNA > 1000 copies/mL at screening visit.

Modified Intent to Treat (MITT)

MITT will include subjects in the T set with at least one post-switch observation and with no major violations of entrance criteria. MITT is the primary analysis set for the analysis of the primary endpoint (change in total cholesterol) and major deviation for MITT set is considered as total cholesterol < 4.2 mmol/L at the screening visit.

8.4.2. Data Handling Conventions

Conventions of Handling missing data are as follows:

Total cholesterol at Week 12 (primary endpoint): For missing cholesterol at Week 12, the missing results at Week 12 will be imputed by the last observed post-baseline (prior to Week 12) result.

Total cholesterol at Week 24: For subjects receiving Atripla who have missing cholesterol at Week 24 the missing results will be imputed by last observed post-baseline value. For subjects receiving KVX+EFV who have missing cholesterol at study Week 24 the missing result will be imputed by last observed post-switch (post-Week 12) result.

Other lipid parameters: Analyses of other lipid parameters follow the same rules as total cholesterol.

Viral load: For analyses of efficacy endpoints, early discontinuation will be considered as failure.

Missing data will be excluded from the analyses of other endpoints.

8.4.3. Interim Analysis

The primary analysis of the primary endpoint is the change from baseline in total cholesterol at Week 12. Thus, there are no interim analyses planned for this study.

8.5. Demographic Data and Baseline Characteristics

Demographic and baseline characteristics measurements will be summarized for the T analysis set using standard descriptive methods (*overall and by treatment group*).

8.6. Analysis Methods

In general mean, standard deviation, minimum, 25th percentile (Q1), median, 75% percentile (Q3) and maximum will summarize the continuous measures. Count and percent of subjects will summarize the categorical and ordinal measures.

8.6.1. Analyses of Primary and Efficacy Endpoints

Primary Endpoint

The hypothesis of treatment differences between the two groups in cholesterol change from baseline (primary endpoint) will be tested using Wilcoxon Rank-Sum test. This analysis will use the MITT analysis set and handling of missing results will follow the conventions specified in Section 8.4.2. Although nonparametric methods will be utilized for hypothesis testing, 95% confidence intervals for the differences in means of the two groups will be constructed.

A secondary analysis of the primary endpoint based on observed data (no imputation for missing) will be conducted.

Efficacy Endpoints

Count and % of subjects with HIV RNA less than specified thresholds (50, 400 copies/mL), in the ITT analysis set, will summarize the viral load endpoints. Confidence Intervals and/or exact tests may be utilized to compare the success (or failure) rates of the two treatment groups. These analyses will be considered as exploratory since the study is not designed or powered for efficacy endpoints.

8.6.2. Analysis of Secondary Endpoints

Analysis of Safety Endpoints

Analysis of other lipid parameters (LDL, HDL and triglycerides) will generally parallel the analysis of total cholesterol.

Analysis of all other safety measures will be descriptive and will include subjects in the T analysis set. Missing observations will be excluded from analysis of safety measures.

All safety data collected on or after the date that study drug was first dispensed, up to the date of last dose of study drug plus 30 days, will be summarized by treatment group (according to the study drug received).

All available data for the pretreatment and treatment-free follow-up periods will be included in data listings.

Analysis of Questionnaire data

Descriptive statistics will be used to characterize the outcome measures (data from questionnaires). Appropriate tests of significance will be utilized to explore observed differences of interest.

8.6.3. Extent of Exposure

A subject's extent of exposure to study drug data will be generated from the study drug administration page of the eCRF. Exposure data will be summarized by treatment group.

8.6.4. Adverse Events

Clinical and laboratory adverse events will be coded using the Medical Dictionary for Regulatory Activities (MedDRA). System Organ Class (SOC), High-Level Group Term (HLGT), High-Level Term (HLT), Preferred Term (PT), and Lower-Level Term (LLT) will be attached to the clinical database.

Summaries (number and percentage of subjects) of treatment-emergent adverse events (by SOC and PT) will be provided by treatment group.

Events will be summarized on the basis of the date of the event onset. A treatment-emergent adverse event will be defined as any adverse event that begins on or after the date of first dose of study drug up to the date of last dose of study drug plus 30 days.

8.6.5. Laboratory Evaluations

Descriptive statistics will summarize selected laboratory data by the observed data and by the change from baseline across time.

Graded laboratory abnormalities will be defined using the grading scheme defined in Appendix 4. Grading of laboratory abnormalities for analysis purposes will be performed by the Biometrics department at Gilead (or designated CRO).

Incidence of treatment-emergent laboratory abnormalities, defined as values that increase at least one toxicity grade from baseline and at any time post baseline, up to and including the date of last dose of study drug plus 30 days, will be summarized by treatment group. If baseline data are missing, then any graded abnormality (i.e., at least a Grade 1) will be considered treatment emergent.

Laboratory abnormalities that occur before the first dose of study drug or after the subject has been discontinued from treatment for at least 30 days will be included in a data listing.

8.6.6. Other Safety Evaluations

Descriptive statistics will summarize other safety measure for subjects in the T analysis set.

8.7. Sample Size

Based on previous studies (RAVE study, GS-02-1008 and other studies on file), a mean difference between the two groups of about 0.5 mmol/L is expected to be observed. The pooled SD for this difference is estimated to be about 1.1 mmol/L.

It is estimated, with an assumed pooled standard deviation of 1.1 mmol/L for total cholesterol, that a sample size of 180 (90 per group) would provide approximately 85% power to detect a mean difference of 0.5 mmol/L between the two groups using a two-sided Student t-test.

9. RESPONSIBILITIES

9.1. Investigator Responsibilities

9.1.1. Good Clinical Practice

This study is subject to, and the investigator will ensure that it is conducted in accordance with, the requirements of the European Community Directive 2005/28/EC and the principles of the "Declaration of Helsinki" (Appendix 11).

9.1.2. Independent Ethics Committee (IEC) Approval

This protocol and any accompanying material to be provided to the subject (such as advertisements, subject information sheets, or descriptions of the study used to obtain informed consent) will be submitted by the Chief Investigator to an IEC. Approval from the IEC must be obtained **before** starting the study and should be documented in a letter to the Chief Investigator specifying the protocol number, protocol version, protocol date, documents reviewed, and date on which the committee met and granted the approval.

Any modifications made to the protocol after receipt of IEC approval that qualify as a 'substantial amendment' (as described in 2005/28/EC and supporting guidances) must also be submitted to the IEC for approval before implementation.

9.1.3. Informed Consent

The investigator is responsible for obtaining written informed consent from each individual participating in this study after adequate explanation of the aims, methods, objectives, and potential hazards of the study and before undertaking any study-related procedures. The investigator must utilize an IEC-approved consent form for documenting written informed consent. Each informed consent will be appropriately signed and dated by the subject or the subject's legally authorized representative and the person obtaining consent.

9.1.4. Confidentiality

The investigator must assure that subjects' anonymity will be strictly maintained and that their identities are protected from unauthorized parties. Only subject initials, date of birth and an identification code (i.e., not names) should be recorded on any form or biological sample submitted to the sponsor, IEC, or laboratory. The investigator must keep a screening log showing codes, names, and addresses for all subjects screened and for all subjects enrolled in the trial.

The investigator agrees that all information received from Gilead, including but not limited to the Investigators Brochure, this protocol, eCRFs, the investigational new drug, and any other study information, remain the sole and exclusive property of Gilead during the conduct of the study and thereafter. This information is not to be disclosed to any third party (except

employees or agents directly involved in the conduct of the study or as required by law) without prior written consent from Gilead. The investigator further agrees to take all reasonable precautions to prevent the disclosure by any employee or agent of the study site to any third party or otherwise into the public domain.

9.1.5. Study Files and Retention of Records

The investigator must maintain adequate and accurate records to enable the conduct of the study to be fully documented and the study data to be subsequently verified. These documents should be classified into at least the following two categories: (1) investigator's study file, and (2) subject clinical source documents.

The investigator's study file will contain the protocol/amendments, CRF and query forms, IEC and governmental approval with correspondence, informed consent, drug records, staff curriculum vitae and authorization forms, and other appropriate documents and correspondence.

Subject clinical source documents (usually defined by the project in advance to record key efficacy/safety parameters independent of the CRFs) would include (although not be limited to) the following: subject hospital/clinic records, physician's and nurse's notes, appointment book, original laboratory reports, electrocardiogram (ECG), electroencephalogram (EEG), X-ray, pathology and special assessment reports, consultant letters, screening and enrollment log, etc.

All clinical study documents must be retained by the investigator for at least 5 years after the investigation is discontinued and regulatory authorities have been notified. Investigators may be required to retain documents longer if required by applicable regulatory requirements or an agreement with Gilead. The investigator must notify Gilead before destroying any clinical study records.

Should the investigator wish to assign the study records to another party or move them to another location, Gilead must be notified in advance.

If the investigator cannot guarantee this archiving requirement at the study site for any or all of the documents, the investigator should arrange to store these in sealed containers outside of the site in an offsite archive facility so that they can be returned sealed to the investigator in case of a regulatory audit.

Biological samples at the conclusion of this study may be retained in storage by the sponsor for a period up to 15 years for purposes of this study.

9.1.6. Electronic Case Report Forms

For each subject enrolled, an eCRF must be completed and signed by the principal investigator or sub-investigator within a reasonable time period after data collection. This also applies to records for those subjects who fail to complete the study (even during a

pre-randomization screening period if an eCRF was initiated). If a subject withdraws from the study, the reason must be noted on the eCRF. If a subject is withdrawn from the study because of a treatment-limiting adverse event, thorough efforts should be made to clearly document the outcome.

9.1.7. Drug Accountability

The investigator or designee (i.e., pharmacist) is responsible for ensuring adequate accountability of all used and unused study drug. This includes:

- Acknowledgment of receipt of each shipment of Atripla (quantity and condition)
- Subject dispensing records. Dispensing records will document
  - Quantities of Atripla received from Gilead
  - Quantities of all study drugs dispensed to subjects, including lot number, date dispensed, subject identifier number, subject initials, and the initials of the person dispensing the medication
- Returns of all study drugs

At the end of the study, following final drug inventory reconciliation by the monitor, the study site will return all unused Atripla, including empty containers, to Gilead according to Gilead procedures.

All drug supplies and associated documentation will be periodically reviewed and verified by the study monitor over the course of the study.

9.1.8. Inspections

The investigator should understand that source documents for this trial should be made available to appropriately qualified personnel from Gilead or its representatives, to IECs, or to regulatory authority or health authority inspectors.

9.1.9. Protocol Compliance

The investigator is responsible for ensuring the study is conducted in accordance with the procedures and evaluations described in this protocol.

9.2. Sponsor Responsibilities

9.2.1. Protocol Modifications

Protocol modifications, except those intended to reduce immediate risk to study subjects, may be made only by Gilead. All protocol modifications that qualify as a ‘substantial

amendment' (as described in 2005/28/EC and supporting guidances) must be submitted to the IEC and Regulatory Authorities in accordance with local requirements. Approval must be obtained before changes can be implemented.

9.2.2. Study Report and Publications

A clinical study report will be prepared and provided to the regulatory agency(ies). Gilead will ensure that the report meets the standards set out in the ICH Guideline for Structure and Content of Clinical Study Reports (ICH E3). Note that an abbreviated report may be prepared in certain cases.

After conclusion of the study and without prior written approval from Gilead, investigators in this study may communicate, orally present, or publish in scientific journals or other scholarly media ***only after the following conditions have been met:***

- The results of the study in their entirety have been publicly disclosed by or with the consent of Gilead in an abstract, manuscript, or presentation form; or
- The study has been completed at all study sites for at least 2 years.

No such communication, presentation, or publication will include Gilead's confidential information (see Section 9.1.4).

The investigator will submit any proposed publication or presentation along with the respective scientific journal or presentation forum at least 30 days before submission of the publication or presentation. The investigator will comply with Gilead's request to delete references to its confidential information (other than the study results) in any paper or presentation and agrees to withhold publication or presentation for an additional 60 days in order to obtain patent protection if deemed necessary.

9.3. Joint Investigator/Sponsor Responsibilities

9.3.1. Access to Information for Monitoring

In accordance with ICH Good Clinical Practice (ICH GCP) guidelines, the study monitor must have direct access to the investigator's source documentation in order to verify the data recorded in the eCRFs for consistency.

The monitor is responsible for routine review of the eCRFs at regular intervals throughout the study to verify adherence to the protocol and the completeness, consistency, and accuracy of the data being entered on them. The monitor should have access to any subject records needed to verify the entries on the eCRFs. The investigator agrees to cooperate with the monitor to ensure that any problems detected in the course of these monitoring visits are resolved.

9.3.2. Access to Information for Auditing or Inspections

Representatives of regulatory authorities or of Gilead may conduct inspections or audits of the clinical study. If the investigator is notified of an inspection by a regulatory authority the investigator agrees to notify the Gilead medical monitor immediately. The investigator agrees to provide to representatives of a regulatory agency or Gilead access to records, facilities, and personnel for the effective conduct of any inspection or audit.

9.3.3. Study Discontinuation

Both the sponsor and the investigator reserve the right to terminate the study at any time. Should this be necessary, both parties will arrange discontinuation procedures and notify the appropriate regulatory authorities and IECs. In terminating the study, Gilead and the investigator will assure that adequate consideration is given to the protection of the subjects' interests.

10. REFERENCES

3551 Carr A, Workman C, Smith DE, Hoy J, Hudson J, Doong N, et al. Abacavir substitution for nucleoside analogs in patients with HIV lipoatrophy: a randomized trial. JAMA 2002;288 (2):207-15.

5117 Carr A, Samaras K, Burton S, Law M, Freund J, Chisholm DJ, et al. A syndrome of peripheral lipodystrophy, hyperlipidaemia and insulin resistance in patients receiving HIV protease inhibitors. AIDS 1998;12 (7):F51-F8.

5675 Dube MP, Stein JH, Aberg JA, Fichtenbaum CJ, Gerber JG, Tashima KT, et al. Guidelines for the evaluation and management of dyslipidemia in human immunodeficiency virus (HIV)-infected adults receiving antiretroviral therapy: recommendations of the HIV Medical Assocaition of the Infectious Disease Society of America and the Adult AIDS Clinical Trials Group. Clin Infect Dis 2003;37 (5):613-27.

5903 Friis-Moller N, Sabin CA, Weber R, d'Arminio Monforte A, El-Sadr WM, Reiss P, et al. Combination antiretroviral therapy and the risk of myocardial infarction. N Engl J Med 2003;349 (21):1993-2003.

9299 Llibre JM, Domingo P, Palacios R, Santos J, Perez-Elias MJ, Sanchez-de la Rosa R, et al. Sustained improvement of dyslipidaemia in HAART-treated patients replacing stavudine with tenofovir. AIDS 2006;20 (10):1407-14.

10159 DeJesus E, Sharma S, Corales R, Ebrahimi R, Fisher A, Flaherty J. Impact of switching virologically-suppressed, HIV-infected patients from fixed-dose zidovudine/lamivudine (CBV) to fixed-dose tenofovir DF/emtricitabine (TVD) [poster number P53]. 8th International Congress on Drug Therapy in HIV Infection; 2006 November 12-16; Glasgow, UK.

10403 UNAIDS/World Health Organization. AIDS epidemic update: December 2006. December, 2006.

10619 Galindo MJ, Miralles C, Perez-Elias MJ, Palacios R, Arazo P, Alvarez ML, et al. Switching an NRTI to tenofovir DF due to NRTI toxicity is safe and effective in both sexes [poster number P161]. 8th International Congress on Drug Therapy in HIV Infection; 2006 November 12-16; Glasgow, UK.

10958 Moyle GJ, Baldwin C, Langroudi B, Mandalia S, Gazzard BG. A 48-week, randomized, open-label comparison of three abacavir-based substitution approaches in the management of dyslipidemia and peripheral lipoatrophy. JAIDS 2003;33 (1):22-8.

10979 Grover SA, Coupal L, Gilmore N, Mukherjee J. Impact of dyslipidemia associated with Highly Active Antiretroviral Therapy (HAART) on cardiovascular risk and life expectancy. Am J Cardiol 2005;95 (5):586-91.

11. APPENDICES

Appendix 1. Investigator Signature Page
Appendix 2. Study Procedures Table
Appendix 3. Management of Clinical and Laboratory Adverse Events
Appendix 4. GSI Grading Scale for Severity of Adverse Events and Laboratory Abnormalities
Appendix 5. Patient Preference of Medicine (POM)
Appendix 6. Treatment Satisfaction and Symptoms Questionnaire (TSS)
Appendix 7. Disallowed Medications
Appendix 8. HAART Intrusiveness Scale (m-HIS)
Appendix 9. Perceived Ease of Adherence (PERC) and Visual Analogue Scale (VAS) Questionnaire
Appendix 10. National Cholesterol Education Program ATP III Guidelines
Appendix 11. World Medical Association Declaration of Helsinki

Appendix 1. Investigator Signature Page

GILEAD SCIENCES LIMITED
FLOWERS BUILDING, GRANTA PARK
ABINGTON, CAMBRIDGE, CB21 6GT
UNITED KINGDOM

STUDY ACKNOWLEDGEMENT

A Phase 4, Open Label, Randomized, Controlled Study to Assess the Effect on Lipid Profile of Switching from a Stable HAART Regimen of fixed dose Abacavir/Lamivudine (Kivexa) Plus Efavirenz, to Once Daily Atripla in Adult HIV-1 Infected Subjects With Raised Cholesterol

GS-UK-177-0109 Original Protocol, 22 November 2007

This protocol has been approved by Gilead Sciences, Inc. The following signature documents this approval.

J Ewan
Jacqueline Ewan (Printed)
Author

28-NOV-07
Date

Signature

INVESTIGATOR STATEMENT

I have read the protocol, including all appendices, and I agree that it contains all necessary details for me and my staff to conduct this study as described. I will conduct this study as outlined herein and will make a reasonable effort to complete the study within the time designated.

I will provide all study personnel under my supervision copies of the protocol and access to all information provided by Gilead Sciences, Inc. I will discuss this material with them to ensure that they are fully informed about the drugs and the study.

Principal Investigator Name (Printed) Signature

Date Site Number

Appendix 2. Study Procedures Table

Study Procedures	Screening	Baseline	Follow-Up Visits				
	Day ≤ –28	Day 0	4 Weeks	12 Weeks	16 Weeks	24 Weeks/ Early Term.	Outcome Visit[a]
Written Informed Consent	X						
Randomization		X					
Medical History	X						
Concomitant and Antiretroviral Medications	X	X	X	X	X	X	X
Adverse Events		X	X	X	X	X	X
Confirmation of HIV-1 Infection	X[b]						
Hepatitis B and C Testing	X						
Serum Pregnancy Test[c]	X	X	X[d]	X[d]	X[d]	X[d]	
Urine Pregnancy Test[c]			X	X	X	X	
FSH level[e]	X						
Physical Examination	X	X	X	X	X	X	
Vital Signs	X	X	X	X	X	X	
Height	X						
Weight	X	X	X	X	X	X	
CD4/CD8 Count and %	X	X	X	X	X	X	
Hematology	X	X	X	X	X	X	
Chemistry	X	X	X	X	X	X	
Urinalysis	X	X	X	X	X	X	
Plasma HIV RNA	X	X	X	X	X	X	
Fasting Glucose		X	X	X	X	X	
Fasting Lipid Profile –TC, LDL, HDL, non-HDL, TG, Cholesterol Ratios		X	X	X	X	X	

Study Procedures	Screening	Baseline	Follow-Up Visits				
	Day ≤ –28	Day 0	4 Weeks	12 Weeks	16 Weeks	24 Weeks/ Early Term.	Outcome Visit[a]
Calculated Creatinine Clearance	X	X	X	X	X	X	
POM Adherence Questionnaire			X[f]		X[f]		
PERC Adherence and VAS Questionnaire		X	X	X	X	X	
Acceptability Questionnaires (TSS and m-HIS)		X	X	X	X	X	
Drug Dispensing		X	X	X	X		
Pill Count			X	X	X	X	

a The investigator will contact the subject to conduct an Outcome Visit thirty days after completion or discontinuation from the study. If study medication was not returned at the last visit the subject must return to the clinic for the outcome visit and return all unused study medication (and used packaging). Subjects that do not have any study medication can be contacted by telephone in order to complete the outcome form. This telephone contact must still be considered a formal study visit.

b Only if not previously performed

c WOCP only

d Serum pregnancy test to be performed if positive urinary pregnancy test occurs

e Female subjects who have been postmenopausal for less than two years

f POM only to be completed for subjects that have switched to Atripla

Appendix 3. Management of Clinical and Laboratory Adverse Events

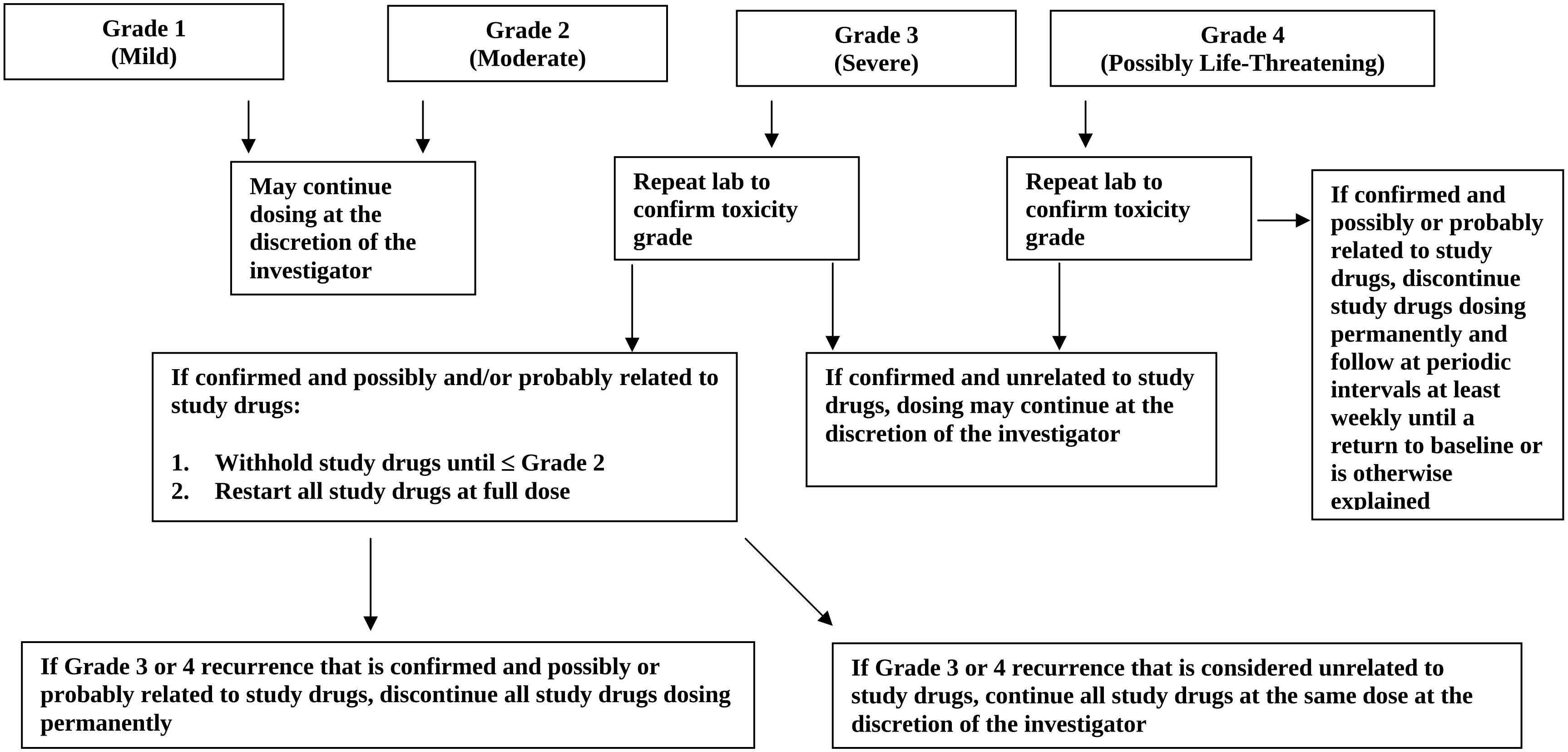

Appendix 4. GSI Grading Scale for Severity of Adverse Events and Laboratory Abnormalities

HEMATOLOGY				
	**Grade 1**	**Grade 2**	**Grade 3**	**Grade 4**
Hemoglobin<br>HIV POSITIVE<br>**Adult and Pediatric ≥ 57 Days**	8.5 to 10.0 g/dL<br>85 to 100 g/L	7.5 to < 8.5 g/dL<br>75 to < 85 g/L	6.5 to < 7.5 g/dL<br>65 to < 75 g/L	< 6.5 g/dL<br>< 65 g/L
HIV NEGATIVE<br>**Adult and Pediatric ≥ 57 Days**	10.0 to 10.9 g/dL<br>100 to 109 g/L<br>OR<br>Any decrease<br>2.5 to < 3.5 g/dL<br>25 to < 35 g/L	9.0 to < 10.0 g/dL<br>90 to < 100 g/L<br>OR<br>Any decrease<br>3.5 to < 4.5 g/dL<br>35 to < 45 g/L	7.0 to < 9.0 g/dL<br>70 to < 90 g/L<br>OR<br>Any decrease<br>≥ 4.5 g/dL<br>≥ 45 g/L	< 7.0 g/dL<br>< 70 g/L
**Infant, 36–56 Days**<br>(HIV POSITIVE OR NEGATIVE)	8.5 to 9.4 g/dL<br>85 to 94 g/L	7.0 to < 8.5 g/dL<br>70 to < 85 g/L	6.0 to < 7.0 g/dL<br>60 to < 70 g/L	< 6.0 g/dL<br>< 60 g/L
**Infant, 22–35 Days**<br>(HIV POSITIVE OR NEGATIVE)	9.5 to 10.5 g/dL<br>95 to 105 g/L	8.0 to < 9.5 g/dL<br>80 to < 95 g/L	7.0 to < 8.0 g/dL<br>70 to < 80 g/L	< 7.0 g/dL<br>< 70 g/L
**Infant, 1–21 Days**<br>(HIV POSITIVE OR NEGATIVE)	12.0 to 13.0 g/dL<br>120 to 130 g/L	10.0 to < 12.0 g/dL<br>100 to < 120 g/L	9.0 to < 10.0 g/dL<br>90 to < 100 g/L	< 9.0 g/dL<br>< 90 g/L

HEMATOLOGY				
	**Grade 1**	**Grade 2**	**Grade 3**	**Grade 4**
Absolute Neutrophil Count (ANC)<br>**Adult and Pediatric, > 7 Days**	1000 to 1300/mm^3<br>1.00 to 1.30 GI/L	750 to < 1000/mm^3<br>0.75 to < 1.00 GI/L	500 to < 750/mm^3<br>0.50 to < 0.75 GI/L	< 500/mm^3<br>< 0.50 GI/L
**Infant**, **2 – ≤ 7 Days**	1250 to 1500/mm^3<br>1.25 to 1.50 GI/L	1000 to < 1250/mm^3<br>1.00 to < 1.25 GI/L	750 to < 1000/mm^3<br>0.75 to < 1.00 GI/L	< 750/mm^3<br>< 0.75 GI/L
**Infant**, **1 Day**	4000 to 5000/mm^3<br>4.00 to 5.00 GI/L	3000 to < 4000/mm^3<br>3.00 to < 4.00 GI/L	1500 to < 3000/mm^3<br>1.50 to < 3.00 GI/L	< 1500/mm^3<br>< 1.50 GI/L
Absolute CD4+ Count<br>HIV NEGATIVE ONLY<br>**Adult and Pediatric > 13 Years**	300 to 400/mm^3<br>300 to 400/microL	200 to < 300/mm^3<br>200 to < 300/microL	100 to < 200/mm^3<br>100 to < 200/microL	< 100/mm^3<br>< 100/microL
Absolute Lymphocyte Count<br>HIV NEGATIVE ONLY<br>**Adult and Pediatric > 13 Years**	600 to 650/mm^3<br>0.60 to 0.65 GI/L	500 to < 600/mm^3<br>0.50 to < 0.60 GI/L	350 to < 500/mm^3<br>0.35 to < 0.50 GI/L	< 350/mm^3<br>< 0.35 GI/L
Platelets	100,000 to < 125,000/mm^3<br>100.0 to < 125.0 GI/L	50,000 to < 100,000/mm^3<br>50.0 to < 100.0 GI/L	25,000 to < 50,000/mm^3<br>25.0 to < 50.0 GI/L	< 25,000/mm^3<br>< 25.0 GI/L
WBCs	2000/mm^3 to 2500/mm^3<br>2.0 GI/L to 2.5 GI/L	1,500 to < 2,000/mm^3<br>1.5 to < 2.0 GI/L	1000 to < 1,500/mm^3<br>1.0 to < 1.5 GI/L	< 1000/mm^3<br>< 1.0 GI/L

HEMATOLOGY				
	**Grade 1**	**Grade 2**	**Grade 3**	**Grade 4**
Hypofibrinogenemia	100 to < LLN mg/dL<br>1.00 to < LLN g/L	75 to < 100 mg/dL<br>0.75 to <1.00 g/L	50 to < 75 mg/dL<br>0.50 to < 0.75 g/L	< 50 mg/dL<br>< 0.50 g/L
Hyperfibrinogenemia	> ULN to 600 mg/dL<br>> ULN to 6.0 g/L	> 600 mg/dL<br>> 6.0 g/L	—	—
Fibrin Split Product	20 to 40 microg/mL<br>20 to 40 mg/L	> 40 to 50 microg/mL<br>> 40 to 50 mg/L	> 50 to 60 microg/mL<br>> 50 to 60 mg/L	> 60 microg/mL<br>> 60 mg/L
Prothrombin Time (PT)	> 1.00 to 1.25 × ULN	> 1.25 to 1.50 × ULN	> 1.50 to 3.00 × ULN	> 3.00 × ULN
Activated Partial Thromboplastin (APPT)	> 1.00 to 1.66 × ULN	> 1.66 to 2.33 × ULN	> 2.33 to 3.00 × ULN	> 3.00 × ULN
Methemoglobin	5.0 to 10.0%	> 10.0 to 15.0%	> 15.0 to 20.0%	> 20.0%

CHEMISTRY				
	**Grade 1**	**Grade 2**	**Grade 3**	**Grade 4**
Hyponatremia	130 mEq/L to < LLN<br>130 mmol/L to < LLN	125 to < 130 mEq/L<br>125 to < 130 mmol/L	121 to < 125 mEq/L<br>121 to < 125 mmol/L	< 121 mEq/L<br>< 121 mmol/L
Hypernatremia	> ULN to 150 mEq/L<br>> ULN to 150 mmol/L	> 150 to 154 mEq/L<br>> 150 to 154 mmol/L	> 154 to 159 mEq/L<br>> 154 to 159 mmol/L	> 159 mEq/L<br>> 159 mmol/L
Hypokalemia	3.0 mEq/L to < LLN<br>3.0 mmol/L to < LLN	2.5 to < 3.0 mEq/L<br>2.5 to < 3.0 mmol/L	2.0 to < 2.5 mEq/L<br>2.0 to < 2.5 mmol/L	< 2.0 mEq/<br>< 2.0 mmol/L
Hyperkalemia	5.6 to 6.0 mEq/L<br>5.6 to 6.0 mmol/L	> 6.0 to 6.5 mEq/L<br>> 6.0 to 6.5 mmol/L	> 6.5 to 7.0 mEq/L<br>> 6.5 to 7.0 mmol/L	> 7.0 mEq/L<br>> 7.0 mmol/L
Hypoglycemia<br>**Adult and Pediatric**<br>**≥ 1 Month**	55 to 64 mg/dL<br>3.1 to 3.5 mmol/L	40 to < 55 mg/dL<br>2.2 to < 3.1 mmol/L	30 to < 40 mg/dL<br>1.7 to < 2.2 mmol/L	< 30 mg/dL<br>< 1.7 mmol/L
**Infant, < 1 Month**	50 to 54 mg/dL<br>2.8 to 3.0 mmol/L	40 to < 50 mg/dL<br>2.2 to < 2.8 mmol/L	30 to < 40 mg/dL<br>1.7 to < 2.2 mmol/L	< 30 mg/dL<br>< 1.7 mmol/L
Hyperglycemia, Nonfasting and No Prior Diabetes	> ULN to 160 mg/dL<br>> ULN to 8.9 mmol/L	> 160 to 250 mg/dL<br>> 8.9 to 13.9 mmol/L	> 250 to 500 mg/dL<br>> 13.9 to 27.7 mmol/L	> 500 mg/dL<br>> 27.7 mmol/L
Hypocalcemia<br>(corrected for albumin)<br>**Adult and Pediatric**<br>**≥ 7 Days**	7.8 mg/dL to < LLN<br>1.94 mmol/L to < LLN	7.0 to < 7.8 mg/dL<br>1.74 to < 1.94 mmol/L	6.1 to < 7.0 mg/dL<br>1.51 to < 1.74 mmol/L	< 6.1 mg/dL<br>< 1.51 mmol/L
**Infant, < 7 Days**	6.5 to 7.5 mg/dL<br>1.61 to 1.88 mmol/L	6.0 to < 6.5 mg/dL<br>1.49 to < 1.61 mmol/L	5.5 to < 6.0 mg/dL<br>1.36 to < 1.49 mmol/L	< 5.5 mg/dL<br>< 1.36 mmol/L

CHEMISTRY				
	**Grade 1**	**Grade 2**	**Grade 3**	**Grade 4**
Hypercalcemia (corrected for albumin) **Adult and Pediatric ≥ 7 Days**	> ULN to 11.5 mg/dL<br>> ULN to 2.88 mmol/L	> 11.5 to 12.5 mg/dL<br>> 2.88 to 3.13 mmol/L	> 12.5 to 13.5 mg/dL<br>> 3.13 to 3.38 mmol/L	> 13.5 mg/dL<br>> 3.38 mmol/L
**Infant, < 7 Days**	11.5 to 12.4 mg/dL<br>2.86 to 3.10 mmol/L	> 12.4 to 12.9 mg/dL<br>> 3.10 to 3.23 mmol/L	> 12.9 to 13.5 mg/dL<br>> 3.23 to 3.38 mmol/L	> 13.5 mg/dL<br>> 3.38 mmol/L
Hypocalcemia (ionized)	3.0 mg/dL to < LLN<br>0.74 mmol/L to < LLN	2.5 to < 3.0 mg/dL<br>0.62 to < 0.74 mmol/L	2.0 to < 2.5 mg/dL<br>0.49 to < 0.62 mmol/L	< 2.0 mg/dL<br>< 0.49 mmol/L
Hypercalcemia (ionized)	> ULN to 6.0 mg/dL<br>> ULN to 1.50 mmol/L	> 6.0 to 6.5 mg/dL<br>> 1.50 to 1.63 mmol/L	> 6.5 to 7.0 mg/dL<br>> 1.63 to 1.75 mmol/L	> 7.0 mg/dL<br>> 1.75 mmol/L
Hypomagnesemia	1.45 mg/dL to < LLN<br>0.60 mmol/L to < LLN	1.09 to < 1.45 mg/dL<br>0.45 to < 0.60 mmol/L	0.72 to < 1.09 mg/dL<br>0.30 to < 0.45 mmol/L	< 0.72 mg/dL<br>< 0.30 mmol/L
Hypophosphatemia **Adult and Pediatric > 14 Years**	2.0 mg/dL to < LLN<br>0.63 mmol/L to < LLN	1.5 to < 2.0 mg/dL<br>0.47 to < 0.63 mmol/L	1.0 to < 1.5 mg/dL<br>0.31 to < 0.47 mmol/L	< 1.0 mg/dL<br>< 0.31 mmol/L
**Pediatric 1 Year–14 Years**	3.0 to 3.5 mg/dL<br>0.96 to 1.14 mmol/L	2.5 to < 3.0 mg/dL<br>0.80 to < 0.96 mmol/L	1.5 to < 2.5 mg/dL<br>0.47 to < 0.80 mmol/L	< 1.5 mg/dL<br>< 0.47 mmol/L
**Pediatric < 1 Year**	3.5 to 4.5 mg/dL<br>1.12 to 1.46 mmol/L	2.5 to < 3.5 mg/dL<br>0.80 to < 1.12 mmol/L	1.5 to < 2.5 mg/dL<br>0.47 to < 0.80 mmol/L	< 1.5 mg/dL<br>< 0.47 mmol/L

CHEMISTRY				
	**Grade 1**	**Grade 2**	**Grade 3**	**Grade 4**
Hyperbilirubinemia<br>**Adult and Pediatric > 14 Days**	> 1.0 to 1.5 × ULN	> 1.5 to 2.5 × ULN	> 2.5 to 5.0 × ULN	> 5.0 × ULN
**Infant, ≤ 14 Days** (non-hemolytic)	NA	20.0 to 25.0 mg/dL<br>342 to 428 micromol/L	> 25.0 to 30.0 mg/dL<br>> 428 to 513 micromol/L	> 30.0 mg/dL<br>> 513 micromol/L
**Infant, ≤ 14 Days** (hemolytic)	NA	NA	20.0 to 25.0 mg/dL<br>342 to 428 micromol/L	> 25.0 mg/dL<br>> 428 micromol/L
Blood Urea Nitrogen	1.25 to 2.50 × ULN	> 2.50 to 5.00 × ULN	> 5.00 to 10.00 × ULN	> 10.00 × ULN
Hyperuricemia	> ULN to 10.0 mg/dL<br>> ULN to 597 micromol/L	> 10.0 to 12.0 mg/dL<br>> 597 to 716 micromol/L	> 12.0 to 15.0 mg/dL<br>> 716 to 895 micromol/L	> 15.0 mg/dL<br>> 895 micromol/L
Hypouricemia	1.5 mg/dL to < LLN<br>90 micromol/L to < LLN	1.0 to <1.5 mg/dL<br>60 to < 90 micromol/L	0.5 to < 1.0 mg/dL<br>30 to < 60 micromol/L	< 0.5 mg/dL<br>< 30 micromol/L
Creatinine	> 1.5 to 2.0 mg/dL<br>> 137 to 181 micromol/L	> 2.0 to 3.0 mg/dL<br>> 181 to 269 micromol/L	> 3.0 to 6.0 mg/dL<br>> 269 to 535 micromol/L	> 6.0 mg/dL<br>> 535 micromol/L
Bicarbonate	16.0 mEq/L to < LLN<br>16.0 mmol/L to < LLN	11.0 to < 16.0 mEq/L<br>11.0 to < 16.0 mmol/L	8.0 to < 11.0 mEq/L<br>8.0 to < 11.0 mmol/L	< 8.0 mEq/L<br>< 8.0 mmol/L
Triglycerides (Fasting)	—	500 to 750 mg/dL	> 750 to 1200 mg/dL	> 1200 mg/dL

CHEMISTRY				
	Grade 1	Grade 2	Grade 3	Grade 4
		5.64 – 8.47 mmol/L	> 8.47 – 13.55 mmol/L	> 13.55 mmol/L
Hypercholesterolemia<br>(Fasting)<br>**Pediatric < 18 Years**	200 to 239 mg/dL<br>5.16 to 6.19 mmol/L<br>170 to 199 mg/dL<br>4.39 to 5.15 mmol/L	> 239 to 300 mg/dL<br>> 6.19 to 7.77 mmol/L<br>> 199 to 300 mg/dL<br>> 5.15 to 7.77 mmol/L	> 300 mg/dL<br>> 7.77 mmol/L<br>> 300 mg/dL<br>> 7.77 mmol/L	—<br><br>NA
Creatine Kinase	3.0 to < 6.0 × ULN	6.0 to < 10.0 × ULN	10.0 to < 20.0 × ULN	≥ 20.0 × ULN
Lactate	ULN to < 2.0 × ULN without acidosis	≥ 2.0 × ULN without acidosis	Increased lactate with pH < 7.3 without life-threatening consequences	Increased lactate with pH < 7.3 with life-threatening consequences

ENZYMES				
	**Grade 1**	**Grade 2**	**Grade 3**	**Grade 4**
AST (SGOT)	1.25 to 2.50 × ULN	> 2.50 to 5.00 × ULN	> 5.00 to 10.00 × ULN	> 10.00 × ULN
ALT (SGPT)	1.25 to 2.50 × ULN	> 2.50 to 5.00 × ULN	> 5.00 to 10.00 × ULN	> 10.00 × ULN
GGT	1.25 to 2.50 × ULN	> 2.50 to 5.00 × ULN	> 5.00 to 10.00 × ULN	> 10.00 × ULN
Alkaline Phosphatase	1.25 to 2.50 × ULN	> 2.50 to 5.00 × ULN	> 5.00 to 10.00 × ULN	> 10.00 × ULN
Total Amylase	> 1.0 to 1.5 × ULN	> 1.5 to 2.0 × ULN	> 2.0 to 5.0 × ULN	> 5.0 × ULN
Pancreatic Amylase	> 1.0 to 1.5 × ULN	> 1.5 to 2.0 × ULN	> 2.0 to 5.0 × ULN	> 5.0 × ULN
Lipase	> 1.0 to 1.5 × ULN	> 1.5 to 3.0 × ULN	> 3.0 to 5.0 × ULN	> 5.0 × ULN
Albumin	3.0 g/dL to < LLN<br>30 g/L to < LLN	2.0 to < 3.0 g/dL<br>20 to < 30 g/L	< 2.0 g/dL<br>< 20 g/L	—

URINALYSIS				
	**Grade 1**	**Grade 2**	**Grade 3**	**Grade 4**
Hematuria (Dipstick)	1+	2+	3+	NA
Hematuria (Quantitative)	6–10 RBC/HPF	> 10 RBC/HPF	Gross, with or without clots OR with RBC casts	Transfusion indicated
Proteinuria (Dipstick)	1+	2–3+	4+	NA
Proteinuria, 24 Hour Collection				
**Adult and Pediatric ≥ 10 Years**	200 to 999 mg/24 h	> 999 to 1999 mg/24 h	> 1999 to 3500 mg/24 h	> 3500 mg/24 h
**Pediatric > 3 Mo to < 10 Years**	201 to 499 mg/m^2/24 h	> 499 to 799 mg/m^2/24 h	> 799 to 1000 mg/m^2/24 h	> 1000 mg/ m^2/24 h
Glycosuria (Dipstick)	1+	2+	3+	4+

CARDIOVASCULAR				
	**Grade 1**	**Grade 2**	**Grade 3**	**Grade 4**
Cardiac Arrhythmia (general)<br>(By ECG or physical exam)	Asymptomatic AND No intervention indicated	Asymptomatic AND Non-urgent medical intervention indicated	Symptomatic, non-life-threatening AND Non-urgent medical intervention indicated	Life-threatening arrhythmia OR Urgent intervention indicated
Cardiac-ischemia/Infarction	NA	NA	Symptomatic ischemia (stable angina) OR Testing consistent with ischemia	Unstable angina OR Acute myocardial infarction
Hemorrhage (significant acute blood loss)	NA	Symptomatic AND No transfusion indicated	Symptomatic AND Transfusion of ≤ 2 units packed RBCs (for children ≤ 10 cc/kg) indicated	Life-threatening hypotension OR Transfusion of > 2 units packed RBCs indicated (for children ≤ 10 cc/kg) indicated
Hypertension (with repeat testing at same visit)	> 140–159 mmHg systolic<br>OR<br>> 90 – 99 mmHg diastolic	> 159–179 mmHg systolic<br>OR<br>> 99–109 mmHg diastolic	> 179 mmHg systolic<br>OR<br>> 109 mmHg diastolic	Life-threatening consequences (e.g., malignant hypertension) OR Hospitalization (other than ER visit) indicated
**Pediatric ≤ 17 Years** (with repeat testing at same visit)	NA	91st – 94th percentile adjusted for age, height, and gender (systolic and/or diastolic)	≥ 95th percentile adjusted for age, height, and gender (systolic and/or diastolic)	Life-threatening consequences (e.g., malignant hypertension) OR Hospitalization indicated (other than emergency room visit)
Hypotension	NA	Symptomatic, corrected with oral fluid replacement	Symptomatic, IV fluids indicated	Shock requiring use of vasopressors or mechanical assistance to maintain blood pressure
Pericardial Effusion	Asymptomatic, small effusion requiring no intervention	Asymptomatic, moderate or larger effusion requiring no intervention	Effusion with non-life-threatening physiologic consequences OR Effusion with nonurgent intervention indicated	Life-threatening consequences (e.g., tamponade) OR Urgent intervention indicated

CARDIOVASCULAR				
	**Grade 1**	**Grade 2**	**Grade 3**	**Grade 4**
Prolonged PR Interval	PR interval<br>0.21 to 0.25 sec	PR interval<br>> 0.25 sec	Type II 2nd degree AV block OR Ventricular pause > 3.0 sec	Complete AV block
**Pediatric ≤ 16 Years**	1st degree AV block<br>(PR > normal for age and rate)	Type I 2nd degree AV block	Type II 2nd degree AV block	Complete AV block
Prolonged QTc	Asymptomatic, QTc interval 0.45 to 0.47 sec OR Increase interval < 0.03 sec above baseline	Asymptomatic, QTc interval 0.48 to 0.49 sec OR Increase in interval 0.03 to 0.05 sec above baseline	Asymptomatic, QTc interval ≥ 0.50 sec OR Increase in interval ≥ 0.06 sec above baseline	Life-threatening consequences, e.g., Torsade de pointes or other associated serious ventricular dysrhythmia
**Pediatric ≤ 16 Years**	Asymptomatic, QTc interval 0.450 to 0.464 sec	Asymptomatic, QTc interval 0.465 to 0.479 sec	Asymptomatic, QTc interval ≥ 0.480 sec	Life-threatening consequences, e.g., Torsade de pointes or other associated serious ventricular dysrhythmia
Thrombosis/Embolism	NA	Deep vein thrombosis AND No intervention indicated (e.g., anticoagulation, lysis filter, invasive procedure)	Deep vein thrombosis AND Intervention indicated (e.g., anticoagulation, lysis filter, invasive procedure)	Embolic event (e.g., pulmonary embolism, life-threatening thrombus)
Vasovagal Episode (associated with a procedure of any kind)	Present without loss of consciousness	Present with transient loss of consciousness	NA	NA
Ventricular Dysfunction (congestive heart failure, CHF)	NA	Asymptomatic diagnostic finding AND intervention indicated	New onset with symptoms OR Worsening symptomatic CHF	Life-threatening CHF

RESPIRATORY				
	**Grade 1**	**Grade 2**	**Grade 3**	**Grade 4**
Bronchospasm (acute)	FEV1 or peak flow reduced to 70% to 80%	FEV1 or peak flow 50% to 69%	FEV1 or peak flow 25% to 49%	Cyanosis OR FEV1 or peak flow < 25% OR Intubation
Dyspnea or Respiratory distress	Dyspnea on exertion with no or minimal interference with usual social & functional activities	Dyspnea on exertion causing greater than minimal interference with usual social & functional activities	Dyspnea at rest causing inability to perform usual social & functional activities	Respiratory failure with ventilatory support indicated
**Pediatric < 14 Years**	Wheezing OR minimal increase in respiratory rate for age	Nasal flaring OR Intercostal retractions OR Pulse oximetry 90% to 95%	Dyspnea at rest causing inability to perform usual social & functional activities OR Pulse oximetry < 90%	Respiratory failure with ventilatory support indicated

OCULAR/VISUAL				
	**Grade 1**	**Grade 2**	**Grade 3**	**Grade 4**
Uveitis	Asymptomatic but detectable on exam	Symptomatic anterior uveitis OR Medical intervention indicated	Posterior or pan-uveitis OR Operative intervention indicated	Disabling visual loss in affected eye(s)
Visual Changes (from baseline)	Visual changes causing no or minimal interference with usual social & functional activities	Visual changes causing greater than minimal interference with usual social & functional activities	Visual changes causing inability to perform usual social & functional activities	Disabling visual loss in affected eye(s)

SKIN				
	**Grade 1**	**Grade 2**	**Grade 3**	**Grade 4**
Alopecia	Thinning detectable by study participant or caregiver (for disabled adults)	Thinning or patchy hair loss detectable by health care provider	Complete hair loss	NA
Cutaneous Reaction–Rash	Localized macular rash	Diffuse macular, maculopapular, or morbilliform rash OR Target lesions	Diffuse macular, maculopapular, or morbilliform rash with vesicles or limited number of bullae OR Superficial ulcerations of mucous membrane limited to one site	Extensive or generalized bullous lesions OR Stevens-Johnson syndrome OR Ulceration of mucous membrane involving two or more distinct mucosal sites OR Toxic epidermal necrolysis (TEN)
Hyperpigmentation	Slight or localized	Marked or generalized	NA	NA
Hypopigmentation	Slight or localized	Marked or generalized	NA	NA
Pruritis (itching – no skin lesions)<br>(See also Injection Site Reactions: Pruritis associated with injection)	Itching causing no or minimal interference with usual social & functional activities	Itching causing greater than minimal interference with usual social & functional activities	Itching causing inability to perform usual social & functional activities	NA

GASTROINTESTINAL				
	**Grade 1**	**Grade 2**	**Grade 3**	**Grade 4**
Anorexia	Loss of appetite without decreased oral intake	Loss of appetite associated with decreased oral intake without significant weight loss	Loss of appetite associated with significant weight loss	Life-threatening consequences OR Aggressive intervention indicated [e.g., tube feeding or total parenteral nutrition]
Ascites	Asymptomatic	Symptomatic AND Intervention indicated (e.g., diuretics or therapeutic paracentesis)	Symptomatic despite intervention	Life-threatening consequences
Cholecystitis	NA	Symptomatic AND Medical intervention indicated	Radiologic, endoscopic, or operative intervention indicated	Life-threatening consequences (e.g., sepsis or perforation)
Constipation	NA	Persistent constipation requiring regular use of dietary modifications, laxatives, or enemas	Obstipation with manual evacuation indicated	Life-threatening consequences (e.g., obstruction)
Diarrhea				
**Adult and Pediatric ≥ 1 Year**	Transient or intermittent episodes of unformed stools OR Increase of ≤ 3 stools over baseline/24 hr	Persistent episodes of unformed to watery stools OR Increase of 4–6 stools over baseline per 24 hrs.	Bloody diarrhea OR Increase of ≥ 7 stools per 24-hour period OR IV fluid replacement indicated	Life-threatening consequences (e.g., hypotensive shock)
**Pediatric < 1 Year**	Liquid stools (more unformed than usual) but usual number of stools	Liquid stools with increased number of stools OR Mild dehydration	Liquid stools with moderate dehydration	Liquid stools resulting in severe dehydration with aggressive rehydration indicated OR Hypotensive shock
Dysphagia-Odynophagia	Symptomatic but able to eat usual diet	Symptoms causing altered dietary intake without medical intervention indicated	Symptoms causing severely altered dietary intake with medical intervention indicated	Life-threatening reduction in oral intake

GASTROINTESTINAL				
	**Grade 1**	**Grade 2**	**Grade 3**	**Grade 4**
Mucositis/stomatitis (clinical exam)<br>See also Proctitis, Dysphagia-Odynophagia,	Erythema of the mucosa	Patchy pseudomembranes or ulcerations	Confluent pseudomembranes or ulcerations OR Mucosal bleeding with minor trauma	Tissue necrosis OR Diffuse spontaneous mucosal bleeding OR Life-threatening consequences (e.g., aspiration, choking)
Nausea	Transient (< 24 hours) or intermittent nausea with no or minimal interference with oral intake	Persistent nausea resulting in decreased oral intake for 24–48 hours	Persistent nausea resulting in minimal oral intake for > 48 hours OR Aggressive rehydration indicated (e.g., IV fluids)	Life-threatening consequences (e.g., hypotensive shock)
Pancreatitis	NA	Symptomatic AND Hospitalization not indicated (other than ER visit)	Symptomatic AND Hospitalization indicated (other than ER visit)	Life-threatening consequences (e.g., sepsis, circulatory failure, hemorrhage
Proctitis (functional-symptomatic)<br>Also see Mucositis/ Stomatitis for Clinical Exam	Rectal discomfort AND No intervention indicated	Symptoms causing greater than minimal interference with usual social & functional activities OR Medical intervention indicated	Symptoms causing inability to perform usual social/functional activities OR Operative intervention indicated	Life-threatening consequences (e.g., perforation)
Vomiting	Transient or intermittent vomiting with no or minimal interference with oral intake	Frequent episodes of vomiting with no or mild dehydration	Persistent vomiting resulting in orthostatic hypotension OR Aggressive rehydration indicated	Life-threatening consequences (e.g., hypotensive shock)

NEUROLOGICAL				
	**Grade 1**	**Grade 2**	**Grade 3**	**Grade 4**
Alteration in Personality-behavior or in Mood (e.g., agitation, anxiety, depression, mania, psychosis)	Alteration causing no or minimal interference with usual social & functional activities	Alteration causing greater than minimal interference with usual social & functional activities	Alteration causing inability to perform usual social & functional activities	Behavior potentially harmful to self or others (e.g., suicidal/homicidal ideation or attempt, acute psychosis) OR Causing inability to perform basic self-care functions
Altered Mental Status<br>For Dementia, see Cognitive and Behavioral/Attentional Disturbance (including dementia and ADD)	Changes causing no or minimal interference with usual social & functional activities	Mild lethargy or somnolence causing greater than minimal interference with usual social & functional activities	Confusion, memory impairment, lethargy, or somnolence causing inability to perform usual social & functional activities	Delirium OR obtundation, OR coma
Ataxia	Asymptomatic ataxia detectable on exam OR Minimal ataxia causing no or minimal interference with usual social & functional activities	Symptomatic ataxia causing greater than minimal interference with usual social & functional activities	Symptomatic ataxia causing inability to perform usual social & functional activities	Disabling ataxia causing inability to perform basic self-care functions
Cognitive and Behavioral/Attentional Disturbance (including dementia and Attention Deficit Disorder)	Disability causing no or minimal interference with usual social & functional activities OR Specialized resources not indicated	Disability causing greater than minimal interference with usual social & functional activities OR Specialized resources on part-time basis indicated	Disability causing inability to perform usual social & functional activities OR Specialized resources on a full-time basis indicated	Disability causing inability to perform basic self-care functions OR Institutionalization indicated
CNS Ischemia (acute)	NA	NA	Transient ischemic attack	Cerebral vascular accident (CVA, stroke) with neurological deficit
Developmental Delay – **Pediatric ≤ 16 Years**	Mild developmental delay, either motor or cognitive, as determined by comparison with a developmental screening tool appropriate for the setting	Moderate developmental delay, either motor or cognitive, as determined by comparison with a developmental screening tool appropriate for the setting	Severe developmental delay, either motor or cognitive, as determined by comparison with a developmental screening tool appropriate for the setting	Developmental regression, either motor or cognitive, as determined by comparison with a developmental screening tool appropriate for the setting

NEUROLOGICAL				
	**Grade 1**	**Grade 2**	**Grade 3**	**Grade 4**
Headache	Symptoms causing no or minimal interference with usual social & functional activities	Symptoms causing greater than minimal interference with usual social & functional activities	Symptoms causing inability to perform usual social & functional activities	Symptoms causing inability to perform basic self-care functions OR Hospitalization indicated (other than ER visit) OR Headache with significant impairment of alertness or other neurologic function
Insomnia	NA	Difficulty sleeping causing greater than minimal interference with usual social/functional activities	Difficulty sleeping causing inability to perform usual social & functional activities	Disabling insomnia causing inability to perform basic self-care functions
Neuromuscular Weakness (including myopathy & neuropathy)	Asymptomatic with decreased strength on exam OR Minimal muscle weakness causing no or minimal interference with usual social & functional activities	Muscle weakness causing greater than minimal interference with usual social & functional activities	Muscle weakness causing inability to perform usual social & functional activities	Disabling muscle weakness causing inability to perform basic self-care functions OR Respiratory muscle weakness impairing ventilation
Neurosensory Alteration (including paresthesia and painful neuropathy)	Asymptomatic with sensory alteration on exam or minimal paresthesia causing no or minimal interference with usual social & functional activities	Sensory alteration or paresthesia causing greater than minimal interference with usual social & functional activities	Sensory alteration or paresthesia causing inability to perform usual social & functional activities	Disabling sensory alteration or paresthesia causing inability to perform basic self-care functions
Seizure: (new onset)	NA	1 seizure	2–4 seizures	Seizures of any kind that are prolonged, repetitive (e.g., status epilepticus), or difficult to control (e.g., refractory epilepsy)

NEUROLOGICAL				
	**Grade 1**	**Grade 2**	**Grade 3**	**Grade 4**
Seizure: (pre-existing)<br>For Worsening of Existing Epilepsy the Grades Should be Based on an Increase from Previous Level of Control to Any of These Levels.	NA	Increased frequency of pre-existing seizures (non-repetitive) without change in seizure character OR infrequent breakthrough seizures while on stable meds in a previously controlled seizure disorder	Change in seizure character from baseline either in duration or quality (e.g., severity or focality)	Seizures of any kind that are prolonged, repetitive (e.g., status epilepticus), or difficult to control (e.g., refractory epilepsy)
Seizure<br>**– Pediatric < 18 Years**	Seizure, generalized onset with or without secondary generalization, lasting < 5 minutes with < 24 hours post ictal state	Seizure, generalized onset with or without secondary generalization, lasting 5–20 minutes with < 24 hours post ictal state	Seizure, generalized onset with or without secondary generalization, lasting > 20 minutes	Seizure, generalized onset with or without secondary generalization, requiring intubation and sedation
Syncope (not associated with a procedure)	NA	Present	NA	NA
Vertigo	Vertigo causing no or minimal interference with usual social & functional activities	Vertigo causing greater than minimal interference with usual social & functional activities	Vertigo causing inability to perform usual social & functional activities	Disabling vertigo causing inability to perform basic self-care functions

MUSCULOSKELETAL				
	**Grade 1**	**Grade 2**	**Grade 3**	**Grade 4**
Arthralgia<br>See also Arthritis	Joint pain causing no or minimal interference with usual social & functional activities	Joint pain causing greater than minimal interference with usual social & functional activities	Joint pain causing inability to perform usual social & functional activities	Disabling joint pain causing inability to perform basic self-care functions
Arthritis<br>See also Arthralgia	Stiffness or joint swelling causing no or minimal interference with usual social & functional activities	Stiffness or joint swelling causing greater than minimal interference with usual social & functional activities	Stiffness or joint swelling causing inability to perform usual social & functional activities	Disabling joint stiffness or swelling causing inability to perform basic self-care functions
Bone Mineral Loss	BMD t-score or z-score –2.5 to –1.0	BMD t-score or z-score < –2.5	Pathological fracture (including loss of vertebral height)	Pathologic fracture causing life-threatening consequences
**Pediatric < 21 Years**	BMD z-score –2.5 to –1.0	BMD z-score < –2.5	Pathological fracture (including loss of vertebral height)	Pathologic fracture causing life-threatening consequences
Myalgia (non-injection site)	Muscle pain causing no or minimal interference with usual social & functional activities	Muscle pain causing greater than minimal interference with usual social & functional activities	Muscle pain causing inability to perform usual social & functional activities	Disabling muscle pain causing inability to perform basic self-care functions
Osteonecrosis	NA	Asymptomatic with radiographic findings AND No operative intervention indicated	Symptomatic bone pain with radiographic findings OR Operative intervention indicated	Disabling bone pain with radiographic findings causing inability to perform basic self-care functions

SYSTEMIC				
	**Grade 1**	**Grade 2**	**Grade 3**	**Grade 4**
Acute Systemic Allergic Reaction	Localized urticaria (wheals) with no medical intervention indicated	Localized urticaria with medical intervention indicated OR Mild angioedema with no medical intervention indicated	Generalized urticaria OR Angioedema with medical intervention indicated OR Symptomatic mild bronchospasm	Acute anaphylaxis OR Life-threatening bronchospasm OR laryngeal edema
Chills	Symptoms causing no or minimal interference with usual social & functional activities	Symptoms causing greater than minimal interference with usual social & functional activities	Symptoms causing inability to perform usual social & functional activities	NA
Fatigue<br>Malaise	Symptoms causing no or minimal interference with usual social & functional activities	Symptoms causing greater than minimal interference with usual social & functional activities	Symptoms causing inability to perform usual social & functional activities	Incapacitating fatigue/ malaise symptoms causing inability to perform basic self-care functions
Fever (nonaxillary)	37.7°C to 38.6°C<br>99.8°F to 101.5°F	38.7°C to 39.3°C<br>101.6°F to 102.8°F	39.4°C to 40.5°C<br>102.9°F to 104.9°F	> 40.5°C<br>> 104.9°F
Pain- Indicate Body Site<br>See also Injection Site Pain, Headache, Arthralgia, and Myalgia	Pain causing no or minimal interference with usual social & functional activities	Pain causing greater than minimal interference with usual social & functional activities	Pain causing inability to perform usual social & functional activities	Disabling pain causing inability to perform basic self-care functions OR Hospitalization (other than ER visit) indicated
Unintentional Weight Loss	NA	5% to 9% loss in body weight from baseline	10% to 19% loss in body weight from baseline	≥ 20% loss in body weight from baseline OR Aggressive intervention indicated [e.g., tube feeding or total parenteral nutrition]

**INJECTION SITE REACTION**				
	**Grade 1**	**Grade 2**	**Grade 3**	**Grade 4**
Injection Site Pain (pain without touching)<br>Or Tenderness (pain when area is touched)	Pain/tenderness causing no or minimal limitation of use of limb	Pain/tenderness limiting use of limb OR Pain/tenderness causing greater than minimal interference with usual social & functional activities	Pain/tenderness causing inability to perform usual social & functional activities	Pain/tenderness causing inability to perform basic self-care function OR Hospitalization (other than ER visit) indicated for management of pain/tenderness
Injection Site Reaction (Localized), > 15 Years<br><br>**Pediatric ≤ 15 Years**	Erythema OR Induration of 5x5 cm to 9x9 cm (or 25-81cm^2)<br><br>**Erythema OR Induration OR Edema present but ≤ 2.5 cm diameter**	Erythema OR Induration OR Edema > 9 cm any diameter (or > 81 cm^2)<br><br>**Erythema OR Induration OR Edema > 2.5 cm diameter but < 50% surface area of the extremity segment (e.g., upper arm/thigh)**	Ulceration OR Secondary infection OR Phlebitis OR Sterile abscess OR Drainage<br><br>**Erythema OR Induration OR Edema involving ≥ 50% surface area of the extremity segment (e.g., upper arm/thigh) OR Ulceration OR Secondary infection OR Phlebitis OR Sterile abscess OR Drainage**	Necrosis (involving dermis and deeper tissue)<br><br>**Necrosis (involving dermis and deeper tissue)**
Pruritis Associated with Injection<br>See also Skin: Pruritis (itching—no skin lesions)	Itching localized to injection site AND Relieved spontaneously or with < 48 h treatment	Itching beyond the injection site but not generalized OR Itching localized to injection site requiring ≥ 48 h treatment	Generalized itching causing inability to perform usual social & functional activities	NA

ENDOCRINE/METABOLIC				
	**Grade 1**	**Grade 2**	**Grade 3**	**Grade 4**
Lipodystrophy (e.g., back of neck, breasts, abdomen)	Detectable by study participant or caregiver (for young children and disabled adults)	Detectable on physical exam by health care provider	Disfiguring OR Obvious changes on casual visual inspection	NA
Diabetes Mellitus	NA	New onset without need to initiate medication OR Modification of current meds to regain glucose control	New onset with initiation of indicated med OR Diabetes uncontrolled despite treatment modification	Life-threatening consequences (e.g., ketoacidosis, hyperosmolar non-ketotic coma)
Gynecomastia	Detectable by study participant or caregiver (for young children and disabled adults)	Detectable on physical exam by health care provider	Disfiguring OR Obvious on casual visual inspection	NA
Hyperthyroidism	Asymptomatic	Symptomatic causing greater than minimal interference with usual social & functional activities OR Thyroid suppression therapy indicated	Symptoms causing inability to perform usual social & functional activities OR Uncontrolled despite treatment modification	Life-threatening consequences (e.g., thyroid storm)
Hypothyroidism	Asymptomatic	Symptomatic causing greater than minimal interference with usual social & functional activities OR Thyroid replacement therapy indicated	Symptoms causing inability to perform usual social & functional activities OR Uncontrolled despite treatment modification	Life-threatening consequences (e.g., myxedema coma)
Lipoatrophy (e.g., fat loss from the face, extremities, buttocks)	Detectable by study participant or caregiver (for young children and disabled adults)	Detectable on physical exam by health care provider	Disfiguring OR Obvious on casual visual inspection	NA

GENITOURINARY				
	**Grade 1**	**Grade 2**	**Grade 3**	**Grade 4**
Intermenstrual Bleeding (IMB)	Spotting observed by participant OR Minimal blood observed during clinical or colposcopic exam	Intermenstrual bleeding not greater in duration or amount than usual menstrual cycle	Intermenstrual bleeding greater in duration or amount than usual menstrual cycle	Hemorrhage with life-threatening hypotension OR Operative intervention indicated
Urinary Tract Obstruction (e.g., stone)	NA	Signs or symptoms of urinary tract obstruction without hydronephrosis or renal dysfunction	Signs or symptoms of urinary tract obstruction with hydronephrosis or renal dysfunction	Obstruction causing life-threatening consequences

INFECTION				
	**Grade 1**	**Grade 2**	**Grade 3**	**Grade 4**
Infection (any other than HIV infection)	Localized, no systemic antimicrobial treatment indicated AND Symptoms causing no or minimal interference with usual social & functional activities	Systemic antimicrobial treatment indicated OR Symptoms causing greater than minimal interference with usual social & functional activities	Systemic antimicrobial treatment indicated AND Symptoms causing inability to perform usual social & functional activities OR Operative intervention (other than simple incision and drainage) indicated	Life-threatening consequences (e.g., septic shock)

Basic Self-care Functions: Activities such as bathing, dressing, toileting, transfer/movement, continence, and feeding.

Usual Social & Functional Activities: Adaptive tasks and desirable activities, such as going to work, shopping, cooking, use of transportation, pursuing a hobby, etc.

Appendix 5. Patient Preference of Medicine (POM)

For Treatment Group 1 (switch to Atripla tablet) Patients Only

How easy did you find it to follow your current HIV medication regimen? Check one answer.

☐ 1. much better, I prefer this medication

☐ 2. slightly better

☐ 3. about the same

☐ 4. slightly worse

☐ 5. much worse, I much prefer my previous medication

Appendix 6. Treatment Satisfaction and Symptoms Questionnaire (TSS)

Treatment Satisfaction and Symptoms Questionnaire (TSS)

To be completed by the Patient. Please use blue or black ink.

Please circle one response for each question.

A. The following questions ask about your satisfaction with your current treatment regimen:

Please circle one response for each question.

	Very Dissatisfied	Somewhat Dissatisfied	Somewhat Satisfied	Very Satisfied
1. In general, how satisfied are you with the convenience and simplicity of your current treatment regimen?	0	1	2	3
2. In general, how satisfied are you with the ability of your current treatment regimen to control your HIV infection?	0	1	2	3
3. In general, how satisfied are you with your ability to tolerate your current treatment regimen?	0	1	2	3
4. In general, how satisfied are you with your current treatment regimen?	0	1	2	3

	Does not bother me	Bothers me a little bit	Bothers me a lot	Bothers me terribly
5. How bothered are you by the side effects of your current treatment regimen?	0	1	2	3

B. The following questions ask about symptoms you might have had during the past month
Please circle one response for each question.

	I don't have this symptom	I have this symptom and it…			
		Does not bother me	Bothers me a little bit	Bothers me a lot	Bothers me terribly
1. Fatigue or loss of energy?	0	1	2	3	4
2. Fevers, chills or sweats?	0	1	2	3	4
3. Feeling dizzy or lightheaded?	0	1	2	3	4
4. Pain, numbness or tingling in the hands or feet?	0	1	2	3	4
5. Trouble remembering?	0	1	2	3	4
6. Nausea or vomiting?	0	1	2	3	4
7. Diarrhea or loose bowel movements?	0	1	2	3	4
8. Felt sad, down or depressed?	0	1	2	3	4
9. Felt nervous or anxious?	0	1	2	3	4
10. Difficulty falling or staying asleep?	0	1	2	3	4
11. Skin problems, such as rash, dryness or itching?	0	1	2	3	4
12. Cough or trouble catching your breath?	0	1	2	3	4
13. Headache?	0	1	2	3	4
14. Loss of appetite or a change in the taste of food?	0	1	2	3	4
15. Bloating, pain or gas in your stomach?	0	1	2	3	4
16. Muscle aches or joint pain?	0	1	2	3	4
17. Problems with having sex, such as loss of interest or lack of satisfaction?	0	1	2	3	4
18. Changes in the way your body looks, such as fat deposits or weight gain?	0	1	2	3	4
19. Problems with weight loss or wasting?	0	1	2	3	4
20. Hair loss or changes in the way your hair looks?	0	1	2	3	4

Thank you!

Appendix 7. Disallowed Medications

The following agents should be avoided during the course of this study.

Drugs/ Drug classes that Interact with ATRIPLA (CCSI 06 Mar 07)	Examples of Nephrotoxic Agents	Drugs/ Drug classes that interact with KIVEXA (UK SPC 27 Aug 07)	Drugs/ Drug Classes that interact with Efavirenz (UK SPC Sep 07)
Terfenadine	Aminoglycoside	Rifampicin	Terfenadine
Astemizole	Antibiotics:	Phenobarbital	Astemizole
Cisapride	Amikacin	Phenytoin	Cisapride
Midazolam	Gentamycin	Ethanol	Midazolam
Triazolam	Kanamycin	Retinoid compounds	Triazolam
Pimozide	Netromycin	Methadone	Pimozide
Bepridil	Paromomycin	Trimethoprim	Bepridil
Ergot Derivatives	Tobramycin	Trimethoprim/	Ergot alkaloids:
Voriconazole	Spectinomycin	sulfamethazole	Ergotamine
Lamivudine[1]	Streptomycin	IV ganiciclovir	Dihydroergotamine
Nephrotoxic agents	IV Amphotericin B	IV foscarnet	Ergonovine
Amprenavir	Cidofovir	Zalcitabine	Methylergonovine
Fosamprenavir	Cisplatin	Ribavirin[3]	St John's Wort
Atazanavir/ Ritonavir	Foscarnet	Interferon-alpha[3]	Voriconazole
Indinavir	IV Pentamidine		Amprenavir
Lopinavir/ Ritonavir	IL-2		Atazanavir
Saquinavir[2]	Ganciclovir		Indinavir
Didanosine			Lopinavir
Clarithromycin			Nelfinavir
Rifabutin			Ritonavir
Rifampicin			Saquinavir[1]
Itraconazole			Rifampicin
Carbamazepine			Rifabutin
Phenytoin			Clarithromycin
Phenobarbital			Itraconazole
Atorvastatin			Carbamazepine
Simvastatin			Phenytoin
Pravastatin			Phenobarbital

Diltiazem<br>Other Ca Channel Blockers<br>Sertraline<br>Methadone<br>St John's Wort			Atorvastatin<br>Pravastatin<br>Simvastatin<br>Methadone<br>Sertraline<br>Cetirizine<br>Lorazepam<br>Diltiazem<br>Other Ca Channel Blockers

[1]Medicinal products containing lamivudine as a component are contraindicated with Atripla therapy.

[2]Use of saquinavir as a sole protease inhibitor with Atripla is not recommended.

[3]Regarding lactic acidosis: Caution should be exercised when administering nucleoside analogues to any patient (particularly obese women) with hepatomegaly, hepatitis or other known risk factors for liver disease and hepatic steatosis (including certain medicinal products and alcohol). Patients co-infected with hepatitis C and treated with alpha-interferon and ribavirin may constitute a special risk.

Other medicinal products containing active components of Atripla are contraindicated with Atripla therapy. In addition, use of adefovir is contraindicated on the CCSI for tenofovir (14 Sept 07) and the drugs didanosine and atazanavir (if unboosted) are not recommended.

Appendix 8. HAART Intrusiveness Scale (m-HIS)

HAART Intrusiveness Scale (m-HIS)

(Horne et al 2002)

In answering the following questions we would like you to think about the problems you may experience with your anti-HIV combination therapy regimen (e.g. timing of doses, complexity of the regimen, side-effects, etc.).*There are no right or wrong answers and this questionnaire is completely confidential*. Under no circumstance will your answers be shown to your doctor or anyone else involved in your care.

Below are listed some statements that other HIV-infected people have made about problems with their anti-HIV combination therapy regimen.

Please show below how much these problems interfere with your own life on a scale of 1 (low interference) to 5 (high interference).

Please tick only one box for each statement

	"MY ANTIVIRAL THERAPY REGIMEN:"	DEGREE OF INTERFERENCE WITH YOUR LIFE				
		**1 Low**	**2**	**3**	**4**	**5 High**
MH1	Interferes with my social life	1 ☐	2 ☐	3 ☐	4 ☐	5 ☐
MH2	Is difficult to fit around meals	1 ☐	2 ☐	3 ☐	4 ☐	5 ☐
MH3	Makes it difficult to be spontaneous	1 ☐	2 ☐	3 ☐	4 ☐	5 ☐
MH4	Is too complex to stick to	1 ☐	2 ☐	3 ☐	4 ☐	5 ☐
MH4	Restrict my ability to travel	1 ☐	2 ☐	3 ☐	4 ☐	5 ☐
MH5	Interferes with my work	1 ☐	2 ☐	3 ☐	4 ☐	5 ☐
MH6	Makes it harder to meet friends	1 ☐	2 ☐	3 ☐	4 ☐	5 ☐
MH7	Interferes with my relationships	1 ☐	2 ☐	3 ☐	4 ☐	5 ☐
MH8	Is difficult to swallow	1 ☐	2 ☐	3 ☐	4 ☐	5 ☐
MH9	Is difficult to remember to take on time	1 ☐	2 ☐	3 ☐	4 ☐	5 ☐

Thank you very much for your cooperation!

Appendix 9. Perceived Ease of Adherence (PERC) and Visual Analogue Scale (VAS) Questionnaire

Perceived Ease of Adherence (PERC) and Visual Analogue Scale (VAS)

Medications vary with respect to number of pills, frequency and timing of taking medication, and other instructions.

How easy did you find it to follow your current HIV medication regimen? *Check one answer.*

☐ 1. very easy

☐ 2. somewhat easy

☐ 3. somewhat difficult

☐ 4. very difficult

VAS Adherence Questionnaire

We understand that many people find it very difficult to take their prescribed medication regularly and often miss doses. We will not be surprised if you have missed some doses as well. In order to find out how often people miss taking their medication, we need to ask you a few questions.

1. Put a cross on the line below at the point showing your best guess about how much of your prescribed anti- HIV medication you have taken in the last 30 days.

0% means you have taken none of the prescribed anti-HIV medication

0% 10% 20% 30% 40% 50% 60% 70% 80% 90% 100%

100% means you have taken every single dose of every prescribed anti-HIV medication

Please enter single digit numbers with leading zeros (e.g. 02 for 2 or 07 for 7) in each box for the following questions.

2. In the past 30 days, how many days have you missed any dose of your prescribed anti-HIV medication?

Enter number of days: ☐☐

3. In the past 7 days, how many days have you missed any dose of your prescribed anti-HIV medication?

Enter number of days: ☐☐

Appendix 10. National Cholesterol Education Program ATP III Guidelines

Adult Treatment Panel (ATP) III Classification of LDL, Total and HDL Cholesterol (mg/dL)*

LDL Cholesterol

mg/dL	mmol/L*	Classification
< 100	< 2.6	Optimal
100–129	2.6–3.3	Near optimal
130–159	3.4–4.1	Borderline high
160–189	4.2–4.9	High
≥ 190	≥ 5.0	Very high

*1 mmol/L = 38.67 mg/dL

Total Cholesterol

mg/dL	mmol/L*	Classification
< 200	< 5.2	Desirable
200–239	5.2–6.2	Borderline high
≥ 240	≥ 6.3	High

*1 mmol/L = 38.5 mg/dL

HDL Cholesterol

mg/dL	mmol/L*	Classification
< 40	< 1.0	Low
≥ 60	≥ 1.5	High

*1 mmol/L = 38.67 mg/dL

*All values taken from ATP III At-A-Glance: Quick Desk Reference on 18-Oct-07. NCEP values are given in mg/dL – conversion to SI units accurate to 1 decimal place.

Appendix 11. World Medical Association Declaration of Helsinki

WORLD MEDICAL ASSOCIATION DECLARATION OF HELSINKI

Ethical Principles for Medical Research Involving Human Subjects

Adopted by the 18th WMA General Assembly, Helsinki, Finland, June 1964, and amended by the

29th WMA General Assembly, Tokyo, Japan, October 1975

35th WMA General Assembly, Venice, Italy, October 1983

41st WMA General Assembly, Hong Kong, September 1989

48th WMA General Assembly, Somerset West, Republic of South Africa, October 1996

and the 52nd WMA General Assembly, Edinburgh, Scotland, October 2000

Note of Clarification on Paragraph 29 added by the WMA General Assembly, Washington 2002

Note of Clarification on Paragraph 30 added by the WMA General Assembly, Tokyo 2004

A. INTRODUCTION

1. The World Medical Association has developed the Declaration of Helsinki as a statement of ethical principles to provide guidance to physicians and other participants in medical research involving human subjects. Medical research involving human subjects includes research on identifiable human material or identifiable data.

2. It is the duty of the physician to promote and safeguard the health of the people. The physician's knowledge and conscience are dedicated to the fulfillment of this duty.

3. The Declaration of Geneva of the World Medical Association binds the physician with the words, “The health of my patient will be my first consideration,” and the International Code of Medical Ethics declares that, “A physician shall act only in the patient’s interest when providing medical care which might have the effect of weakening the physical and mental condition of the patient.”

4. Medical progress is based on research which ultimately must rest in part on experimentation involving human subjects.

5. In medical research on human subjects, considerations related to the well-being of the human subject should take precedence over the interests of science and society.

6. The primary purpose of medical research involving human subjects is to improve prophylactic, diagnostic and therapeutic procedures and the understanding of the aetiology and pathogenesis of disease. Even the best proven prophylactic, diagnostic, and therapeutic methods must continuously be challenged through research for their effectiveness, efficiency, accessibility and quality.

7. In current medical practice and in medical research, most prophylactic, diagnostic and therapeutic procedures involve risks and burdens.

8. Medical research is subject to ethical standards that promote respect for all human beings and protect their health and rights. Some research populations are vulnerable and need special protection. The particular needs of the economically and medically disadvantaged must be recognized. Special attention is also required for those who cannot give or refuse consent for themselves, for those who may be subject to giving consent under duress, for those who will not benefit personally from the research and for those for whom the research is combined with care.

9. Research Investigators should be aware of the ethical, legal and regulatory requirements for research on human subjects in their own countries as well as applicable international requirements. No national ethical, legal or regulatory requirement should be allowed to reduce or eliminate any of the protections for human subjects set forth in this Declaration.

B. BASIC PRINCIPLES FOR ALL MEDICAL RESEARCH

10. It is the duty of the physician in medical research to protect the life, health, privacy, and dignity of the human subject.

11. Medical research involving human subjects must conform to generally accepted scientific principles, be based on a thorough knowledge of the scientific literature, other relevant sources of information, and on adequate laboratory and, where appropriate, animal experimentation.

12. Appropriate caution must be exercised in the conduct of research which may affect the environment, and the welfare of animals used for research must be respected.

13. The design and performance of each experimental procedure involving human subjects should be clearly formulated in an experimental protocol. This protocol should be submitted for consideration, comment, guidance, and where appropriate, approval to a specially appointed ethical review committee, which must be independent of the investigator, the sponsor or any other kind of undue influence. This independent committee should be in conformity with the laws and regulations of the country in which the research experiment is performed. The committee has the right to monitor ongoing trials. The researcher has the obligation to provide monitoring information to the committee, especially any serious adverse events. The researcher should also submit to the committee, for review, information regarding funding, sponsors, institutional affiliations, other potential conflicts of interest and incentives for subjects.

14. The research protocol should always contain a statement of the ethical considerations involved and should indicate that there is compliance with the principles enunciated in this Declaration.

15. Medical research involving human subjects should be conducted only by scientifically qualified persons and under the supervision of a clinically competent medical person. The responsibility for the human subject must always rest with a medically qualified person and never rest on the subject of the research, even though the subject has given consent.

16. Every medical research project involving human subjects should be preceded by careful assessment of predictable risks and burdens in comparison with foreseeable benefits to the subject or to others. This does not preclude the participation of healthy volunteers in medical research. The design of all studies should be publicly available.

17. Physicians should abstain from engaging in research projects involving human subjects unless they are confident that the risks involved have been adequately assessed and can be satisfactorily managed. Physicians should cease any investigation if the risks are found to outweigh the potential benefits or if there is conclusive proof of positive and beneficial results.

18. Medical research involving human subjects should only be conducted if the importance of the objective outweighs the inherent risks and burdens to the subject. This is especially important when the human subjects are healthy volunteers.

19. Medical research is only justified if there is a reasonable likelihood that the populations in which the research is carried out stand to benefit from the results of the research.

20. The subjects must be volunteers and informed participants in the research project.

21. The right of research subjects to safeguard their integrity must always be respected. Every precaution should be taken to respect the privacy of the subject, the confidentiality of the patient's information and to minimize the impact of the study on the subject's physical and mental integrity and on the personality of the subject.

22. In any research on human beings, each potential subject must be adequately informed of the aims, methods, sources of funding, any possible conflicts of interest, institutional affiliations of the researcher, the anticipated benefits and potential risks of the study and the discomfort it may entail. The subject should be informed of the right to abstain from participation in the study or to withdraw consent to participate at any time without reprisal. After ensuring that the subject has understood the information, the physician should then obtain the subject's freely-given informed consent, preferably in writing. If the consent cannot be obtained in writing, the non-written consent must be formally documented and witnessed.

23. When obtaining informed consent for the research project the physician should be particularly cautious if the subject is in a dependent relationship with the physician or may

consent under duress. In that case the informed consent should be obtained by a well-informed physician who is not engaged in the investigation and who is completely independent of this relationship.

24. For a research subject who is legally incompetent, physically or mentally incapable of giving consent or is a legally incompetent minor, the investigator must obtain informed consent from the legally authorized representative in accordance with applicable law. These groups should not be included in research unless the research is necessary to promote the health of the population represented and this research cannot instead be performed on legally competent persons.

25. When a subject deemed legally incompetent, such as a minor child, is able to give assent to decisions about participation in research, the investigator must obtain that assent in addition to the consent of the legally authorized representative.

26. Research on individuals from whom it is not possible to obtain consent, including proxy or advance consent, should be done only if the physical/mental condition that prevents obtaining informed consent is a necessary characteristic of the research population. The specific reasons for involving research subjects with a condition that renders them unable to give informed consent should be stated in the experimental protocol for consideration and approval of the review committee. The protocol should state that consent to remain in the research should be obtained as soon as possible from the individual or a legally authorized surrogate.

27. Both authors and publishers have ethical obligations. In publication of the results of research, the investigators are obliged to preserve the accuracy of the results. Negative as well as positive results should be published or otherwise publicly available. Sources of funding, institutional affiliations and any possible conflicts of interest should be declared in the publication. Reports of experimentation not in accordance with the principles laid down in this Declaration should not be accepted for publication.

C. ADDITIONAL PRINCIPLES FOR MEDICAL RESEARCH COMBINED WITH

MEDICAL CARE

28. The physician may combine medical research with medical care, only to the extent that the research is justified by its potential prophylactic, diagnostic or therapeutic value. When medical research is combined with medical care, additional standards apply to protect the patients who are research subjects.

29. The benefits, risks, burdens and effectiveness of a new method should be tested against those of the best current prophylactic, diagnostic, and therapeutic methods. This does not exclude the use of placebo, or no treatment, in studies where no proven prophylactic, diagnostic or therapeutic method exists.[1]

30. At the conclusion of the study, every patient entered into the study should be assured of access to the best proven prophylactic, diagnostic and therapeutic methods identified by the study.[2]

31. The physician should fully inform the patient which aspects of the care are related to the research. The refusal of a patient to participate in a study must never interfere with the patient-physician relationship.

32. In the treatment of a patient, where proven prophylactic, diagnostic and therapeutic methods do not exist or have been ineffective, the physician, with informed consent from the patient, must be free to use unproven or new prophylactic, diagnostic and therapeutic measures, if in the physician's judgment it offers hope of saving life, re-establishing health or alleviating suffering. Where possible, these measures should be made the object of research, designed to evaluate their safety and efficacy. In all cases, new information should be recorded and, where appropriate, published. The other relevant guidelines of this Declaration should be followed.

[1] Note of clarification on paragraph 29 of the WMA Declaration of Helsinki The WMA hereby reaffirms its position that extreme care must be taken in making use of a placebo-controlled trial and that in general this methodology should only be used in the absence of existing proven therapy. However, a placebo-controlled trial may be ethically acceptable, even if proven therapy is available, under the following circumstances:

– Where for compelling and scientifically sound methodological reasons its use is necessary to determine the efficacy or safety of a prophylactic, diagnostic or therapeutic method; or

– Where a prophylactic, diagnostic or therapeutic method is being investigated for a minor condition and the patients who receive placebo will not be subject to any additional risk of serious or irreversible harm.

All other provisions of the Declaration of Helsinki must be adhered to, especially the need for appropriate ethical and scientific review.

[2] Note of clarification on paragraph 30 of the WMA Declaration of Helsinki

The WMA hereby reaffirms its position that it is necessary during the study planning process to identify post-trial access by study participants to prophylactic, diagnostic and therapeutic procedures identified as beneficial in the study or access to other appropriate care. Post-trial access arrangements or other care must be described in the study protocol so the ethical review committee may consider such arrangements during its review.

9.10.2004
