# Supplementary material for: A Randomized Comparative Trial of Continued Abacavir/Lamivudine plus Efavirenz or Replacement with Efavirenz/Emtricitabine/Tenofovir DF in Hypercholesterolemic HIV-1 Infected Individuals
Source: PLoS One. 2015 Feb 6;10(2):e0116297. doi: 10.1371/journal.pone.0116297 (PMC4319732; doi:10.1371/journal.pone.0116297)
Supplement: S2 Protocol — (PDF) [file pone.0116297.s003.pdf]

Flowers Building, Granta Park
Abington, Cambridge, CB21 6GT

PROTOCOL AMENDMENT 1

Study GS-UK-177-0109

A Phase 4, Open Label, Randomized, Controlled Study to Assess the Effect on Lipid Profile of Switching from a Stable HAART Regimen of fixed dose Abacavir/Lamivudine (Kivexa) Plus Efavirenz, to Once Daily Atripla in Adult HIV-1 Infected Subjects With High Cholesterol

**Original Protocol Date:**	November, 22, 2007
**Amendment 1 Date:**	January, 22, 2008

**Rationale:**	Herein is a summary of the major changes made to the original protocol dated November, 22, 2007 and reflected in Amendment 1 dated January, 22, 2008.<br>1. Sponsor's responsibilities regarding reporting of SAEs/ SUSARs in accordance with the European Commission Clinical Trials Directive including confirmation that the Regulatory Authorities will be notified of any SUSARs.<br>Specific changes contained in Amendment 1are presented herein.

**Global Changes:**	None

**Page, Section:**	30, 7.5.1
**Original Text:**	Gilead has requirements for expedited reporting to worldwide regulatory authorities of SAEs meeting specific requirements; therefore, Gilead must be notified immediately regarding the occurrence of any SAE that occurs after the screening visit, including SAEs resulting from protocol-associated procedures performed from screening onwards.
**Revised Text:**	Gilead is required to expedite to worldwide regulatory authorities reports of Serious Adverse Events, Serious Adverse Drug Reactions or Suspected Unexpected Serious Adverse Reactions (SUSARs) in line with relevant legislation, including the European Commission Clinical Trials Directive (2001/20/EC); therefore, Gilead must be notified immediately regarding the occurrence of any SAE or SADR that occurs after the screening visit, including SAEs/SADRs resulting from protocol-associated procedures as defined in relevant legislation including 2001/20/EC, performed from screening onwards.
**Rationale:**	The MHRA requested that the protocol be amended to include the Sponsor's responsibilities as regards reporting SAEs/SUSARs in accordance with the European Directive 2001/20/EC and to confirm that the Regulatory Authorities will be notified of SUSARs according to the EU Directive 2001/20/EC.

**Page, Section:**	31, 7.5.2
**Original Text:**	An SAE may qualify for reporting to regulatory authorities if the SAE is related to the study drug. Expectedness of SAEs will be determined by Gilead using reference safety information specified in the Investigator's Brochure. All investigators will receive a formal notification describing unexpected related serious adverse events. In accordance with the EU Clinical Trials Directive (2001/20/EC), Gilead will notify the co-ordinating Ethics Committees in concerned Member States of serious adverse events that are unexpected and possibly attributable to the study drug.
**Revised Text:**	An event may qualify for reporting to regulatory authorities if it is a Serious Adverse Event, Serious Adverse Drug Reaction or Suspected Unexpected Serious Adverse Reaction (SUSAR) in line with relevant legislation, including the European Commission Clinical Trials Directive (2001/20/EC). Expectedness of SAEs will be determined by Gilead using the reference safety information specified in the Investigator's Brochure. All investigators will receive a safety letter notifying them of relevant SUSAR reports. In accordance with the European Commission Directive 2001/20/EC, Gilead will notify the relevant Ethics Committees in concerned Member States of applicable SUSARs as individual notifications or through a periodic line listing.
**Rationale:**	The MHRA requested that the protocol be amended to include the Sponsor's responsibilities as regards reporting SAEs/SUSARs in accordance with the European Directive 2001/20/EC and to confirm that the Regulatory Authorities will be notified of SUSARs according to the EU Directive 2001/20/EC.

"I have read and understand the above, and agree to this protocol amendment as written."	
Principal Investigator	Date
