# Supplementary material for: A Randomized Comparative Trial of Continued Abacavir/Lamivudine plus Efavirenz or Replacement with Efavirenz/Emtricitabine/Tenofovir DF in Hypercholesterolemic HIV-1 Infected Individuals
Source: PLoS One. 2015 Feb 6;10(2):e0116297. doi: 10.1371/journal.pone.0116297 (PMC4319732; doi:10.1371/journal.pone.0116297)
Supplement: S3 Protocol — (PDF) [file pone.0116297.s004.pdf]

Flowers Building, Granta Park
Abington, Cambridge, CB21 6GT

PROTOCOL AMENDMENT 2

Study GS-UK-177-0109

A Phase 4, Open Label, Randomized, Controlled Study to Assess the Effect on Lipid Profile of Switching from a Stable HAART Regimen of fixed dose Abacavir/Lamivudine (Kivexa) Plus Efavirenz, to Once Daily Atripla in Adult HIV-1 Infected Subjects With High Cholesterol

**Original Protocol Date:**	22 November 2007
**Amendment 1 Date:**	22 January 2008
**Amendment 2 Date:**	12 May 2008

**Rationale:**	Herein is a summary of the major changes made to the Amendment 1 protocol dated 22 January 2008 and reflected in Amendment 2 dated 12 May 2008.<br>1. Update names and abbreviations of study questionnaires; clarification of frequency of questionnaires<br>2. Update European Commission approval of Atripla<br>3. Clarification of study visit windows<br>4. Reduction of time to stop disallowed medications from 30 days to 28 days<br>5. Change to disallowed medication list<br>6. Changes to inclusion criteria<br>7. Clarification and minor corrections of statistical analysis methods<br>Specific changes contained in Amendment 2 are presented herein.

**Global Changes:**	

**Page, Section:**	Page 6, Protocol Synopsis, Diagnosis and Main Eligibility Criteria<br>Page 16, Section 4.2. Inclusion Criteria
**Original Text:**	• Plasma HIV RNA < 50 copies/mL\L ≥ 12 weeks prior to screening<br>• Documented confirmed total cholesterol ≥ 5.2 mmol/L for last two consecutive testings (at least 4 weeks apart) with last result ≤ 4 weeks prior to Screening

**Revised Text:**	• Undetectable plasma HIV-1 RNA (<50 copies/mL) at Screening and ≥ 12 weeks prior to Screening.<br>• Documented confirmed raised total cholesterol ≥ 5.2 mmol/L for last two consecutive tests (at least 4 weeks apart) prior to Screening and fasting total cholesterol ≥ 5.2 mmol/L at Screening.
**Rationale:**	Clarification that the subject's viral load needs to be < 50 copies/ml at the screening visit as well as being undetectable for at least 12 weeks before screening.<br>A fasting total cholesterol ≥ 5.2 mmol/L needs to be obtained at the Screening visit to ensure that subjects are not enrolled that subsequently do not have the required cholesterol level at Baseline.

**Page, Section:**	Page 6, Protocol Synopsis, Study Procedures/ Frequency
**Original Text:**	The following will be performed at all visits (Screening, Baseline, Weeks 4, 12, 16 and 24):<br>• Hematology, serum chemistry and urinalysis tests<br>• Plasma HIV RNA (viral load)<br>• CD4/CD8 cell count and percentage<br>• Calculated creatinine clearance<br>• Vital signs, weight and physical examination<br>• Pregnancy test for women of childbearing potential
**Revised Text:**	The following will be performed at all visits (Screening, Baseline, Weeks 4, 12, 16 and 24):<br>• Hematology, serum chemistry and urinalysis tests<br>• Plasma HIV RNA (viral load)<br>• CD4/CD8 cell count and percentage<br>• Calculated creatinine clearance<br>• Vital signs, weight and physical examination<br>• Pregnancy test for women of childbearing potential<br>• Fasting lipid profile measurements. These will include total cholesterol (TC), high density and low density lipoproteins (HDL, LDL), triglycerides (TG) and cholesterol ratios<br>• Fasting glucose
**Rationale:**	Fasting glucose and lipids to be performed at screening visit also.

**Page, Section:**	Page 7, Protocol Synopsis, Study Procedures/ Frequency
**Original Text:**	Patient Preference of Medicine (POM) questionnaire at Week 4, Week 12, Week 16, and Week 24.

**Revised Text:**	Patient Preference of Medicine (POM) questionnaire at Week 4 for Group 1 subjects and Week 16 for Group 2 subjects.
**Rationale:**	The POM questionnaire is completed by both groups 4 weeks after they switch treatment.

**Page, Section:**	Page 10, Glossary of Abbreviations and Definition of Terms
**Original Text:**	NA
**Revised Text:**	PERC: Perceived Ease of Regimen for Condition<br>POM: Preference of Medication<br>VAS: Visual Analogue Scale
**Rationale:**	Addition of questionnaire abbreviations to glossary of abbreviations

**Page, Section:**	Page12, Section 1.2. EFV/FTC/TDF Fixed-Dose Combination Tablets (Atripla)
**Original Text:**	In the European Union a Marketing Authorization Application (MAA) has been filed with the European Medicines Agency (EMEA) and is undergoing review. The formulation received a positive opinion from the CHMP on 18th October 2007, with a recommended indication for the treatment of HIV-1 infected adults with virologic suppression to HIV-1 RNA levels of < 50 copies/ml on their current antiretroviral combination therapy for more than 3 months. Patients must not have experienced virological failure on any prior antiretroviral therapy and must be known not to have harboured virus strains with mutations conferring significant resistance to any of the three components contained in Atripla prior to initiation of their first antiretroviral treatment regimen. The CHMP Opinion on Atripla is currently being considered by the European Commission which is empowered to grant marketing authorizations in the European Union.
**Revised Text:**	In the European Union the formulation received a positive opinion from the CHMP on 18 October 2007 and European Commission decision on 13 December 2007. The approved EU indication is for the treatment of HIV-1 infected adults with virologic suppression to HIV-1 RNA levels of < 50 copies/mL on their current antiretroviral combination therapy for more than 3 months. Patients must not have experienced virological failure on any prior antiretroviral therapy and must be known not to have harboured virus strains with mutations conferring significant resistance to any of the three components contained in Atripla prior to initiation of their first antiretroviral treatment regimen.
**Rationale:**	Updated information on status of the approval of Atripla

**Page, Section:**	Page 15, Figure 3-1. Study Schema
**Original Text:**	12 weeks<br>24 weeks
**Revised Text:**	12 weeks (end of phase 1)<br>24 weeks (end of phase 2)
**Rationale:**	Addition of wording to clarify that the study is comparing two 12 week periods of treatment with Atripla. Week 12 is the end of the 12 week period for Phase 1 and the start of the 12 week period for Phase 2.

**Page, Section:**	Page 16, Section 4.2. Inclusion Criteria
**Original Text:**	Hepatic Total Bilirubin ≤ 1.5mg/dL
**Revised Text:**	Hepatic Total Bilirubin ≤ 22 umol/L
**Rationale:**	Units changed to reflect European standard

**Page, Section:**	Page 18, Section 4.3. Exclusion Criteria
**Original Text:**	Administration of any of these medications must be discontinued at least 30 days prior to the Baseline visit
**Revised Text:**	Administration of any of these medications must be discontinued at least 28 days prior to the Baseline visit
**Rationale:**	Subjects that are taking disallowed medications at the Screening visit, that are otherwise eligible for the study, can be requested at screening to stop taking the disallowed medication, providing the Investigator feels this is in the subject's best interest, to enable the subject to attend the Baseline visit 28 days later.

**Page, Section:**	Page 23, Section 6.2.1. Screening Visit
**Original Text:**	• Chemistry profile: albumin, AST, ALT, direct bilirubin, total bilirubin, creatinine, potassium, phosphate, glucose, calcium and amylase, CrCl (calc), sodium, bicarbonate, chloride
**Revised Text:**	• Chemistry profile: albumin, AST, ALT, direct bilirubin, total bilirubin, creatinine, potassium, phosphate, glucose, calcium and amylase, CrCl (calc), sodium, bicarbonate, chloride<br>• Fasting lipid profile – TC, LDL, HDL, non-HDL, TG, cholesterol ratios<br>• Fasting glucose
**Rationale:**	Fasting glucose and lipids to be performed at screening visit also.

**Page, Section:**	Page 24, Section 6.2.2. Baseline Assessments
**Original Text:**	• Acceptability questionnaires:<br>- Treatment Satisfaction and Symptoms Questionnaire, Appendix 6<br>- HAART Intrusiveness Scale (m-HIS), Appendix 8<br>• Adherence questionnaire (Perceived Ease of Adherence (PERC) and Visual Analogue Scale (VAS), Appendix 9

**Revised Text:**	• Acceptability questionnaires:<br>- Treatment Satisfaction and Symptoms (TSS) questionnaire, Appendix 6<br>- HAART Intrusiveness Scale (m-HIS), Appendix 8<br>- Perceived Ease of Regimen for Condition (PERC) questionnaire, Appendix 9<br>• Adherence questionnaire:<br>- Visual Analogue Scale (VAS) adherence questionnaire, Appendix 9
**Rationale:**	PERC questionnaire and VAS adherence questionnaire are two questionnaires in one appendix, PERC is for acceptability and VAS is for adherence.

**Page, Section:**	Page 24, Section 6.3. Treatment Assessments
**Original Text:**	All study visits should be completed within ± 5days based on the baseline visit
**Revised Text:**	All study visits after switching to Atripla should be completed within ± 5 days based on the switch to Atripla visit (Baseline visit for Group 1 and Week 12 visit for Group 2). For Group 2, the study visits up to to Week 12 should be completed within ± 5 days based on the Baseline visit.
**Rationale:**	Week 12 to Week 24 for Group 2 subjects are required to replicate the Baseline to Week 12 visit for Group 1 subjects. This will enable the study to truly compare the Week 12 outcome for Group 1 subjects with the Week 24 outcome for Group 2 subjects and hence assess if there is a confirmation of the Week 12 effect.

**Page, Section:**	Page 25, Section 6.3. Treatment Assessments
**Original Text:**	• Acceptability questionnaires:<br>- Treatment Satisfaction and Symptoms Questionnaire, Appendix 6<br>- HAART Intrusiveness Scale (m-HIS), Appendix 8<br>• Adherence questionnaires:<br>- Patient Preference of Medication (POM) questionnaire, Appendix 5 (Week 4 and Week 16 for switched patients only)<br>- Perceived Ease of Adherence (PERC) and Visual Analogue Scale (VAS), Appendix 9
**Revised Text:**	• Acceptability questionnaires:<br>- Patient Preference of Medication (POM) questionnaire, Appendix 5 (Week 4 and Week 16 for switched patients only)<br>- Treatment Satisfaction and Symptoms (TSS) questionnaire, Appendix 6<br>- HAART Intrusiveness Scale (m-HIS), Appendix 8<br>- Perceived Ease of Regimen for Condition (PERC) questionnaire, Appendix 9<br>• Adherence questionnaire:<br>- Visual Analogue Scale (VAS) adherence questionnaire, Appendix 9

**Rationale:**	PERC questionnaire and VAS adherence questionnaire are two questionnaires in one appendix, PERC is for acceptability and VAS is for adherence.<br>POM is an acceptability questionnaire.

**Page, Section:**	Page 26, Section 6.7. Criteria for Discontinuation of Study Treatment
**Original Text:**	NA
**Revised Text:**	Subjects who discontinue study treatment prior to Week 24 should have an Early Termination Visit within 72 hours of the last dose of study drug. Additionally, subjects will be contacted 30 days after the last study visit for a follow-up assessment.
**Rationale:**	Addition of procedure for early termination visit as this was missed off the previous version.

**Page, Section:**	Page 30, Section 7.4. Overdose
**Original Text:**	Any accidental or intentional overdose (any increase in frequency or dosage of study medication that exceeds what is mandated by the protocol), misuse or abuse of study medication as well as any Gilead product taken as a concomitant medication, whether suspected or confirmed, and whether or not associated with an adverse experience, must be reported using the SAE form and forwarded within 24 hours to Gilead Drug Safety and Public Health. The procedure for reporting is the same as an SAE, as outlined in Section 7.5. Although the procedure for reporting all overdoses will be the same as that for reporting SAEs, an overdose will be considered an SAE only if any of the seriousness criteria are met. Any clinical sequelae in association with the overdose should be reported as an AE (as outlined in Section 7.1) or SAE (as outlined in Section 7.3) along with the overdose. Details of signs or symptoms, clinical management and outcome should be reported if available.
**Revised Text:**	Any accidental or intentional overdose (any increase in frequency or dosage of study medication that exceeds what is mandated by the protocol), misuse or abuse of study medication as well as any Gilead product taken as a concomitant medication, whether suspected or confirmed, and whether or not associated with an adverse experience, must be reported using the Gilead Drug Safety and Public Health (DSPH) Clinical Overdose Report form and forwarded within 24 hours to Gilead Drug Safety and Public Health. The procedure for reporting is the same as an SAE, as outlined in Section 7.5. Although the procedure for reporting all overdoses will be the same as that for reporting SAEs, an overdose will be considered an SAE only if any of the seriousness criteria are met. Any clinical sequelae in association with the overdose should be reported as an AE (as outlined in Section 7.1) or SAE (as outlined in Section 7.3) along with the overdose. Details of signs or symptoms, clinical management and outcome should be reported if available.
**Rationale:**	This has been updated to use the new Overdose Report form to report overdoses instead of the SAE form

**Page, Section:**	Pages 36–40, Section 8. Statistical Considerations
**Original Text:**	Total cholesterol
**Revised Text:**	Total fasting cholesterol
**Rationale:**	Correction of total cholesterol to total fasting cholesterol throughout this section to ensure consistency with the rest of the protocol

**Page, Section:**	Page 37, Section 8.4.1. Analysis Sets
**Original Text:**	**Intent to Treat (ITT)**<br>ITT is the primary analysis set for the analyses of the efficacy endpoints and major deviation for ITT set is considered as HIV RNA > 1000 copies/mL at screening visit.<br>**Modified Intent to Treat (MITT)**<br>MITT is the primary analysis set for the analyses of the primary endpoint (change in total cholesterol) and major deviation for MITT set is considered as total cholesterol < 4.2 mmol/L at the screening visit.

**Revised Text:**	**Intent to Treat (ITT)**<br>ITT is the primary analysis set for the analyses of the efficacy endpoints and major deviation for ITT set is considered as HIV RNA > 1000 copies/mL at baseline visit.<br>**Modified Intent to Treat (MITT)**<br>MITT is the primary analysis set for the analyses of the primary endpoint (change in total cholesterol) and major deviation for MITT set is considered as total fasting cholesterol < 4.2 mmol/L at the baseline visit.
**Rationale:**	This was specified as screening visit in error.

**Page, Section:**	Page 37, Section 8.4.2. Data Handling Conventions
**Original Text:**	Total cholesterol at Week 24: For Subjects receiving Atripla who have missing cholesterol at Week 24 the missing results will be imputed by last observed post-baseline value. For subjects receiving KVX+EFV who have missing cholesterol at study Week 24 the missing result will be imputed by last observed post-switch (post-Week 12) result.
**Revised Text:**	Total cholesterol at Week 24: For subjects randomized to Atripla at baseline visit who have missing cholesterol at Week 24 the missing results will be imputed by last observed post-baseline value. For subjects randomized to KVX+EFV at baseline visit who have missing cholesterol at study Week 24 the missing result will be imputed by last observed post-switch (post-Week 12) result.
**Rationale:**	Clarification of wording

**Page, Section:**	Page 39, Section 8.6.2. Analysis of Secondary Endpoints
**Original Text:**	**Analysis of Safety Endpoints**<br>All safety data collected on or after the date that study drug was first dispensed, up to the date of last dose of study drug plus 30 days, will be summarized by treatment group (according to the study drug received).
**Revised Text:**	**Analysis of Safety Endpoints**<br>All safety data collected on or after the first dose date of study drug, up to the date of last dose of study drug plus 30 days, will be summarized b y treatment group (according to the study drug received).
**Rationale:**	Safety analysis corrected to only reflect the period that study drug was received.

**Page, Section:**	Page 48, Appendices<br>Page 82, Appendix 9
**Original Text:**	Appendix 9. Perceived Ease of Adherence (PERC) and Visual Analogue Scale (VAS) Questionnaire
**Revised Text:**	Appendix 9. Perceived Ease of Regimen for Condition (PERC) Questionnaire and Visual Analogue Scale (VAS) adherence Questionnaire
**Rationale:**	Title did not correctly match acronym for PERC. Visual Analogue Scale is an adherence questionnaire which was not clear in the original title.

**Page, Section:**	Pages 50–51, Appendix 2. Study Procedures Table
**Original Text:**	24 Weeks/Early Term
**Revised Text:**	24 Weeks/Early Term[h]
**Rationale:**	Footnote added to give further guidance for early termination visit

**Page, Section:**	Pages 50–51, Appendix 2. Study Procedures Table
**Original Text:**	NA
**Revised Text:**	h Early term visit to be performed within 72 hours of last dose of study drug
**Rationale:**	Footnote added to give further guidance for early termination visit

**Page, Section:**	Page 50, Appendix 2. Study Procedures Table
**Original Text:**	Vital signs
**Revised Text:**	Vital Signs[g]
**Rationale:**	Footnote added to give further guidance for vital signs

**Page, Section:**	Pages 50–51, Appendix 2. Study Procedures Table
**Original Text:**	Screening<br>Day ≤ –28
**Revised Text:**	Screening<br>Day ≥ –28
**Rationale:**	The baseline visit should occur within 28 days of the screening visit. This was not correctly specified.

**Page, Section:**	Page 51, Appendix 2. Study Procedures Table
**Original Text:**	NA
**Revised Text:**	g Vital signs include blood pressure and heart rate. Blood pressure to be taken in sitting position.
Rationale:	Footnote added to give further guidance for vital signs.

**Page, Section:**	Page 51, Appendix 2. Study Procedures Table
**Original Text:**	POM Adherence Questionnaire
**Revised Text:**	POM Acceptability Questionnaire
**Rationale:**	The POM questionnaire is an acceptability questionnaire not an adherence questionnaire.

**Page, Section:**	Page 51, Appendix 2. Study Procedures Table
**Original Text:**	PERC Adherence and VAS Questionnaire
**Revised Text:**	VAS Adherence Questionnaire
**Rationale:**	Incorrect labeling of PERC and VAS questionnaire

**Page, Section:**	Page 51, Appendix 2. Study Procedures Table
**Original Text:**	Acceptability Questionnaires (TSS and m-HIS)
**Revised Text:**	Acceptability Questionnaires (TSS, m-HIS and PERC)
**Rationale:**	The PERC questionnaire is an acceptability questionnaire not an adherence questionnaire

**Page, Section:**	Page 76, Appendix 5. Patient Preference of Medicine (POM)
**Original Text:**	For Treatment Group 1 (switch to Atripla tablet) Patients Only
**Revised Text:**	For patients who have just switched to Atripla tablet
**Rationale:**	The POM questionnaire is completed by both groups 4 weeks after they switch treatment.

**Page, Section:**	Page 76, Appendix 5. Patient Preference of Medicine (POM)
**Original Text:**	How easy did you find it to follow you current HIV medication regimen?
**Revised Text:**	How does your current medication compare to the previous HAART medicines your doctor prescribed for your HIV infection?
**Rationale:**	The questionnaire wording was changed to reflect the standard wording used across all Gilead studies using this questionnaire.

**Page, Section:**	Page 79, Appendix 7. Disallowed Medications
**Original Text:**	Atorvastatin<br>Pravastatin
**Revised Text:**	~~Atorvastatin~~<br>~~Pravastatin~~
**Rationale:**	Atorvastatin and Pravastatin have been removed from the disallowed medications list as these are medications commonly taken by HIV patients with high cholesterol in normal clinical practice. Potential study subjects will have been stable on these for at least 12 weeks prior to entering the study in order to meet the entry criteria. Subjects will not, however, be permitted to initiate these drugs during the study.

**Page, Section:**	Page 81, Appendix 8. HAART Intrusiveness Scale (m-HIS)
**Original Text:**	MH4: Is too complex to stick to<br>MH4: Restrict my ability to travel<br>MH5: Interferes with my work<br>MH6: Makes it harder to meet friends<br>MH7: Interferes with my relationships<br>MH8: Is difficult to swallow<br>MH9: Is difficult to remember to take on time
**Revised Text:**	MH4: Is too complex to stick to<br>MH5: Restricts my ability to travel<br>MH6: Interferes with my work<br>MH7: Makes it harder to meet friends<br>MH8: Interferes with my relationships<br>MH9: Is difficult to swallow<br>MH10: Is difficult to remember to take on time
**Rationale:**	The numbering of question MH4 was used twice. The questionnaire has been re-numbered from MH4 to MH10 to ensure the numbering is consistent. Also, for MH5, Restrict has been changed to Restricts for better grammar.

**Page, Section:**	Page 82, Appendix 9. Perceived Ease of Regimen for Condition (PERC) Questionnaire and Visual Analogue Scale (VAS) Adherence Questionnaire
**Original Text:**	Perceived Ease of Adherence (PERC) and Visual Analogue Scale (VAS)
**Revised Text:**	Perceived Ease of Regimen for Condition (PERC) questionnaire
**Rationale:**	PERC questionnaire and VAS adherence questionnaire are two questionnaires in one appendix. This title should be for the PERC only.

“I have read and understand the above, and agree to this protocol amendment as written.”	
Principal Investigator	Date
