# Supplementary material for: A Randomized Comparative Trial of Continued Abacavir/Lamivudine plus Efavirenz or Replacement with Efavirenz/Emtricitabine/Tenofovir DF in Hypercholesterolemic HIV-1 Infected Individuals
Source: PLoS One. 2015 Feb 6;10(2):e0116297. doi: 10.1371/journal.pone.0116297 (PMC4319732; doi:10.1371/journal.pone.0116297)
Supplement: S4 Protocol — (PDF) [file pone.0116297.s005.pdf]

Flowers Building, Granta Park
Abington, Cambridge, CB21 6GT

PROTOCOL AMENDMENT 3

Study GS-UK-177-0109

A Phase 4, Open Label, Randomized, Controlled Study to Assess the Effect on Lipid Profile of Switching from a Stable HAART Regimen of fixed dose Abacavir/Lamivudine (Kivexa) Plus Efavirenz, to Once Daily Atripla in Adult HIV-1 Infected Subjects With High Cholesterol

**Original Protocol Date:**	22 November 2007
**Amendment 1 Date:**	22 January 2008
**Amendment 2 Date:**	12 May 2008
**Amendment 3 Date:**	15 December 2008

**Rationale:**	Herein is a summary of the major changes made to the Amendment 2 protocol dated 12 May 2008 and reflected in Amendment 3 dated 15 December 2008.<br>1. Addition of Ireland as a new country<br>2. Change in DSPH contact details<br>3. Removal of the requirement to collect partner pregnancies<br>4. Update of Declaration of Helsinki to latest version<br>Specific changes contained in Amendment 3 are presented herein.

**Global Changes:**	

**Page, Section:**	5, Protocol Synopsis
**Original Text:**	• Approximately 10 centers in the United Kingdom
**Revised Text:**	• Approximately 18 centers in the United Kingdom and Ireland
**Rationale:**	Addition of Ireland as a new country and update in total number of centers

**Page, Section:**	31, Section 7.5.1
**Original Text:**	Name: Rie Devert, RN MSN<br>Title: Associate Manager, DSPH<br>Phone: 01223 897500<br>Fax: 01223 897290<br>Email: csafety@gilead.com

**Revised Text:**	Name: Sarah Charles<br>Title: Senior Manager, DSPH<br>Phone: 01223 897500<br>Fax: 01223 897290<br>Email: csafety@gilead.com
**Rationale:**	Update to contact name for DSPH.

**Page, Section:**	34, Section 7.8
**Original Text:**	The investigator should report all pregnancies that occur in female subjects as well as female partners of male subjects to Gilead DSPH within 24 hours of becoming aware of the pregnancy, and up to 30 days after the last dose of study drugs.
**Revised Text:**	The investigator should report all pregnancies that occur in female subjects to Gilead DSPH within 24 hours of becoming aware of the pregnancy, and up to 30 days after the last dose of study drugs.
**Rationale:**	Revised wording to reflect that the collection of partner pregnancies is no longer required as Gilead has assessed the impact of Atripla on partner pregnancies and have no evidence that offspring can be affected due to paternal exposure.

**Page, Section:**	84, Appendix 11
**Original Text:**	World Medical Association Declaration of Helsinki, 2004
**Revised Text:**	World Medical Association Declaration of Helsinki, 2008
**Rationale:**	Update to include latest version of Declaration of Helsinki.

"I have read and understand the above, and agree to this protocol amendment as written."	
Principal Investigator	Date
